# Effectiveness and safety of Maxing Shigan Decoction for community-acquired pneumonia: a systematic review and meta-analysis of randomized controlled trials

**DOI:** 10.3389/fmed.2025.1639027

**Published:** 2025-08-29

**Authors:** Yutong Ling, Xuehan Liu, Bingrui Zhang, Qionghua Xiao, Zhihao Shuai, Han Bai, Rui Cai, Shuangsang Li, Mingyi Yuan, Yanxia Zhang

**Affiliations:** ^1^Dongfang Hospital, Beijing University of Chinese Medicine, Beijing, China; ^2^Center for Evidence-Based Chinese Medicine, Beijing University of Chinese Medicine, Beijing, China; ^3^Dongzhimen Hospital, Beijing University of Chinese Medicine, Beijing, China; ^4^Graduate School, Henan University of Chinese Medicine, Zhengzhou, China; ^5^China-Japan Friendship Clinical Medical College, Beijing University of Chinese Medicine, Beijing, China; ^6^China Academy of Chinese Medicine Sciences Guang’anmen Hospital, Beijing, China

**Keywords:** traditional Chinese medicine, Maxing Shigan Decoction, community-acquired pneumonia, systematic review, meta-analysis

## Abstract

**Background and Objective:**

Community-acquired pneumonia (CAP) is an acute lung infection disease with high morbidity and mortality. The treatment of CAP has become more and more challenging due to the gradual increase of antibiotic resistance and adverse events. Relevant evidence indicates that Maxing Shigan Decoction (MXSG) may play a unique therapeutic advantage. Our aim is to evaluate the overall effectiveness and safety of MXSG for CAP.

**Methods:**

Eight databases (PubMed, Embase, the Cochrane Library, CNKI, Wanfang, VIP, Yiigle, and Sinomed) were searched from their inception to January 20, 2025. Randomized controlled trials evaluating the effectiveness and safety of MXSG alone or in combination with conventional western medicine (WM) for CAP were included. We conducted meta-analysis by RevMan 5.4 software or just performed qualitative analysis.

**Results:**

We included 81 RCTs with 6682 participants in total. Compared with western medicine (WM) alone, MXSG plus WM showed a more beneficial effect on reducing the duration of fever (MD = −1.58 days, 95% CI: −1.88 to −1.29, *p* < 0.00001), cough (MD = −2.30 days, 95% CI: −2.61 to −1.99, *p* < 0.00001), phlegm (MD = −2.40 days, 95% CI: −2.56 to −2.23, *p* < 0.00001), dyspnea (MD = −2.11 days, 95% CI: −2.73 to −1.49, *p* < 0.00001), pulmonary crepitation (MD = −2.13 days, 95% CI: −2.47 to −1.79, *p* < 0.00001) and length of hospitalization (MD = −1.38 days, 95% CI: −2.54 to −0.23, *p* = 0.02). Furthermore, MXSG plus WM was significantly superior to WM in promoting the absorption of lung inflammation (MD = −3.31 days, 95% CI: −4.17 to −2.46, *p* < 0.00001) and improving forced expiratory volume in the first second (MD = 0.54 L, 95% CI: 0.21 to 0.87, *p* = 0.001). The incidence of adverse events was 3.60% in MXSG plus WM group and 5.38% in WM group, but the difference was not significant (*p* = 0.06).

**Conclusion:**

Moderate or low certainty of evidence suggested that compared with WM alone, MXSG combined with WM may have potential effectiveness on relieving the clinical symptoms, promoting the absorption of lung inflammation, improving lung function, and reducing hospitalization length with a good safety for patients with CAP. In the future, high-quality double-blind RCTs should be required to confirm the effectiveness and safety on CAP.

**Systematic review registration:**

https://www.crd.york.ac.uk/prospero/display_record.php?ID=CRD42023404693, identifier CRD42023404693.

## 1 Introduction

Community-acquired pneumonia (CAP) is an acute inflammation of lung parenchyma which occurs outside the hospital, including pneumonia that develops during the incubation period after hospital admission. Common clinical manifestations of CAP include fever, cough, expectoration, dyspnea, chest distress, chest pain, and localized auscultatory abnormalities (1). CAP is one of the leading causes of death from infectious diseases with high morbidity and mortality in all ages (2). In the United States, more than 1.5 million adults are hospitalized for CAP each year, 100,000 deaths of inpatients occur during hospitalization and approximately a third of hospitalization patients with CAP die within 1 year (1, 3). A study in 2020 showed that the annual incidence of CAP in China was about 713 patients per 100,000 people (4). Besides, CAP also becomes one of the major burdens on healthcare resources with high medical cost. For CAP inpatients, the mean healthcare cost is 11148 dollars in simple cases and 51219 dollars in complicated cases (5). CAP can be caused by a variety of pathogenic microorganisms such as bacteria, viruses, fungi, and other atypical pathogens. *Streptococcus pneumoniae*, human rhinovirus, and influenza virus are the most frequently identified pathogens (2). Other common pathogens include *Staphylococcus aureus*, *Mycoplasma pneumoniae*, *Legionella*, and Enterobacteriaceae (2).

The conventional treatments for CAP involve anti-infective treatment and symptomatic supportive treatment. Prior to pathogen identification, antibiotics should be selected according to the patient age, comorbidities, risk factors, severity of disease, antibiotic allergies, the most possible pathogen, and local epidemiological patterns (2). Referring to the guideline of American Thoracic Society and Infectious Diseases Society of America, amoxicillin, doxycycline, or a macrolide are recommended for CAP patients without comorbidities or risk factors (6). For patients with comorbidities, the choices of antibiotics involve a monotherapy with a respiratory fluoroquinolone as well as a combination therapy of amoxicillin/clavulanate or a cephalosporin and macrolide or doxycycline (6). However, the irrational use of antibiotics may result in potential adverse events (7). And the increasing antibiotic resistance has brought great difficulty to empiric treatment of CAP and has become a significant global public health problem (8). In this context, traditional Chinese medicine (TCM) has distinctive features and obvious advantages. Clinical evidence indicated that TCM alone could reduce antibiotic utilization in the treatment of none-severe CAP (9). Furthermore, the combination of TCM and western medicine may decrease the treatment failure rate and mortality in treating severe CAP (9).

Maxing Shigan Decoction (MXSG) is one of the classical TCM formulations which has been applied to treating respiratory infectious diseases for thousands of years. Originally documented in Treatise on Febrile and Miscellaneous Diseases, MXSG consists of Ephedrae Herba (Mahuang, *Ephedra sinica* Stapf), Armeniacae Semen Amarum (Xingren, Semen Armeniacae Amarum), Gypsum Fibrosum (Shigao, Gypsum Fibrosum), Radix Glycyrrhizae (Gancao, *Glycyrrhiza uralensis* Fisch.). The combination of these four kinds of Chinese herbal medicine plays the role of clearing heat, freeing lung and calming panting, which is suitable for the TCM syndrome of lung heat congestion characterized by fever, cough, asthma, thirst, thin white or yellow tongue coating, and slippery rapid pulse. Modern pharmacological studies have corroborated that MXSG may have antipyretic, anti-inflammatory, antibacterial, antiviral, and immunomodulatory effects (10–12). For instance, a preclinical study demonstrated the antiviral and anti-inflammatory pharmacodynamic functions and the mechanism of MXSG by network pharmacology and in vitro experimental verification, which also indicated the positive role of MXSG in combating COVID-19 (10). In another in vitro model of *Mycoplasma pneumoniae* infection in A549 cell culture, the experiment results revealed that MXSG played anti-inflammatory action by reducing NLRP3, pro-IL-1β, Caspase-1, pro-Caspase-1, and GSDMD-N (11).

Numerous clinical trials have provided evidence demonstrating positive therapeutic effect of MXSG on CAP (13, 14). Furthermore, Several Chinese clinical guidelines also recommended MXSG as a complementary medicine in CAP treatment (9, 15). In recent years, growing medical researchers have developed a large number of Chinese herbal formulations based on MXSG by adjusting composition and dosage of the original formulation, which brings more possibility and variety into clinical research. Therefore, this review aims to evaluate the total effectiveness and safety of MXSG for CAP by systematic review and try to explore the potential factors which may affect the therapeutic effect.

## 2 Materials and methods

### 2.1 Study registration

This systematic review was conducted referring to the Preferred Reporting Items for Systematic Reviews and Meta-Analyses (PRISMA) 2020 (16) and Cochrane Handbook for Systematic Reviews of Interventions (17). We have registered a protocol of this review in PROSPERO (CRD42023404693).

### 2.2 Eligibility criteria

#### 2.2.1 Type of studies

All design modes of randomized controlled trials (RCTs) evaluating the effectiveness and safety of MXSG in the treatment of CAP were involved in this review, regardless of source or country. Duplicate publications were also included. However, we would only extract the data in publication with more complete information.

#### 2.2.2 Type of participants

Participants meeting the diagnostic criteria for CAP (6) were eligible for enrollment: (1) community-acquired onset; (2) new chest imaging findings demonstrating patchy infiltrates, lobar or segmental consolidation, ground-glass opacities, or interstitial changes; and (3) at least one of the following clinical manifestations: (i) new-onset cough, sputum production, exacerbation of pre-existing respiratory symptoms, chest pain, dyspnea, or hemoptysis; (ii) fever; (iii) signs of pulmonary consolidation or the presence of wet rales on auscultation; or (iv) peripheral white blood cell count > 10 × 10^9^/L or <4 × 10^9^/L. This diagnosis requires the exclusion of alternative conditions including tuberculosis, lung tumors, non-infectious interstitial lung diseases, pulmonary edema, atelectasis and pulmonary embolism. Besides, patients with comorbidities such as tumors, tuberculosis, bronchiectasis, and other serious diseases were excluded. There were no restrictions on age, gender, or race of participants.

#### 2.2.3 Type of interventions

Interventions involved oral MXSG or modified MXSG (including Ephedrae Herba, Armeniacae Semen Amarum, Gypsum Fibrosum and Radix Glycyrrhizae), without limitations on dosage, frequency or dosage form. The treatment group received MXSG alone or in combination with conventional western medicine (WM). WM included antibiotics, antipyretic, antitussive, expectorant, antiasthmatic and other symptomatic treatment. Control group received either no intervention, placebo, waiting-list management or WM alone. It should be noted that if the WM in both groups were different, the study was excluded. The control group could not contain TCM therapy.

#### 2.2.4 Type of comparisons

The following comparisons were included in this review: MXSG vs. placebo, MXSG vs. WM, MXSG plus WM vs. WM, and MXSG+WM1 vs. WM1+WM2.

#### 2.2.5 Type of outcome measures

The included studies were required to report at least one of the following outcomes:

(1) Main outcomes

Resolution time of symptoms (defined as the duration from treatment initiation until complete alleviation of target symptoms): Resolution time of fever, cough, phlegm, pulmonary crepitations, dyspnea, and chest pain.

Adverse events: The specific adverse events that occurred during treatment and the incidence of adverse events were recorded.

(2) Additional outcomes

Laboratory indicators: White blood cell, C-reactive protein and procalcitonin.

Improvement of chest radiographs: Improvement rate of chest radiograph (defined as the proportion of patients demonstrating improvement on chest imaging after treatment), the absorption time of lung inflammation (the duration from treatment initiation until complete clearance of pulmonary inflammatory lesions).

Lung function: Forced vital capacity (FVC), forced expiratory volume in the first second (FEV1) and peak expiratory flow (PEF).

Length of hospital stay.

All-cause mortality: The proportion of deceased patients to the total enrolled cohort during the study.

### 2.3 Search strategy

Eight databases were searched from their inception to January 20, 2025, including Chinese National Knowledge Infrastructure (CNKI), Wanfang Database, Chongqing VIP Database (VIP), Yiigle Database, Chinese Biomedical Literature Database (Sinomed), Cochrane Library, PubMed and Embase. We also retrieved the reference lists of included studies, relevant systematic reviews and clinical trial registers to find studies meeting the inclusion criteria. The detailed search strategies were in Supplementary File 1.

### 2.4 Data selection and extraction

Two reviewers independently screened titles and abstracts to identify potentially relevant studies using NoteExpress3.6.0 software and further judged possible relevant studies by reading the full text. Disagreements were resolved through discussion with a third author.

After the selection process, six reviewers independently performed data extraction by using pretested Excel data extraction forms. The data to extract includes (1) basic information of the eligible studies (such as author, year of publication, and sample size) (2) characteristics of participants (such as age and gender) (3) details of the interventions (such as dose, dosage form, and course) (4) outcome data.

### 2.5 Risk of bias

Five reviewers independently used the Cochrane Risk of Bias 2 tool (17) to evaluate the quality of included RCTs based on the following five items: randomization process, deviations from intended interventions, missing outcome data, measurement of the outcome and selection of the reported result. We reported all the risks of bias described above, and judged each item from three levels: “high risk,” “low risk,” and “some concerns.” Each RCT was evaluated an overall bias by two reviewers independently. Any disagreement was resolved by consensus or with the discussion of a third review author.

### 2.6 Data syntheses

Meta-analysis was performed when studies had homogeneity. And qualitative analysis would be used when both subgroup analysis and sensitivity analysis could not explain the source of heterogeneity. RevMan 5.4 software was applied for meta-analysis. We evaluated effect size by using relative risk (RR) with 95% CI for dichotomous data, mean difference (MD) or standardized mean difference (SMD) with 95% CI for continuous data.

Because of the variation in TCM treatments (including composition, dosage, dosage form, and administration frequency), there was considerable clinical heterogeneity among the included studies. Therefore, we chose the random effects model to pool the overall effects. Heterogeneity between studies was evaluated using the statistic I^2^. When there was substantial heterogeneity (I^2^ > 50%), subgroup analysis or sensitivity analysis would be conducted to understand the source of heterogeneity or we just conducted qualitative integrated description.

Sensitivity analysis was performed to test the robustness of the results by excluding the included studies one by one to see if there were clinical differences. Funnel plot and Egger’s test were conducted to evaluate publication bias if there were at least 10 RCTs for certain outcome.

### 2.7 Subgroup analysis

Subgroup analysis would be performed when there was substantial heterogeneity (I^2^ > 50%). In consideration of participants’ characteristics, TCM therapy, and other clinical diversity, we would try to conduct subgroup analysis according to the following clinical factors: (1) age of participants (patients aged under 14 years old were regarded as children, in the range of 14–65 years old as adults, and over 65 years old as elders; (2) severity of CAP; (3) flavored quantity of Chinese herbal medicine (the flavored quantity referred to the modification amount of Chinese herbal medicine except for Ephedrae Herba, Semen Armeniacae Amarum, Gypsum Fibrosum and Radix Glycyrrhizae in Modified MXSG); (4) whether to take syndrome differentiation and treatment (syndrome differentiation and treatment meant researchers would add or cut several Chinese herbal medicine in the formulation according to the specific symptom or syndrome of each participant).

### 2.8 Trial sequential analysis

To reduce type I (false positive) and type II (false negative) errors, we conducted trial sequential analysis (TSA) using TSA Viewer version 0.9.5.10 (Copenhagen: Copenhagen trial Unit) to control the risk of random errors and estimated the required sample size for robust meta-analysis conclusion. We defined the risk of type I errors as 5% and risk of type II errors as 20%. For dichotomous outcomes, we applied 33.09% relative risk reduction (RRR) based on the results of previous RCTs to calculate required information size (RIS). For continuous outcomes, we used the built-in empirical algorithm in the software to calculate RIS. When the cumulative Z curve entered the futility area or crossed the trial sequential monitoring boundary, a sufficient level of evidence may have been reached to confirm the results. If the Z curve didn’t cross any boundaries and RIS has not been reached, more trials would be required.

### 2.9 Certainty of evidence

Two authors independently assessed the certainty of evidence by using the Grading of Recommendations Assessment, Development, and Evaluation (GRADE) approach in GRADEpro GDT software^1^. Any disagreement would be considered by a third author to make final decision.

## 3 Results

### 3.1 Screening

**FIGURE 1:**
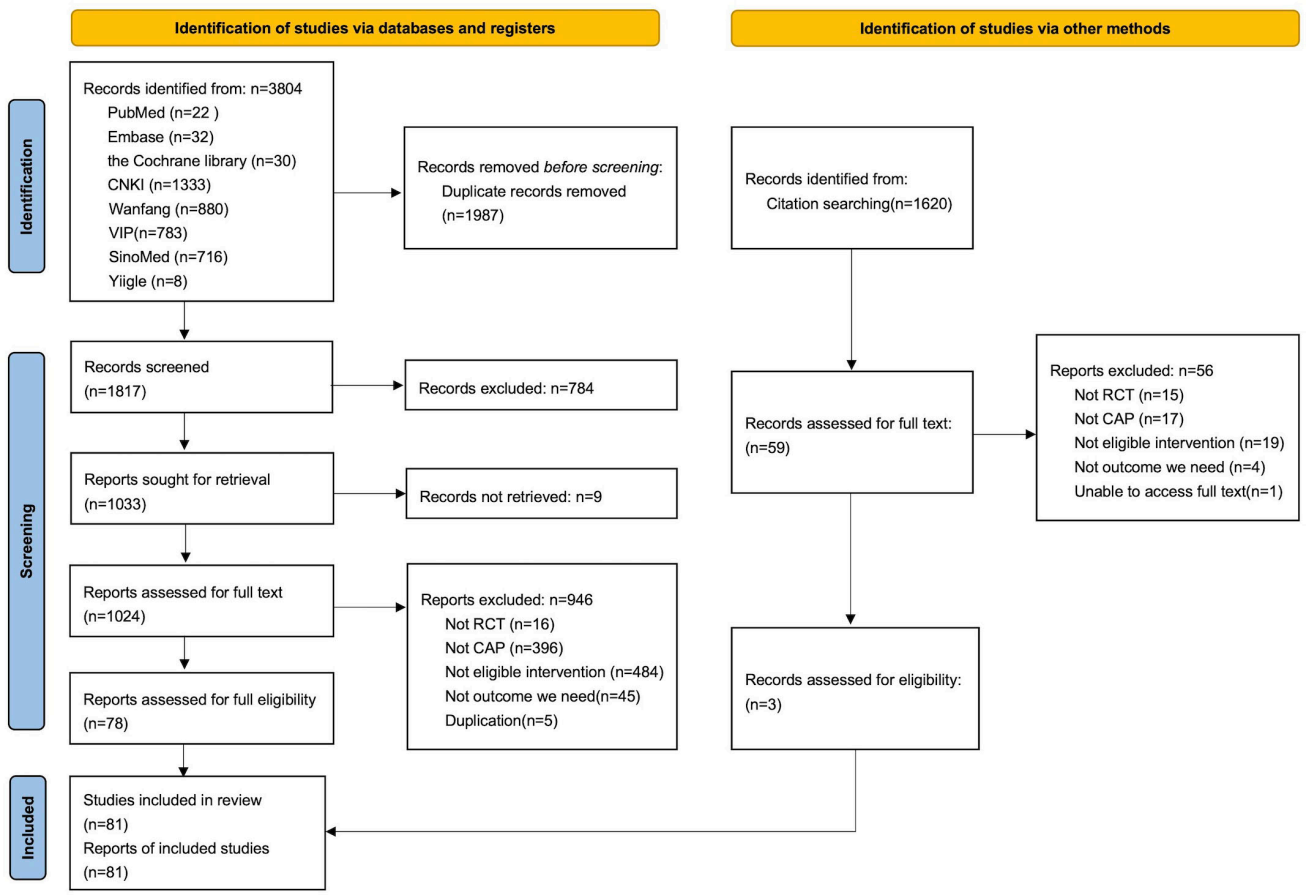
PRISMA flow chart of studies searching and screening.

3804 records were identified initially from the databases and 1987 duplicates were removed before screening. After reading the titles and abstracts, we excluded 784 studies according to the eligibility criteria. Nine studies were excluded because of the lack of full text, so only 1024 studies were downloaded. And 78 studies were included after reading the whole text. Meanwhile, we also retrieved the references of included studies and found three studies meeting the eligibility. Eventually, 81 studies (18–98) were included in this review. More details were presented in the PRISMA flow chart (Figure 1).

### 3.2 Characteristics of included studies

All RCTs included were conducted in China and published between 2006 and 2024, with 80 RCTs reported in Chinese and one in English. A total of 6682 participants aged from 2 months to 94 years old were enrolled. 15 RCTs focused on children, 10 RCTs only enrolled elders, 20 RCTs paid attention to adults, and the participants of the remaining 36 RCTs incorporated patients at different ages. In total, 20 studies reported severity of CAP, of which four studies recruited patients with severe CAP and the other 16 recruited patients with none-severe CAP. Only 10 studies reported the types of pathogen and participants in seven studies were infected with *Mycoplasma pneumonia*. The included studies covered a total of four comparison types, which were respectively, MXSG vs. placebo, MXSG vs. WM, MXSG plus WM vs. WM, and MXSG+WM1 vs. WM1+WM2.

In terms of intervention, only five studies used Standard MXSG (S-MXSG, the original prescription of MXSG), and the remaining used Modified MXSG (M-MXSG, the modified prescription of MXSG added with more Chinese herbal medicine based on S-MXSG). As for the composition, M-MXSG in 76 included studies were additionally supplemented with other kinds of Chinese herbal medicine. The additional Chinese herbal medicine mainly played the effects of clearing heat and detoxifying, relieving cough and reducing sputum, freeing lung and relieving asthma, thus assisting MXSG to play a more significant effect (Supplementary Table 1). Furthermore, more than 60% studies applied M-MXSG containing more than double the amount of Chinese herbal medicine than that in the original formulation. Almost half of the included studies took TCM syndrome differentiation and treatment based on the fixed prescription, according to the specific symptoms or TCM syndrome of each patient. The other studies gave the same fixed decoction to all participants. A total of 78 RCTs used antibiotics in WM group. And the kinds of antibiotics mainly incorporated macrolides (such as azithromycin), quinolones (such as levofloxacin and moxifloxacin), and cephalosporins (such as cefoperazone and ceftriaxone). For the report of primary outcomes, 31 studies evaluated the duration of symptoms and 33 studies recorded adverse events. As for secondary outcomes, 51 RCTs reported relevant laboratory indicators, 46 RCTs mentioned the improvement of chest radiographs, six RCTs evaluated lung function, seven recorded the hospitalization length, and only one reported mortality. The composition of Chinese herbal formulations could be seen in Supplementary Table 2. The details of the included studies were shown in Table 1.

**TABLE 1:** Characteristics of the included studies.

Study ID	Sample size	Gender (male/female)		Age (years old)		Duration of symptoms before treatments		Intervention		Course of treatment (d)	Outcome
		T	C	T	C	T	C	T	C		
**MXSG+WM vs. WM, 78 studies**											
Chen (18)	53	14/13	15/11	65.3 ± 2.7	66.1 ± 2.5	(7.1 ± 0.5) d	(7.3 ± 0.6) d	M- -MXSG+antibiotics+symptomatic treatment	Antibiotics+symptomatic treatment	30	➀➂
Cheng et al. (20)	58	15/14	16/13	72.37 ± 3.26	73.38 ± 3.15	(5.24 ± 1.27) d	(5.31 ± 1.42) d	M-MXSG+moxifloxacin	Moxifloxacin	14	➁➂➃
Cheng et al. (21)	70	18/17	16/19	59.68 ± 15.98	58.77 ± 15.28	NR	NR	M-MXSG+antibiotics	Antibiotics	10	➂➃
Cheng et al. (22)	80	23/17	17/23	51.63 ± 20.00	54.43 ± 20.68	(36.82 ± 18.06) h	(38.47 ± 19.73) h	N- -MXSG+antibiotics+symptomatic treatment	Antibiotics+symptomatic treatment	7	➂➄
Chu (23)	94	25/22	26/21	41.87 ± 6.76	42.29 ± 6.82	(7.15 ± 1.52) d	(7.38 ± 1.64) d	M-MXSG+cefuroxime sodium+symptomatic treatment	Cefuroxime sodium+symptomatic treatment	7	➂➃
Cui et al. (24)	160	52/28	48/32	52.82 ± 6.61	52.45 ± 6.58	(2.63 ± 1.73) m	(12.47 ± 1.71) m	M- -MXSG+antibiotics+symptomatic treatment	Antibiotics+symptomatic treatment	7	➁➂➃➄
Dai (25)	110	32/23	30/25	53.32 ± 10.21	52.89 ± 9.92	(7.45 ± 1.31) d	(7.49 ± 1.31) d	M-MXSG+symptomatic treatment	Symptomatic treatment	NR	➁➂➃
Deng (26)	88	24/20	25/19	61.6 ± 7.8	61.2 ± 7.5	(6.8 ± 2.1) d	(6.5 ± 2.2) d	N- -MXSG+antibiotics+symptomatic treatment	Antibiotics+symptomatic treatment	10	➂
Dong et al. (27)	106	25/28	29/24	52.14 ± 10.93	51.36 ± 11.85	(4.30 ± 0.82) d	(4.32 ± 0.84) d	M-MXSG+imipenem and cilastatin sodium+symptomatic treatment	Imipenem and cilastatin sodium+symptomatic treatment	7	➃
Du (28)	300	82/68	79/71	2.29 ± 0.53	2.32 ± 0.49	NR	NR	S-MXSG+azithromycin+symptomatic treatment	Azithromycin+symptomatic treatment	21	➂
Fang and Long (29)	98	21/28	23/26	5.0 ± 1.2	5.2 ± 1.4	(2.3 ± 0.4) d	(2.4 ± 0.4) d	M-MXSG+cefodizime sodium+symptomatic treatment	Cefodizime sodium+symptomatic treatment	14	➀
Fei (30)	60	15/15	14/16	75.2 ± 11.85	73.3 ± 12.44	NR	NR	M-MXSG+antibiotics+symptomatic treatment	Antibiotics+symptomatic treatment	7	➂
Gao (31)	94	23/24	25/22	7.14 ± 1.49	6.93 ± 1.65	(6.98 ± 1.02) d	(7.34 ± 0.96) d	M-MXSG+azithromycin	Azithromycin	21	➃
Guo (32)	87	25/19	23/20	61.01 ± 8.34	61.64 ± 7.20	(3.46 ± 0.59) d	(3.41 ± 0.64) d	M- -MXSG+antibiotics+symptomatic treatment	Antibiotics+symptomatic treatment	12	➀➁➂
He (33)	60	20/19	11/9	67 ∼ 89	67 ∼ 90	NR	NR	M- -MXSG+antibiotics+symptomatic treatment	Antibiotics+symptomatic treatment	5 ∼ 7	➂
Hu (34)	60	30	30	52.63 ± 5.41	53.76 ± 5.44	NR	NR	M- -MXSG+antibiotics+symptomatic treatment	Antibiotics+symptomatic treatment	5	➂
Hu (35)	130	66	64	3 ∼ 7	3 ∼ 7	NR	NR	S-MXSG+azithromycin+symptomatic treatment	Azithromycin+symptomatic treatment	12	➂➃
Huo et al. (36)	78	NR	NR	4.77 ± 0.52	4.77 ± 0.52	NR	NR	M-MXSG+cefodizime sodium+symptomatic treatment	Cefodizime sodium+symptomatic treatment	14	➀➁➂➃
Jin (37)	84	9/31	14/26	49.73 ± 2.19	50.40 ± 2.18	NR	NR	M-MXSG+levofloxacin+symptomatic treatment	Levofloxacin+symptomatic treatment	10 (±3)	➀➁➂➃
Kong et al. (38)	120	34/26	31/29	54.38 ± 9.97	55.91 ± 9.26	(12.81 ± 3.73) d	(12.06 ± 3.84) d	S- -MXSG+antibiotics+symptomatic treatment	Antibiotics+symptomatic treatment	21	➁➄
Li (39)	79	20/19	23/17	18 ∼ 65	20 ∼ 64	(4.00 ± 2.12) d	(4.00 ± 2.02) d	M-MXSG+antibiotics	Antibiotics	10 ∼ 14	➀➁➃
Li (40)	45	14/8	13/10	53.7 ± 2.8	51.2 ± 3.7	(5.7 ± 1.1) d	(6.1 ± 0.9) d	M-MXSG+moxifloxacin+ symptomatic treatment	Moxifloxacin+symptomatic treatment	10	➀➁➂➃
Li et al. (41)	88	25/19	23/21	4.8 ± 0.7	4.9 ± 0.7	(4.5 ± 0.8) d	(4.6 ± 0.7) d	M-MXSG+azithromycin	Azithromycin	7	➁➂➃
Li and Zhang (42)	100	27/24	27/22	67.9 ± 12.7	65.8 ± 11.9	(7.8 ± 4.2) d	(8.1 ± 3.9) d	M- -MXSG+antibiotics+symptomatic treatment	Antibiotics+symptomatic treatment	10	➀➁➂
Li et al. (43)	84	24/18	23/19	37.4 ± 10.2	37.2 ± 10.1	(6.5 ± 1.3) d	(6.4 ± 1.5) d	M-MXSG+cefoxitin sodium+symptomatic treatment	Cefoxitin sodium+symptomatic treatment	7	➀➂
Li and Huang (44)	74	18/19	17/20	6.25 ± 1.28	6.12 ± 1.23	(17.62 ± 3.74) d	(17.28 ± 3.44) d	M-MXSG+azithromycin+ symptomatic treatment	Azithromycin+symptomatic treatment	12	➀➁➃
Liu (45)	68	18/16	19/15	54.38 ± 4.83	54.44 ± 4.91	(16.35 ± 4.92) h	(16.28 ± 4.87) h	M-MXSG+cefoperazone sodium and sulbactam sodium+azithromycin	Cefoperazone sodium and sulbactam sodium+azithromycin	14	➀➃➅
Liu (46)	60	11/19	13/17	38.43 ± 5.28	35.24 ± 6.23	(3.54 ± 1.02) d	(3.54 ± 1.03) d	M-MXSG+levofloxacin	Levofloxacin	10	➃
Liu et al. (47)	80	24/16	23/17	53.29 ± 1.23	52.78 ± 1.04	(65.23 ± 9.27) h	(64.83 ± 9.16) h	M-MXSG+antibiotics+symptomatic treatment	Antibiotics+symptomatic treatment	7 ∼ 14	➀➄
Liu et al. (51)	62	17/14	15/16	55.59 ± 4.27	56.08 ± 4.19	NR	NR	M-MXSG+antibiotics+symptomatic treatment	Antibiotics+symptomatic treatment	14	➁➂
Liu et al., (48)	50	25	25	70.5 ± 4.7	70.5 ± 4.7	NR	NR	M-MXSG+moxifloxacin	Moxifloxacin	7	➀➂➃
Liu (49)	60	19/11	16/14	37.43 ± 13.70	39.47 ± 16.49	(4.06 ± 1.26) d	(3.86 ± 1.09) d	M-MXSG+antibiotics+symptomatic treatment	Antibiotics+symptomatic treatment	7	➁➃➅
Huo et al. (36)	78	NR	NR	4.77 ± 0.52	4.77 ± 0.52	NR	NR	M-MXSG+cefodizime sodium+symptomatic treatment	Cefodizime sodium+symptomatic treatment	14	➀➁➂➃
Liu et al. (50)	60	18/12	17/13	38.13 ± 13.70	39.27 ± 16.19	(4.06 ± 1.26) d	(3.86 ± 1.09) d	M-MXSG+antibiotics+ symptomatic treatment	antibiotics+ symptomatic treatment	7	➂
Lu (52)	74	NR	NR	13∼78	13∼78	NR	NR	M-MXSG+ulinastain+symptomatic treatment	Ulinastain+symptomatic treatment	7	➁
Ma (53)	106	31/22	30/23	55.01 ± 7.70	54.60 ± 7.52	(3.11 ± 0.83) d	(3.02 ± 0.71) d	M-MXSG+cefuroxime sodium+symptomatic treatment	Cefuroxime sodium+symptomatic treatment	14	➀➂➃➅
Ma (54)	60	15/14	16/13	51.97 ± 15.69	48.56 ± 17.09	NR	NR	M-MXSG+levofloxacin+symptomatic treatment	Levofloxacin+symptomatic treatment	7	➁➃
Ma and Chen (55)	62	18/13	16/15	35 ∼ 84	28 ∼ 86	2∼12 d	2∼11 d	M-MXSG+antibiotics+symptomatic treatment	Antibiotics+symptomatic treatment	15	➀➃
Ma (56)	100	26/24	31/19	2.711 ± 2.259	1.700 ± 1.801	(7.600 ± 4.899) d	(8.020 ± 4.565) d	M-MXSG+cefuroxime sodium+symptomatic treatment	Cefuroxime sodium+symptomatic treatment	7	➁➂
Meng et al. (57)	80	22/18	24/16	62.50 ± 8.30	64.20 ± 7.50	(65.70 ± 9.50) h	(63.80 ± 8.60) h	M-MXSG+cefoperazone sodium and sulbactam sodium+symptomatic treatment	Cefoperazone sodium and sulbactam sodium+symptomatic treatment	7 ∼ 14	➀➂
Mo et al. (58)	53	15/12	14/12	55.62 ± 4.50	55.24 ± 4.36	NR	NR	M-MXSG+imipenem and cilastatin sodium+ulinastain+symptomatic treatment	Imipenem and cilastatin sodium+ulinastain+symptomatic treatment	14	➂➃
Mo (59)	50	14/11	12/13	47.28 ± 13.21	46.44 ± 9.59	NR	NR	M-MXSG+moxifloxacin+ambroxol hydrochloride	Moxifloxacin+ambroxol hydrochloride	7	➁➂➃
Ni (60)	80	22/18	21/19	51.34 ± 3.23	51.36 ± 3.21	(4.23 ± 0.34) d	(4.21 ± 0.32) d	M-MXSG+cefotaxime sodium	Cefotaxime sodium	7	➀➁➄
Ning et al. (61)	86	27/16	26/17	58.15 ± 6.04	58.09 ± 6.01	(4.22 ± 1.03) d	(4.29 ± 1.08) d	M-MXSG+cefuroxime sodium	Cefuroxime sodium	7	➀➂➃
Shen et al. (62)	80	40	40	64.37 ± 11.35	64.71 ± 11.45	(7.23 ± 4.28) d	(7.14 ± 4.33) d	M-MXSG+antibiotics+N-symptomatic treatment	Antibiotics+symptomatic treatment	10	➁➂
Shen and Zhou (63)	50	13/12	14/11	35 ∼ 70	28 ∼ 72	2∼7 d	2∼8 d	M-MXSG+antibiotics	Antibiotics	10	➂➃
Shi (64)	100	27/23	26/24	44.56 ± 6.35	47.36 ± 7.65	(4.51 ± 0.63) d	(4.62 ± 0.53) d	M-MXSG+levofloxacin	Levofloxacin	10	➀
Song (65)	100	28/22	26/24	4.85 ± 1.12	4.76 ± 1.33	(3.13 ± 1.03) d	(3.08 ± 1.01) d	M-MXSG+cefuroxime sodium or azithromycin+symptomatic treatment	Cefuroxime sodium or azithromycin+symptomatic treatment	7	➀➁➂➃
Su and Yang (66)	60	20/10	16/14	62.7 ± 7.9	63.5 ± 8.3	(65.3 ± 9.8) h	(64.6 ± 9.0) h	M-MXSG+cefoperazone sodium and sulbactam sodium	Cefoperazone sodium and sulbactam sodium	7∼14	➃
Su et al. (67)	80	23/17	25/15	40 ± 5.6	43 ± 6.8	(3 ± 1.2) d	(3 ± 1.1) d	S-MXSG+cefuroxime sodium	Cefuroxime sodium+symptomatic treatment	14	➃
Sun (68)	64	17/15	18/14	67.15 ± 7.95	64.65 ± 8.25	(7.15 ± 4.34) d	(7.32 ± 4.28) d	M- -MXSG+cefazolin sodium and levofloxacin+symptomatic treatment	Cefazolin sodium and levofloxacin+symptomatic treatment	10	➂
Sun et al. (69)	108	28/26	29/25	9.6 ± 8.4	9.2 ± 8.8	(1.6 ± 0.4) d	(1.4 ± 0.5) d	M-MXSG+cefoperazone sodium and sulbactam sodium+symptomatic treatment	Cefoperazone sodium and sulbactam sodium+symptomatic treatment	7	➀➂➃➅
Sun (70)	130	38/27	35/30	67. 28 ± 5. 15	67. 28 ± 5. 15	NR	NR	M-MXSG+cefuroxime sodium+symptomatic treatment	Cefuroxime sodium+symptomatic treatment	20	➀➁➂➃
Tang and Chen (71)	90	25/20	24/21	69.60 ± 6.10	71.16 ± 6.51	NR	NR	M-MXSG+cefoperazone sodium and sulbactam sodium+symptomatic treatment	Cefoperazone sodium and sulbactam sodium+symptomatic treatment	10	➁➂➃
Tian and Hu (73)	76	23/15	25/13	42.6 ± 7.5	41.8 ± 7.9	(2.3 ± 1.1) d	(2.0 ± 1.2) d	M-MXSG+levofloxacin+symptomatic treatment	Levofloxacin+symptomatic treatment	10	➃
Wang et al. (74)	60	30	30	NR	NR	NR	NR	M-MXSG+antibiotics+symptomatic treatment	Antibiotics+symptomatic treatment	10	➂
Wang and Zhou (75)	50	14/11	15/10	50	41	3.5d	4d	M-MXSG+azithromycin+ cefoperazone sodium and sulbactam sodium	Azithromycin+cefoperazone sodium and sulbactam sodium	10	➃
Wang et al. (76)	60	13/17	14/16	5.3 ± 1.4	5.2 ± 1.3	(23.8 ± 3.2) d	(23.5 ± 2.8) d	M-MXSG+azithromycin+ symptomatic treatment	Azithromycin+symptomatic treatment	14	➀
Wang (77)	56	20/8	18/10	55.18 ± 4.7	54.23 ± 5.6	NR	NR	M-MXSG+moxifloxacin	Moxifloxacin	14	➃
Wang (78)	106	33/25	30/18	3.63 ± 1.54	3.87 ± 1.83	4.23 d	4.52 d	M-MXSG+cefoperazone sodium and sulbactam sodium+symptomatic treatment	Cefoperazone sodium and sulbactam sodium+symptomatic treatment	7	➀➅
Wang (79)	60	17/13	16/14	49.69 ± 4.38	49.75 ± 4.42	NR	NR	M- -MXSG+antibiotics+symptomatic treatment	Antibiotics+symptomatic treatment	7∼10	➁➂
Wu et al. (80)	123	33/30	32/28	7.6 ± 1.7	7.3 ± 1.8	(4.3 ± 1.4) d	(4.2 ± 1.6) d	M-MXSG+azithromycin	Azithromycin	21	➀➃
Wu et al. (81)	82	17/23	20/22	59.3 ± 14.6	61.2 ± 13.7	(9.6 ± 2.4) d	(10.9 ± 2.4) d	N- -MXSG+antibiotics+symptomatic treatment	Antibiotics+symptomatic treatment	10	➀➂➅
Xiao (82)	80	25/12	23/15	50.135 ± 13.039	49.711 ± 13.160	NR	NR	M-MXSG+moxifloxacin+ symptomatic treatment	Moxifloxacin+symptomatic treatment	10	➁➂➃
Xie (83)	60	23/7	24/6	2.814 ± 1.503	2.883 ± 1.0228	NR	NR	M-MXSG+ceftriaxone sodium+symptomatic treatment	Cefuroxime sodium+symptomatic treatment	7	➀➅
Xie et al. (84)	96	29/19	26/22	73.47 ± 4.38	71.23 ± 6.16	(3.78 ± 1.60) d	(3.60 ± 2.07) d	M- -MXSG+antibiotics+symptomatic treatment	Moxifloxacin+symptomatic treatment	14	➂
Xin (85)	120	51	69	46.76 ± 13.72	46.76 ± 13.72	(4.79 ± 2.36) d	(4.79 ± 2.36) d	M-MXSG+latamoxef sodium+symptomatic treatment	Latamoxef sodium+symptomatic treatment	7	➁➂
Xu (86)	60	19/11	20/10	32 ∼ 78	33 ∼ 79	NR	NR	M-MXSG+antibiotics+symptomatic treatment	Antibiotics+symptomatic treatment	14	➂➃➆
Xu (87)	60	18/12	17/13	43.25 ± 4.35	44.10 ± 3.87	NR	NR	M-MXSG+symptomatic treatment	Symptomatic treatment	5	➁➂
Yang (88)	88	NR	NR	NR	NR	3.2 d	3.2 d	M- -MXSG+antibiotics+symptomatic treatment	Antibiotics+symptomatic treatment	10	➃
Yang (89)	60	18/12	17/13	45.5 ± 3.8	45.8 ± 3.9	(4.32 ± 1.57) d	(4.29 ± 1.45) d	M-MXSG+cefoperazone sodium and sulbactam sodium+symptomatic treatment	Cefoperazone sodium and sulbactam sodium+symptomatic treatment	14	➀➃
Yang (90)	62	21/10	22/9	65 ∼ 79	65 ∼ 80	5–10 d	4–11 d	M-MXSG+cefoperazone sodium and sulbactam sodium+levofloxacin	Cefoperazone sodium and sulbactam sodium+levofloxacin	7	➂
Yuan (91)	60	16/14	17/13	66.43 ± 8.23	64.73 ± 6.97	(3.87 ± 1.66) d	(3.37 ± 1.59) d	N- -MXSG+levofloxacin+symptomatic treatment	Levofloxacin+symptomatic treatment	10	➁➂
Zhang (92)	80	26/14	25/15	52.78 ± 3.52	52.78 ± 3.52	(6.31 ± 2.76) d	(6.23 ± 1.89) d	M- -MXSG+antibiotics+symptomatic treatment	Antibiotics+symptomatic treatment	10	➁➃
Zhang (93)	120	NR	NR	5.84 ± 1.47	5.67 ± 1.62	(7.65 ± 3.14) d	(5.81 ± 1.76) d	M-MXSG+azithromycin+ symptomatic treatment	Azithromycin+symptomatic treatment	14	➀➁
Zhou et al. (95)	80	23/17	25/15	49.60 ± 6.10	50.16 ± 6.51	(10.95 ± 1.60) d	(10.80 ± 1.79) d	M-MXSG+cefuroxime sodium+levofloxacin+symptomatic treatment	Cefuroxime sodium+ levofloxacin+symptomatic treatment	10	➁➂➃
Zhou (96)	90	26/19	27/18	66.08 ± 6.13	66.32 ± 6.74	NR	NR	M-MXSG+cefuroxime sodium	Cefuroxime sodium	14	➀➃
Zhu et al. (97)	102	34/17	32/19	60.12 ± 19.97	60.12 ± 18.32	(9.93 ± 2.02) d	(9.32 ± 1.77) d	M-MXSG+symptomatic treatment	Symptomatic treatment	5	➁➂
Zou et al. (98)	60	18/12	16/14	42.1 ± 14.2	41.3 ± 15.1	NR	NR	M- -MXSG+antibiotics+symptomatic treatment	Antibiotics+symptomatic treatment	10	➂
**MXSG vs. WM, 1 study**											
Chen et al. (19)	40	8/12	10/10	61.95 ± 12.37	60.15 ± 9.21	(3.20 ± 1.01) d	(3.30 ± 1.17) d	M-MXSG+symptomatic treatment	Levofloxacin+symptomatic treatment	10	➂➃
**MXSG+WM 1 vs. WM 1+WM 2, 1 study**											
Tian et al. (72)	98	22/27	23/26	49.6 ± 2.35	49.8 ± 2.36	(6.12 ± 3.1) d	(6.14 ± 3.13) d	M-MXSG+moxifloxacin	Moxifloxacin+symptomatic treatment	14	➁➂➃
**MXSG vs. placebo, 1 study**											
Zheng et al. (94)	80	16/20	17/18	4.28 ± 0.96	4.47 ± 1.09	(37.00 ± 14.63) h	(39.26 ± 14.16) h	S-MXSG	Placebo	10	➀➁➃

T, treatment group; C, control group; m, month; d, day; h, hour; NR, not report; S-MXSG, Standard-Maxing Shigan Decoction; M-MXSG, Modified-Maxing Shigan Decoction. ➀ Resolution time of clinical symptoms (fever, cough, phlegm, pulmonary crepitation, dyspnea, chest pain). ➁ Adverse events. ➂ Relevant laboratory indicators (WBC, CRP, PCT). ➃ Improvement of chest radiograph (improvement rate of chest radiograph, absorption time of lung inflammation). ➄ Lung function (FVC, FEV1, PEF). ➅ Length of hospitalization. ➆ All-cause mortality.

### 3.3 Risk of bias assessment

In the aspect of randomization, 42 RCTs used random number tables and one RCT used software to generate random sequences, but the other 38 RCTs failed to report random sequence generation. Besides, none of studies reported the information of allocation concealment. However, in all the included studies, there was no significant baseline difference among intervention groups. Therefore, all studies were determined to be of uncertain risk.

In terms of deviations from intended interventions, only one study conducted blind method by applying MXSG placebo in control group, but the other 80 studies failed to blinded participants. Furthermore, a total of three studies didn’t use appropriate analysis to estimate the effect of assignment to intervention. After comprehensive assessment, two studies were rated as high risk, while the others were considered to have an uncertain risk.

As for the bias of outcome measurement, only two studies were judged as low risk, among which one study blinded the outcome assessors, and the other used objective laboratory outcomes. The remaining 79 studies didn’t mention the blinding of outcome assessment so that were rated as high risk.

For incomplete outcome data, 77 RCTs had no or only a few missing data and were assessed as low risk. But the drop-out rates in three RCTs were over 5% and the other one RCT didn’t report the number of patients after treatment, which resulted in high risk for this item.

As to the selective reporting, none of studies mentioned registration protocol. Of which, two studies didn’t sufficiently report the expected outcome indicators, so were considered as unclear risk. In the remaining 79 studies, the outcomes in Section “3 Results” were the same as that in Section “2 Materials and methods,” resulting in low risk of bias.

**FIGURE 2:**
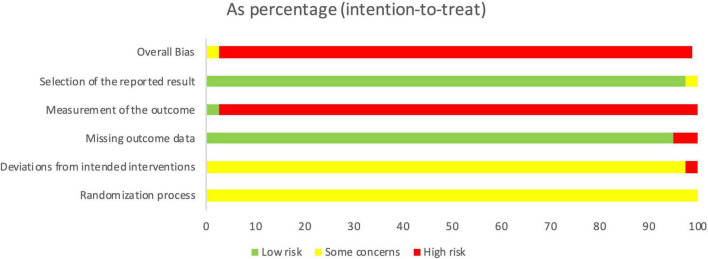
Risk of bias assessment for eligible studies.

In conclusion, only two RCTs were judged to have moderate risk of bias and the other RCTs were considered as high risk of bias (Figure 2).

### 3.4 Primary outcomes

#### 3.4.1 Resolution time of fever

##### 3.4.1.1 MXSG+WM versus WM

**FIGURE 3:**
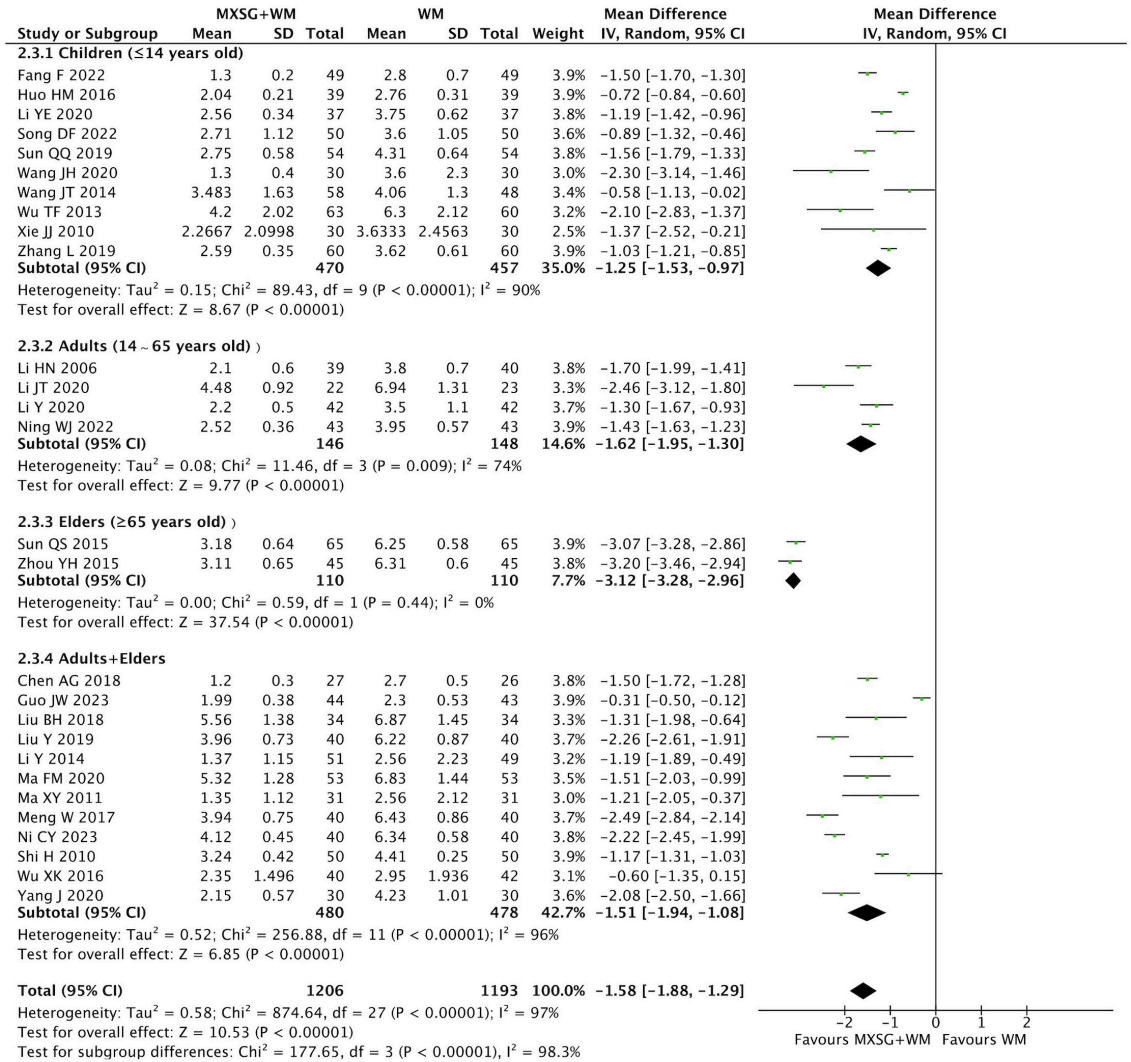
Forest plot of resolution time of fever by age. Comparison: MXSG plus WM vs. WM. MXSG, Maxing Shigan Decoction; WM, western medicine.

A total of 28 RCTs (2399 participants) took this type of comparison. Compared with WM alone, MXSG plus WM significantly reduced fever duration (MD = −1.58 days, 95% CI: −1.88 to −1.29, *p* < 0.00001; I^2^ = 97%) (Figure 3).

For subgroup analysis, heterogeneity in all subgroups decreased when classified by age, but high heterogeneity still existed in children group and adults plus elders group. Furthermore, MXSG plus WM may be more effective for elders on fever resolution (MD = −3.12 days, 95% CI: −3.28 to −2.96, *p* < 0.00001; I^2^ = 0%). However, neither the application of syndrome differentiation nor the flavored quantity of Chinese herbal medicine accounted for the overall high heterogeneity. And subgroup analysis based on the severity of pneumonia was failed to performed as most studies didn’t report this information (Supplementary Table 3). Although sensitivity analysis demonstrated the robustness of the findings, it could not fully explain the sources of heterogeneity. Through a comprehensive analysis of the included studies, we found that the high heterogeneity might stem from the clinical diversity including inconsistent baseline disease severity, significant differences in baseline disease duration and variations in western medical treatment plans. Additionally, most studies did not clearly define the measurement standards and methods for the resolution time of fever, which might lead to methodological heterogeneity.

##### 3.4.1.2 MXSG versus placebo

Only one study (80 participants) compared MXSG with placebo using median time to record the duration of fever. The median time to fever resolution in MXSG group was 0.5 (. to.) days, which was shorter than 1.0 (0.5–1.5) days in control group (*p* < 0.05).

#### 3.4.2 Resolution time of cough

**FIGURE 4:**
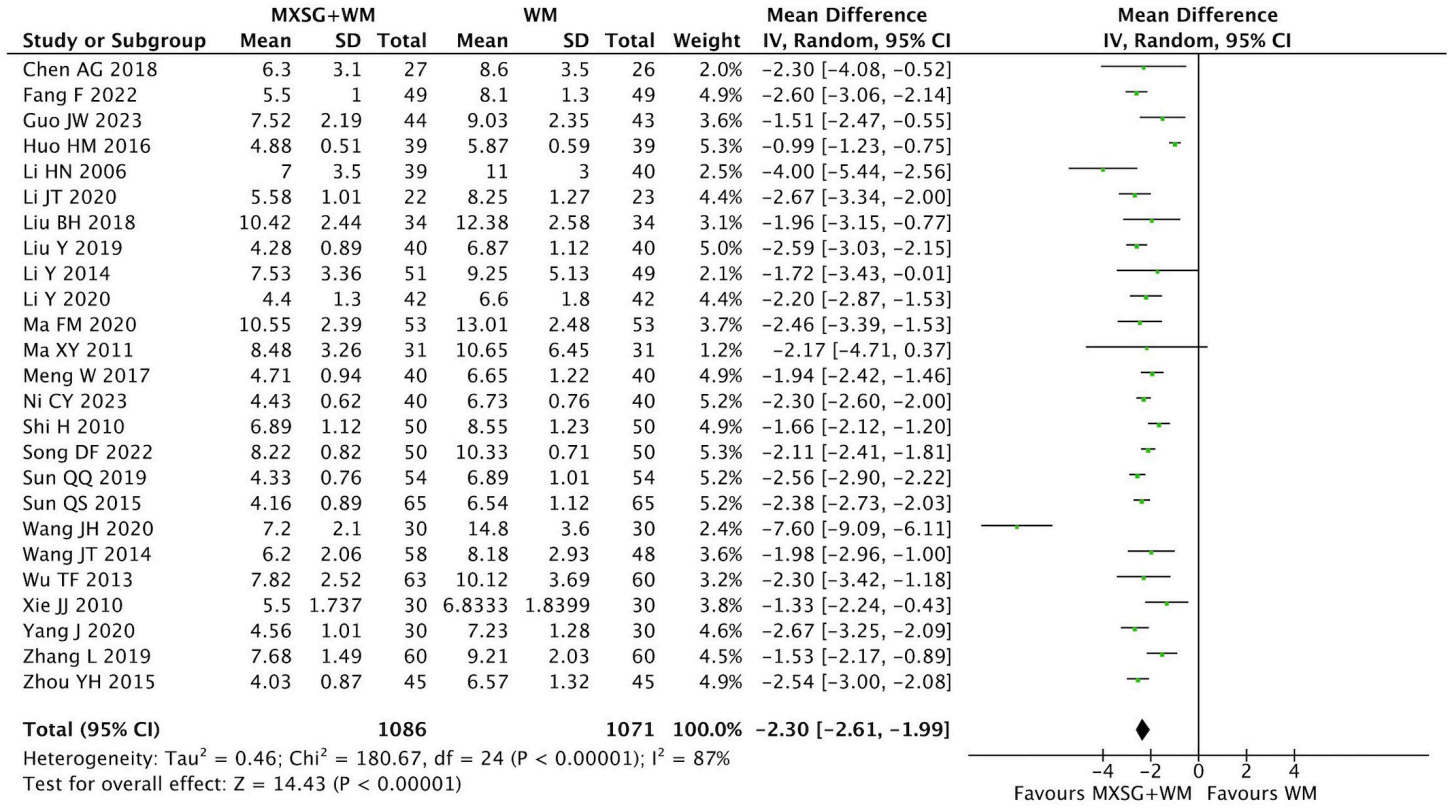
Forest plot of resolution time of cough. Comparison: MXSG plus WM vs. WM. MXSG, Maxing Shigan Decoction; WM, western medicine.

By comparing MXSG plus WM with WM, 25 RCTs (2157 participants) reported the resolution time of cough. The pooled data showed that the duration of cough in MXSG plus WM group was shorter than that in WM group (MD = −2.30 days, 95% CI: −2.61 to −1.99, *p* < 0.00001; I^2^ = 87%) (Figure 4).

When conducting subgroup analyses based on age, flavored quantity of Chinese medicine, and the application of syndrome differentiation, the heterogeneity within some subgroups did not decrease (Supplementary Table 3). To further explore heterogeneity sources, sensitivity analysis revealed that the exclusion of two trials by Huo HM (36) and Wang JH (76) reduced overall heterogeneity to I^2^ = 42%, indicating these studies were key contributors of heterogeneity. Distinctive features of these trials included: First, the included patients were all children with pneumonia aged 4–5 years old; second, the types of pneumonia were special, including bronchopneumonia and *Mycoplasma pneumonia*; third, the baseline symptom duration of cough was relatively long, with an average of 23.8 days. Additionally, unreported assessment methods for cough outcomes in most studies may introduce methodological measurement bias.

#### 3.4.3 Resolution time of phlegm

**FIGURE 5:**
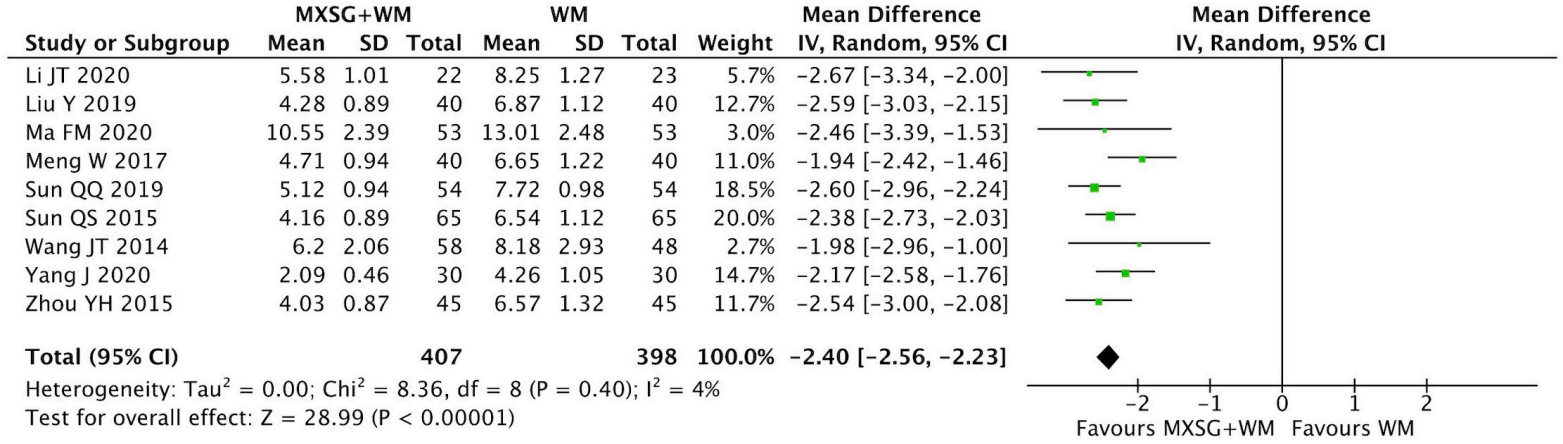
Forest plot of resolution time of phlegm. Comparison: MXSG plus WM vs. WM. MXSG, Maxing Shigan Decoction; WM, western medicine.

Nine RCTs including 805 participants evaluated this outcome. When compared with WM group, the resolution time of phlegm in MXSG plus WM group was significantly shorter. (MD = −2.40 days, 95% CI: −2.56 to −2.23, *p* < 0.00001; I^2^ = 4%) (Figure 5).

#### 3.4.4 Resolution time of dyspnea

**FIGURE 6:**
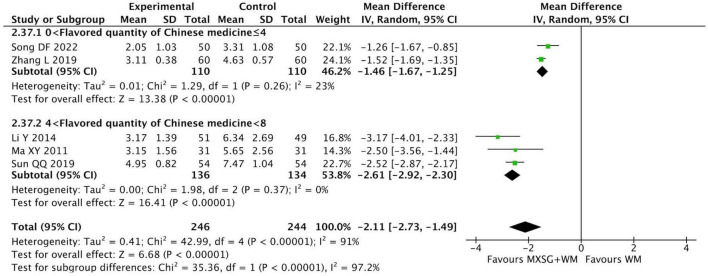
Forest plot of resolution time of dyspnea by flavored quantity of Chinese medicine. Comparison: MXSG plus WM vs. WM. MXSG, Maxing Shigan Decoction; WM, western medicine.

Five studies involving 490 participants compared MXSG plus WM with WM alone on this outcome. The pooled data indicated that the resolution time of dyspnea in MXSG plus WM group reduced more than that in WM group (MD = −2.11 days, 95% CI: −2.73 to −1.49, *p* < 0.00001; I^2^ = 91%) (Figure 6).

The result of subgroup analysis revealed that MXSG plus WM may be more effective in relieving dyspnea when the number of additional Chinese medicine was in the range of four to eight (MD = −2.61 days, 95% CI: −2.92 to −2.30, *p* < 0.00001; I^2^ = 0%). And the heterogeneity in each group had a significant reduction (Supplementary Table 3).

#### 3.4.5 Resolution time of chest pain

None of studies reported this outcome.

#### 3.4.6 Resolution time of pulmonary crepitation

**FIGURE 7:**
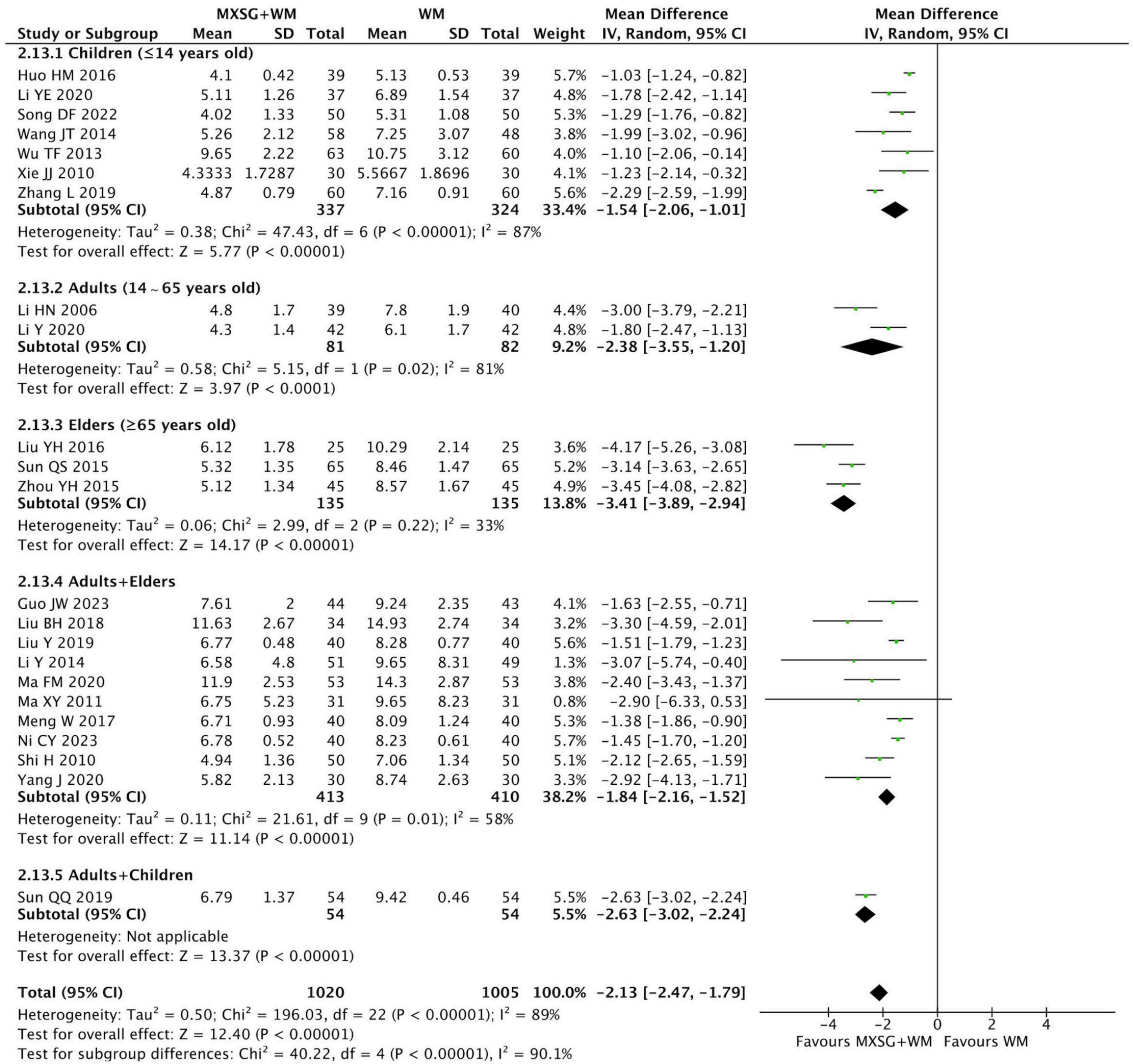
Forest plot of resolution time of pulmonary crepitation by age. Comparison: MXSG plus WM vs. WM. MXSG, Maxing Shigan Decoction; WM, western medicine.

23 studies (2025 participants) reporting the resolution time of pulmonary crepitation were pooled in a meta-analysis. The result demonstrated that the pulmonary crepitation in MXSG plus WM group disappeared faster than that in WM group (MD = −2.13 days, 95% CI: −2.47 to −1.79, *p* < 0.00001; I^2^ = 89%) (Figure 7).

Meanwhile, we observed that age may be the source of heterogeneity for this outcome. The data revealed that MXSG plus WM may reduce the duration of pulmonary crepitation more effectively on elders with CAP (MD = −3.41 days, 95% CI: −3.89 to −2.94, *p* < 0.00001; I^2^ = 33%) (Supplementary Table 3).

#### 3.4.7 Adverse events

**FIGURE 8:**
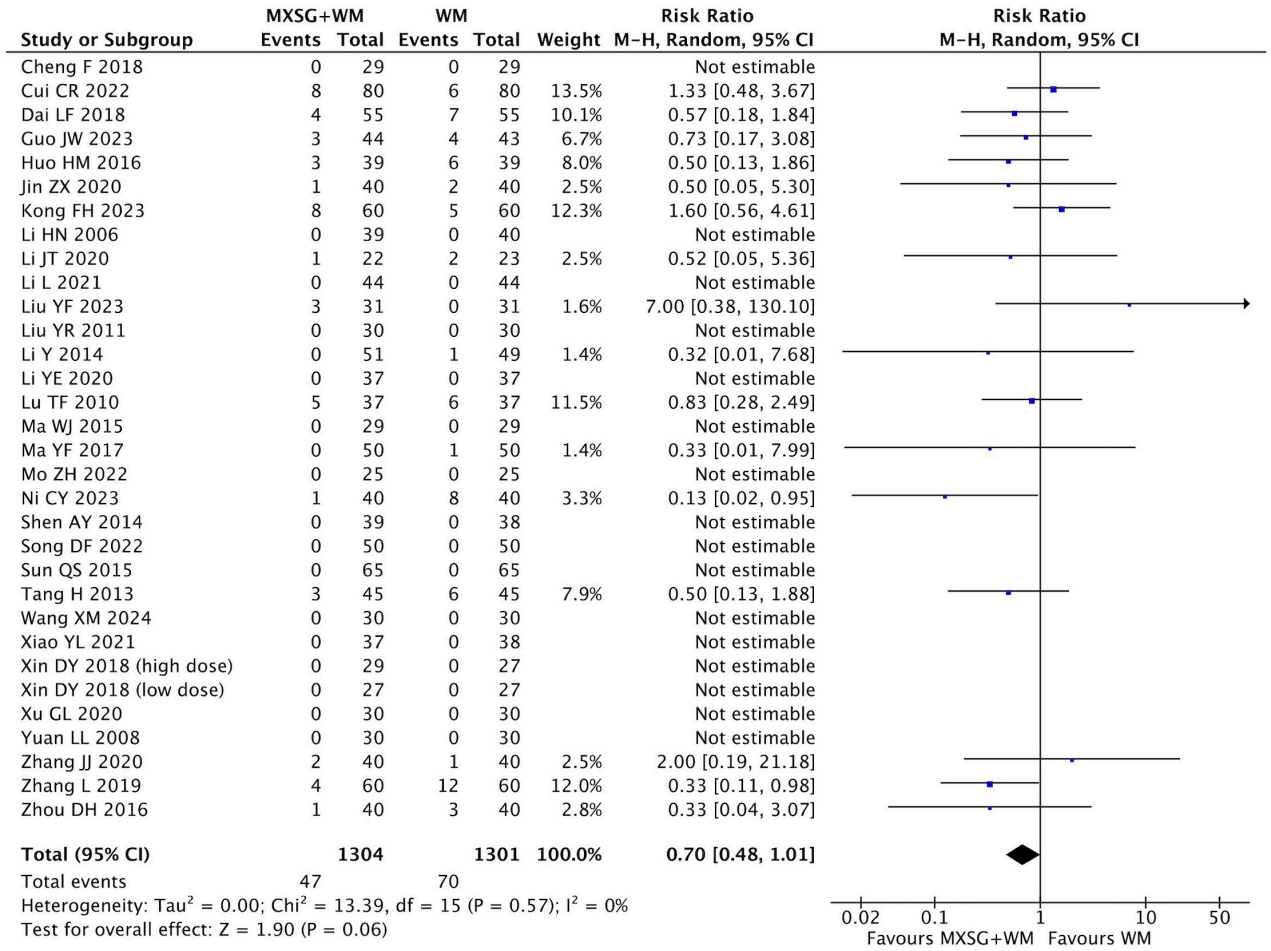
Forest plot of the incidence of adverse events. Comparison: MXSG plus WM vs. WM. MXSG, Maxing Shigan Decoction; WM, western medicine.

In total, 33 studies reported adverse events including nausea, inappetence, emesis, diarrhea, rash, dizziness, and other mild symptoms. Among these studies, 17 RCTs declared that no adverse events were found in both groups and the remaining 16 RCTs recorded the specific adverse reactions in detail (Table 2). The incidence of adverse reactions was 3.60% in MXSG plus WM group and 5.38% in WM group. (RR = 0.70, 95% CI: 0.48 to 1.01, *p* = 0.06; I^2^ = 0%) (Figure 8 and Supplementary Table 3).

**TABLE 2:** Adverse events of MXSG for patients with CAP in the included studies.

	Treatment group		Control group	
Study ID	Number of adverse events	Specific adverse event	Number of adverse events	Specific adverse event
**MXSG+WM vs. WM**				
Cui et al. (24)	8	4 for nausea; 1 for headache; 2 for sweating; 1 for dizzy	6	3 for nausea; 1 for headache; 1 for sweating; 1 for dizzy
Cheng et al. (20)	0	NR	0	NR
Dai (25)	4	3 for inappetence; 1 for diarrhea	7	2 for nausea and emesis; 3 for inappetence; 1 for mild increase of AST; 1 for mild increase of ALT
Guo (32)	3	3 for nausea, emesis and diarrhea	4	2 for dizziness and headache; 1 for nausea, emesis and diarrhea; 1 for lethargy
Huo et al. (36)	3	1 for mild laryngeal irritation; 1 for whiny; 1 for rash	6	2 for hoarseness; 2 for mild increase of ALT; 1 for mild laryngeal irritation; 1 for pain of mouth and tongue
Jin (37)	1	1 for loose stool	2	1 for dizzy and hyperactive; 1 for nausea, stomach discomfort and abdominal distension
Kong et al. (38)	8	2 for nausea and emesis; 2 for abdominal pain and diarrhea; 2 for inappetence; 2 for rash	5	2 for nausea and emesis; 1 abdominal pain; 1 for inappetence; 1 for rash
Li (39)	0	NR	0	NR
Li (40)	1	1 for dizzy	2	1 for dizzy; 1 for nausea
Li et al. (41)	0	NR	0	NR
Li and Zhang (42)	0	NR	1	1 for increased frequency of bowel movement
Li et al. (43)	0	NR	0	NR
Liu (51)	3	NR	0	NR
Liu (49)	0	NR	0	NR
Lu (52)	5	2 for diarrhea; 1 for decrease of WBC; 2 for rash	6	2 for decrease of WBC; 1 for diarrhea; 3 for rash
Ma (54)	0	NR	0	NR
Ma (56)	0	NR	1	1 for considered allergic to cefuroxime
Mo (59)	0	NR	0	NR
Ni (60)	0	NR	0	NR
Shen and Zhou (63)	0	NR	0	NR
Song (65)	0	NR	0	NR
Sun (70)	0	NR	0	NR
Tang and Chen (71)	3	1 for diarrhea; 2 for inappetence	6	4 for inappetence and nausea; 2 for mild increase of ALT and AST
Wang (79)	1	1 for nausea and emesis	8	3 for abdominal discomfort; 3 for nausea and emesis; 2 for minor rash
Xiao (82)	0	NR	0	NR
Xin (85)	0	NR	0	NR
Xu (87)	0	NR	0	NR
Yuan (91)	0	NR	0	NR
Zhang (92)	2	2 for increase of BUN	1	1 for increase of BUN
Zhang (93)	4	2 for mild nausea and emesis; 1 for abdominal pain; 1 for rash	12	4 for mild nausea and emesis; 3 for mild diarrhea; 3 for mild abdominal pain; 2 for rash
Zhou et al. (95)	1	1 for nausea and inappetence	3	3 for nausea and inappetence
**MXSG+WM 1 vs. WM 1+WM 2**				
Tian et al. (72)	0	NR	0	NR
**MXSG vs. placebo**				
Zheng et al. (94)	0	NR	0	NR

T, treatment group; C, control group; NR, not report; AST, glutamic oxaloacetic transaminase; ALT, glutamic pyruvic transaminase; WBC, white blood cell; BUN, blood urea nitrogen.

### 3.5 Secondary outcomes

#### 3.5.1 C-reactive protein (CRP)

##### 3.5.1.1 MXSG+WM versus WM

38 RCTs involving 3293 participants compared the level of CRP between MXSG plus WM and WM alone. There was high heterogeneity (I^2^ = 96%) among the studies. And all of the planned subgroup analyses could not explain the source of heterogeneity (Supplementary Table 3). Consequently, we just performed a narrative synthesis instead of combining the data for a meta-analysis (Supplementary Table 4).

Among these 38 RCTs, the results of 34 studies all indicated that CRP in MXSG plus WM group decreased more than that in WM group. However, there was no significant change on CRP in the remaining four studies.

##### 3.5.1.2 MXSG versus WM

Only one study (40 patients) evaluated the level of CRP by comparing MXSG with levofloxacin injection. The result showed that no significant difference was found between the two groups (*p* = 0.27).

##### 3.5.1.3 MXSG+WM 1 versus WM 1+WM 2

The other study compared MXSG plus moxifloxacin injection with moxifloxacin injection plus antipyretic and expectorants. Result showed that MXSG plus antibiotic had more reduction in CRP compared with control group (MD = −4.03 mg/L, 95% CI: −4.43 to 3.63; *p* < 0.00001). The concrete results of CRP were presented in Supplementary Table 4.

#### 3.5.2 White blood cell (WBC)

##### 3.5.2.1 MXSG+WM versus WM

33 studies (2435 participants) reported WBC level in this comparison type, among which the results of 23 studies showed that the level of WBC in MXSG plus WM group reduced more than in WM group. In the remaining 10 studies, the difference between two groups was not notable (Supplementary Table 4). We did not conduct a meta-analysis because of the high heterogeneity (I^2^ = 92%) even after subgroup analyses (Supplementary Table 3).

##### 3.5.2.2 MXSG versus WM

One RCT with 40 patients compared MXSG with levofloxacin injection on the level of WBC. The result indicated that there was no significant change between two groups (MD = −0.29 × 10^9^/L, 95% CI: −1.46 to 0.88; *p* = 0.63).

##### 3.5.2.3 MXSG+WM 1 versus WM 1+WM 2

The remaining one RCT (90 participants) took this comparison type. Compared with moxifloxacin injection plus antipyretic and expectorants, WBC level in MXSG plus moxifloxacin injection group reduced more (MD = −2.15 × 10^9^/L, 95% CI: −3.43 to −0.87; *p* = 0.001). More details of the results on WBC were in Supplementary Table 4.

#### 3.5.3 Procalcitonin (PCT)

##### 3.5.3.1 MXSG+WM versus WM

By comparing MXSG plus WM with WM alone, 28 studies including 2379 participants evaluated this outcome. All the results of 26 RCTs demonstrated that MXSG plus WM was superior to WM alone on decreasing the level of PCT. However, in the other two studies, no significant difference was observed between two groups (Supplementary Table 4). The heterogeneity (I^2^ = 95%) among studies was too high to perform meta-analysis (Supplementary Table 3).

##### 3.5.3.2 MXSG+WM 1 versus WM 1+WM 2

The result of the other study showed that MXSG plus moxifloxacin injection group had a lower level of PCT after treatment when compared with moxifloxacin injection plus antipyretic and expectorants (MD = −0.19 ng/ml, 95% CI: −0.29 to −0.09; *p* = 0.0003). The specific data of PCT could be seen in Supplementary Table 4.

#### 3.5.4 Absorption time of lung inflammation

Nine studies reported the absorption time of lung inflammation and applied X-rays to observe the change of lung radiograph.

##### 3.5.4.1 MXSG+WM versus WM

**FIGURE 9:**
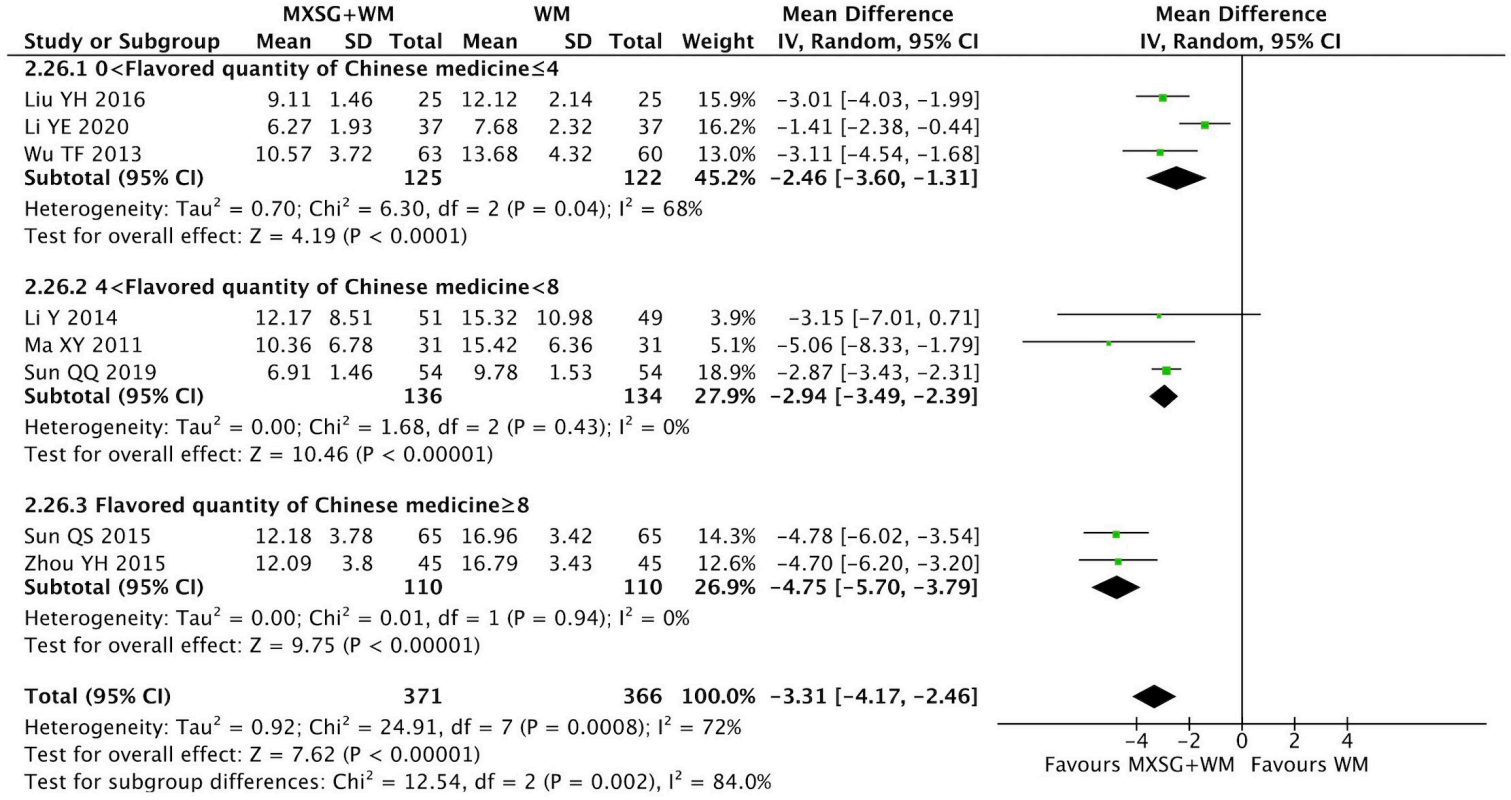
Forest plot of absorption time of lung inflammation by flavored quantity of Chinese medicine. Comparison: MXSG plus WM vs. WM. MXSG, Maxing Shigan Decoction; WM, western medicine.

Eight RCTs incorporating 687 participants evaluated this outcome. The pooled data indicated that the absorption of lung inflammation in MXSG plus WM group was faster than that in WM group (MD = −3.31 days, 95% CI: −4.17 to −2.46, *p* < 0.00001; I^2^ = 72%) (Figure 9).

When the flavored quantity of Chinese medicine was over eight, the combination of MXSG and WM seemed to be more effective in promoting the absorption of lung inflammation (MD = −4.75 days, 95% CI: −5.70 to −3.79, *p* < 0.00001; I^2^ = 0%). Besides, the heterogeneity decreased in each subgroup (Supplementary Table 3).

##### 3.5.4.2 MXSG+WM 1 versus WM 1+WM 2

Only one study compared MXSG plus moxifloxacin injection with moxifloxacin injection plus symptomatic treatment on this outcome. The data indicated that there was no statistical difference between two groups in the absorption time of lung inflammation (MD = −4.17 days, 95% CI: −8.43 to 0.09, *p* = 0.06).

#### 3.5.5 Improvement rate of chest radiograph

For this outcome, 35 studies applied X-rays to observe the absorption of lung inflammation, eight studies used chest CT, one study applied both measurement methods at the same time, and the remaining one study did not report the measurement method.

##### 3.5.5.1 MXSG+WM versus WM

A total of 42 RCTs involving 2244 participants measured this outcome. The heterogeneity among studies was high (I^2^ = 73%) and we could not find the source of it by the predefined subgroup analyses (Supplementary Table 3). Among these RCTs, 25 studies declared that the difference in the improvement rate of chest radiographs was not significant between two groups. However, results of the remaining 17 studies demonstrated that MXSG plus WM was better in improving the chest radiograph of CAP patients than WM alone (Supplementary Table 4).

##### 3.5.5.2 MXSG versus WM

One study compared the treatment of MXSG with levofloxacin injection and the improvement rates of chest radiographs in both groups were 100%.

##### 3.5.5.3 MXSG+WM 1 versus WM 1+WM 2

One study took this comparison type and there was no statistical difference between the treatment of MXSG plus moxifloxacin injection and moxifloxacin injection plus antipyretic and expectorants on the improvement rate of chest radiograph (RR = 1.05, 95% CI: 0.96 to 1.15, *p* = 0.29).

##### 3.5.5.4 MXSG versus placebo

Only one study took this comparison type and no significant change was found between MXSG group and placebo group on this outcome (RR = 1.04, 95% CI: 0.87 to 1.24, *p* = 0.69). The details of improvement rate of chest radiographs were presented in Supplementary Table 4.

#### 3.5.6 Lung function: forced vital capacity (FVC)

Five RCTs compared MXSG plus WM with WM alone on this outcome. Among them, two studies demonstrated that MXSG plus WM group had a better effect on FVC than WM group. However, the other two studies showed that FVC in WM group improved more than MXSG plus WM group. And in the remaining one study, the difference between groups was not significant (Supplementary Table 4). Heterogeneity among studies was still high even after subgroup analyses, so meta-analysis was failed to perform (I^2^ = 98%) (Supplementary Table 3).

#### 3.5.7 Lung function: forced expiratory volume in the first second (FEV1)

**FIGURE 10:**
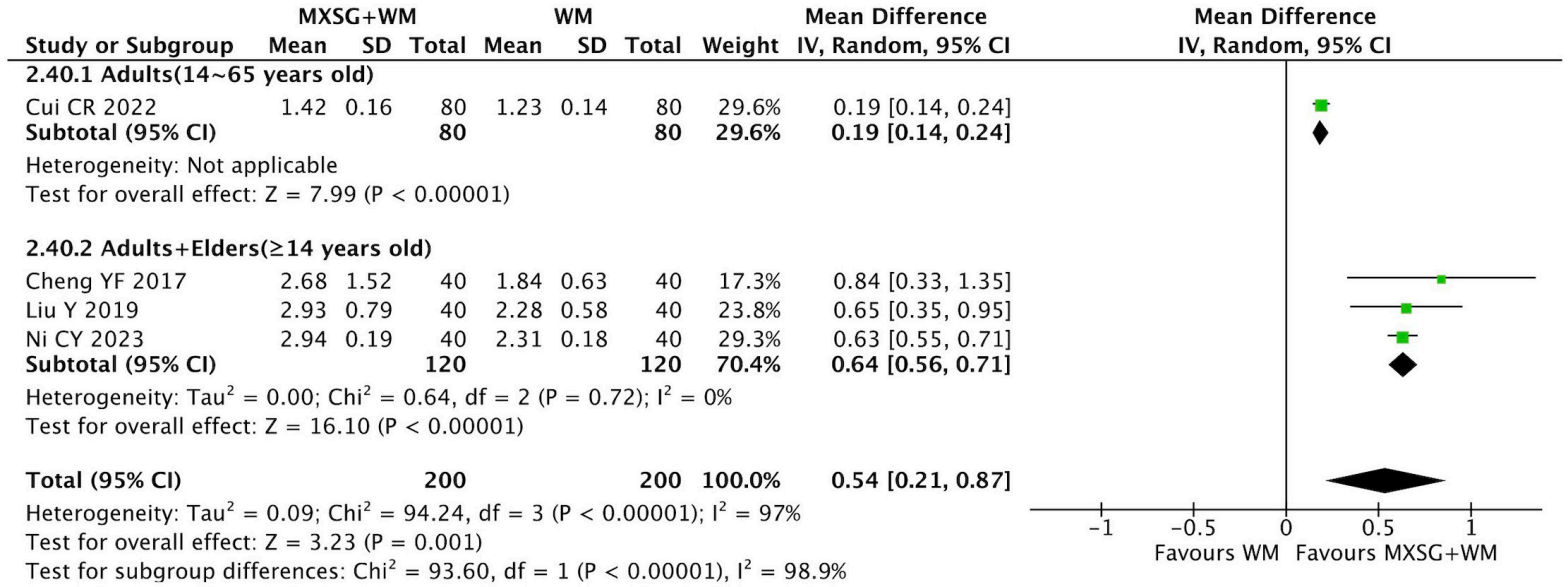
Forest plot of FEV1 by age. Comparison: MXSG plus WM vs. WM. MXSG, Maxing Shigan Decoction; WM, western medicine.

Four RCTs reported this outcome. The data indicated that MXSG plus WM had a better effect on FEV1 than WM alone (MD = 0.54L, 95% CI: 0.21 to 0.87, *p* = 0.001; I^2^ = 97%) (Figure 10). Heterogeneity in each group had an obvious reduction when subgroup analysis was classified by age. Furthermore, we found that MXSG plus WM seemed to be more effective for adults plus elders group on FEV1 compared with WM alone (MD = 0.64L, 95% CI: 0.56 to 0.71, *p* < 0.00001; I^2^ = 0%) (Supplementary Table 3).

#### 3.5.8 Lung function: peak expiratory flow (PEF)

Two studies compared MXSG plus WM with WM on this outcome. The results of both studies indicated that the level of PEF in MXSG plus WM group was higher than that in WM group after treatment. However, the heterogeneity was too high to pool the data (I^2^ = 97%) (Supplementary Tables 3, 4).

#### 3.5.9 Length of hospitalization

**FIGURE 11:**
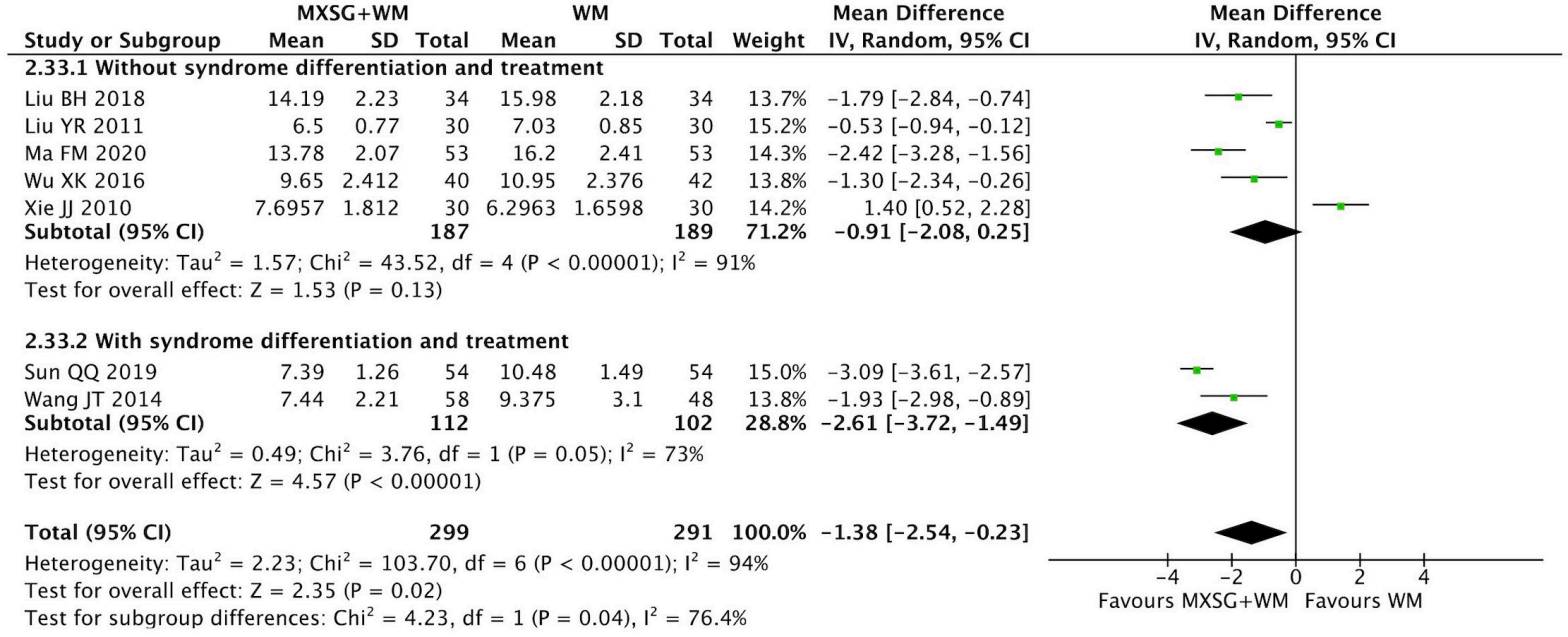
Forest plot of length of hospitalization by whether to take syndrome differentiation and treatment. Comparison: MXSG plus WM vs. WM. MXSG, Maxing Shigan Decoction; WM, western medicine.

Seven RCTs involving 590 participants evaluated the length of hospitalization. The results demonstrated that MXSG plus WM may shorten the length of hospitalization when compared with WM alone (MD = −1.38 days, 95% CI: −2.54 to −0.23, *p* = 0.02; I^2^ = 94%) (Figure 11).

We found that heterogeneity within each group reduced in subgroup analysis based on whether to take syndrome differentiation and treatment, but substantial heterogeneity (I^2^ = 91%) remained in the “without syndrome differentiation and treatment” subgroup. Furthermore, MXSG plus WM may show better effect on reducing the hospitalization time when taking syndrome differentiation and treatment (MD = −2.61 days, 95% CI: −3.72 to −1.49, *p* < 0.00001; I^2^ = 73%). Besides, age and the flavored quantity of Chinese medicine failed to substantially reduce heterogeneity within each subgroup (Supplementary Table 3). Sensitivity analysis revealed that when the studies by Xie JJ (83), Liu YR (49) and Sun QQ (69) were excluded, the overall heterogeneity decreased to I^2^ = 0%, indicating that these studies likely contributed to the observed heterogeneity. Further analysis of study characteristics demonstrated that the high heterogeneity may originate from the variation in antibiotic selection within western medicine regimens, the differences in herb dosage and compatibility during TCM interventions and the methodological limitation arising from ill-defined discharge criteria.

#### 3.5.10 Mortality

Only one RCT recorded the outcome of mortality. There was no statistically significant difference in mortality between MXSG plus WM and WM alone (RR = 0.25, 95% CI: 0.06 to 1.08; *p* = 0.06).

### 3.6 Sensitivity analysis

The result of sensitivity analysis revealed that most of our findings were robust. However, for incidence of adverse events, the significance of difference (*p* = 0.06) changed to *p* ≤ 0.04 after excluding the studies of Cui CR (24) (*n* = 160, RR = 1.33, 95% CI: 0.48 to 3.67, *p* = 0.58), Kong FH (38) (*n* = 120, RR = 1.60, 95% CI: 0.56 to 4.61, *p* = 0.38), Liu YF (51) (*n* = 62, RR = 7.00, 95% CI: 0.38 to 130.1, *p* = 0.19) and Zhang (92) (*n* = 80, RR = 2.00, 95% CI: 0.19 to 21.18, *p* = 0.56), respectively. We found that the results of these studies all indicated the incidence of adverse events in MXSG plus WM group might be higher than WM group, but the difference was not statistically significant. By analyzing the study characteristics, we found the potential reasons for the instability of results: First, these studies had relatively small or moderate sample sizes compared to the other studies. The instability of their results might have an impact on the point estimate and confidence interval, especially when the event occurrence rate was already low; Second, the excluded studies had inconsistent definitions, collection and reporting of adverse events. Cui CR 2022 (24) and Kong FH 2023 (38) captured symptomatic adverse events, Zhang JJ 2020 (92) only reported the specific laboratory indicator abnormality of “serum urea nitrogen” as an adverse event, while Liu YF 2023 (51) reported unspecified events. Therefore, the certainty of findings may be influenced to some extent and our conclusion should be treated with caution (Supplementary Figure 1).

### 3.7 Publication bias

Egger’s test results indicated that there was no evidence of publication bias in the resolution time of fever (*p* = 0.156), resolution time of cough (*p* = 0.095) and the incidence of adverse events (*p* = 0.550). However, the resolution time of pulmonary crepitation had a significant publication bias (*p* = 0.019) (Supplementary Figure 2). And the above results were basically consistent with funnel plots (Supplementary Figure 3). Publication bias was failed to detected for other outcomes because of the insufficient number of the included studies.

### 3.8 Trial sequential analysis

According to TSA, Z-curve of length of hospitalization crossed conventional significant threshold, but didn’t reach the TSA line and RIS line, suggesting the existence of a false positive result. Z-curves of the incidence of adverse events neither crossed the conventional significant threshold and the futility boundaries, nor reached the RIS line, indicating the possibility of a false negative error and the need for more trials to prove the conclusion. TSA of other outcomes didn’t show false positive or negative error (Supplementary Figure 4).

### 3.9 Certainty of evidence

**FIGURE 12:**
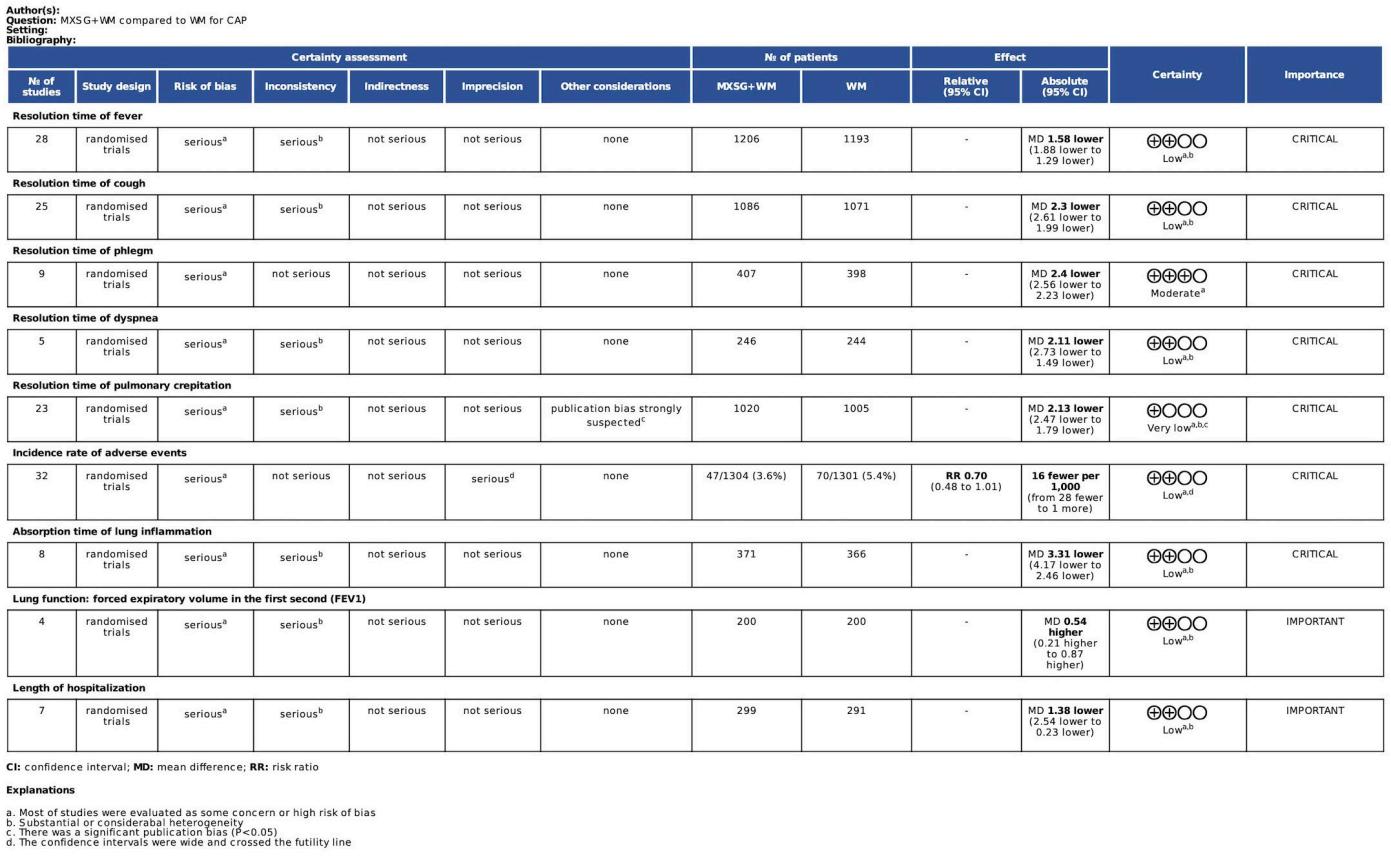
Certainty of evidence.

Grade method was applied to evaluate the certainty of evidence for all important outcomes. Certainty of evidence was rated as moderate or low mainly due to the risk of bias (lack of allocation concealment and blinding as well as the selective reporting), inconsistency (I square value was high), imprecision and the potential publication bias. More details about the certainty assessment were shown in Figure 12.

## 4 Discussion

### 4.1 Summary of main findings

This review demonstrated that compared with WM alone, the combination of MXSG and WM may have potential effective on decreasing the length of hospitalization as well as the duration of symptoms including fever, cough, phlegm, dyspnea, and pulmonary crepitation by 1∼3 days. It may also promote the absorption of lung inflammation and improve lung function better than WM alone. However, these findings should be interpreted with caution given the moderate-to-low certainty of evidence. For mortality, there was no statistically significant difference between MXSG plus WM and WM alone. The heterogeneities among studies on laboratory indicators and the improvement rate of chest radiographs were too high to pool the data. In addition, MXSG alone seemed to have a better antipyretic effect than placebo, but it didn’t show statistical difference in other outcomes compared with either WM group or placebo group. Regarding safety, no serious adverse events occurred during treatment and the incidence of adverse events in MXSG plus WM group was not higher than WM group.

### 4.2 Interpretation of the results

The pathogenesis of CAP primarily involved local and systemic inflammatory responses caused by pathogen invasion. These inflammatory responses accounted for most clinical symptoms, physical signs, and abnormalities in laboratory and imaging findings (99). In this regard, MXSG demonstrated anti-inflammatory and antipyretic effects by inhibiting leukocyte adhesion, suppressing the release of inflammatory factors and inflammatory cell infiltration, as well as improving endotoxin-induced pulmonary interstitial edema (100). Additionally, MXSG played an anti-infection effect by inhibiting the proliferation of pathogens, blocking the storm of inflammatory factors, and improving the imbalance of intestinal flora to show (101). Furthermore, MXSG presented a positive effect on relieving cough and asthma by inhibiting the release of allergic substances, reducing bronchial epithelial damage, and relieving bronchospasm (102). Consequently, adding MXSG with antibiotics had a better effect on relieving fever, cough and dyspnea, promoting the absorption of pulmonary inflammation as well as improving lung function, thereby potentially reducing hospitalization length. However, we couldn’t draw a conclusion of the effectiveness of MXSG on the laboratory indicators due to the high heterogeneity, which may result from the different detection methods, treatment course, detection time, and disease baseline in each study. Meanwhile, the influence of MXSG on mortality was also uncertain, because most of the included studies didn’t report this outcome.

For subgroup analyses, we found that when compared with WM alone, the effect of MXSG plus WM was more notable for elders on reducing the duration of fever (−3.12 vs. −1.45 days, interaction *p* < 0.00001). In addition, for adults plus elders group, MXSG plus WM significantly decreased the duration of pulmonary crepitation (−3.41 vs. −1.88 days, interaction *p* < 0.00001) and improved FEV1 (0.64L vs. 0.19L, interaction *p* < 0.00001). These findings suggested that age may influence the effectiveness of MXSG, possibly due to the different clinical features including etiology, risk factors, comorbidities, severity, and clinical presentation for CAP patients at different ages (103, 104). Besides, MXSG plus WM shortened the length of hospitalization more obviously in subgroup receiving syndrome differentiation and treatment (−2.61 vs. −0.91 days, interaction *p* = 0.04). TCM syndrome differentiation was known as a comprehensive analysis of clinical information obtained from observation, listening, questioning, and pulse (105). It provided more individual therapeutic schedules which could resolve different symptoms of each patient with pertinence, so that improving the effectiveness of intervention. In addition, the effectiveness of MXSG plus WM was more significant in relieving dyspnea when the flavored quantity of Chinese medicine was four to eight (−2.61 vs. −1.46 days, interaction *p* < 0.00001) and in reducing the absorption time of lung inflammation when the flavored quantity was over eight (−4.75 vs. −2.71 days, interaction *p* = 0.0008). The results indicated that compared with S-MXSG, M-MXSG seemed to be more suitable for complex clinical conditions in reality, because it contained more Chinese herbal medicine with different therapeutic actions. However, since subgroup analyses were exploratory and observational with no causal inferences should be drawn, the results only suggested potential associations between study characteristics and the intervention effect, and further studies were needed to verify the conclusion.

By analyzing the composition of M-MXSG, we found that in addition to the four fixed Chinese herbal medicine, the following Chinese herbal medicine were often added in clinical practice to achieve a synergistic effect: First, Heat-clearing and detoxifying Chinese herbal medicine, such as Baikal Skullcap Root (Huangqin, *Scutellaria baicalensis Georgi*) and Honeysuckle Flower (Jinyinhua, *Lonicera japonica Thunb.*), which have the antipyretic, anti-inflammatory, antibacterial and antiviral effects in pharmacology (106–108); Second, Wind-heat-dispersing Chinese herbal medicine, such as Peppermint Leaf (Bohe, *Mentha haplocalyx Briq.*) and Great Burdock Fruit (Niubangzi, *Arctium lappa L*.), demonstrating pharmacological effects such as diaphoretic, antipyretic, anti-pathogenic activity and anti-allergic action (109–111); Third, Lung-fire-clearing and asthma-relieving Chinese herbal medicine, such as Pepperweed Seed (Tinglizi, *Lepidium apetalum Willd.*) and White Mulberry Root Bark (Sangbaipi, *Morus alba L.*), which have pharmacological effects including relieving asthma, cough suppression and diuresis (112, 113); Forth, Cough-relieving and expectorant Chinese herbal medicine, such as Platycodon Root [Jiegeng, *Platycodon grandiflorus (Jacq.) A.DC.]* and Stemona Root [Baibu, *Stemona sessilifolia (Miq.) Miq.]*, which have expectorant, cough-relieving and anti-inflammatory effects (114–116) (Supplementary Table 1).

### 4.3 Strengths and limitations

This systematic review presented three key methodological strengths: First, we have collected and extracted abundant clinical data. For example, we incorporated all Chinese herbal decoctions based on MXSG and a total of four comparison types. Second, we chose proper statistical methods to analyze data and deal with the heterogeneity among studies. Considering the diversity of clinical heterogeneity, we predefined several subgroup analyses to solve it and tried to explore more specific information to provide a reference for clinical practice. The subgroup analyses not only covered the common characteristics of participants but also considered the particularity of TCM treatment especially the influence of additional Chinese herbal medicine in MXSG as well as TCM syndrome differentiation and treatment. Furthermore, we observed that the grouping factors may be the source of heterogeneity, which confirmed our suspicions.

However, there were also some limitations. First, all studies were carried out in China which may result from that MXSG was mainly used in China and has not been popularized abroad. Second, the methodology quality of the included RCTs was generally medium to low, which resulted from the inadequate reporting of randomization process, the lack of double-blind method and the loss of registration protocol. As a consequence, the risk of bias was relatively high, resulting in a reduction on the certainty of conclusion. Third, the clinical heterogeneity of the included studies was high, which brought challenges to quantitative analysis and even resulted in the failure to conduct meta-analysis. Moreover, due to the differences in the composition and dosage of MXSG as well as TCM syndrome differentiation and treatment, it was hard to repeat the research and made the standardization of TCM difficult. The above differences mainly arose from the different ages and conditions of participants in each study, as well as the different clinical experiences and medication habits of physicians, which may lead to great clinical heterogeneity. Furthermore, this was also the problem and challenge of clinical trials related to TCM compounds. However, we could not thoroughly deny the effectiveness of TCM because of its diversity. The same and standardized therapy was not necessarily the most effective for each individual. And the unique feature of TCM precisely lies in its individualized treatment plan such as the modification in formulation as well as syndrome differentiation and treatment. Consequently, while our findings provided preliminary evidence regarding the effectiveness of MXSG for CAP, the study conclusion should be treated with appropriate caution because of these limitations.

### 4.4 Comparison with previous studies

Current systematic reviews of MXSG on CAP demonstrated that MXSG could shorten the duration of fever, cough, phlegm, pulmonary crepitation, and the absorption time of lung inflammation (13, 14, 117–119). The relevant evidence also showed that MXSG may reduce infection indices (such as CAP, WBC and PCT) and improve effective rate. Moreover, none of the reviews reported serious adverse reactions which could reflect the safety of MXSG. In conclusion, these findings were consistent with our review, which confirmed that MXSG may bring benefits in relieving the symptoms and signs of CAP patients with a great safety.

In contrast to previous studies with similar subject, our review included a larger amount of RCTs with wider research range. Meanwhile, this was an update and supplement to systematic reviews on MXSG for patients with CAP. Additionally, we included various comparison types including MXSG alone or MXSG plus WM in treatment groups and WM or placebo in control groups. Last but not least, we also designed several subgroup analyses based on the clinical diversity and characteristics of TCM therapy to explore the source of heterogeneity, which could enrich the thoughts for research and clinical treatment.

### 4.5 Implications

In future clinical practice, MXSG was suggested to be used in treating CAP as an adjunctive therapy to antibiotics. Importantly, the application of MXSG should adhere to TCM syndrome differentiation principle. The kind and dosage of Chinese herbal medicine were supposed to be adjusted according to the specific symptoms of patients, rather than employing a standardized formulation for all patients. In order to achieve a better curative effect, we recommended that based on S-MXSG, more Chinese herbal medicine demonstrating the effect of clearing lung heat, reducing phlegm or relieving cough should be added to the decoction.

To enhance the methodological quality, we advised strict adherence to CONSORT statement for the reporting of RCTs. It was also suggested that researchers register a protocol in advance and record the URL (Uniform Resource Locator) in articles to decrease the reporting bias. In order to improve the quality of future clinical trials, random sequence generation and allocation concealment were advised to be conducted strictly referring to Cochrane Handbook17. Besides, TCM placebo had better be given to control group to realize the blinding of participants and researchers. And the outcome of follow-up was supposed to be added in future trials to observe long-term curative effect of MXSG on CAP, such as mortality. This series of measures would improve the methodology quality and enhance the certainty of conclusion.

In consideration of the variety of TCM treatment, researchers could conduct dose-effect analysis by designing clinical trials to explore whether different dosages and ratios of Chinese herbal medicine would influence the effectiveness of MXSG on CAP patients. It was also recommended to perform data mining to analyze the composition principles of MXSG applied in modern times, which could provide guiding value for clinical practice. Furthermore, future clinical studies based on real world should also research the influence of TCM syndrome differentiation and treatment on the effectiveness of MXSG.

Traditional Chinese medicine clinical research is facing a great methodological challenge due to the complex composition principle and dose-effect relationship of TCM compounds. To address these challenges, we propose the following research strategies: First, future researchers should establish classification criteria for TCM formulations and clinical evidence reporting standards, so that TCM clinical research is consistent with TCM theories and reflects the general principles of evidence-based medicine at the same time. Second, future clinical research on TCM formulations need to be implemented gradually in phases on the premise of basic experiments, and the research methods should be continuously innovated to facilitate effective translation and promotion of clinical evidence for TCM compounds.

## 5 Conclusion

Evidence of limited quality indicated that in contrast to WM, MXSG combined with WM may have potential positive effect on the treatment of CAP with a good safety. The subgroup analyses indicated that age, TCM syndrome differentiation and treatment as well as the flavored quantity of Chinese medicine may be the sources of clinical heterogeneity among studies. Because of the poor methodological quality and substantial heterogeneity, the evidence supporting our findings remained uncertain. Additionally, due to the certainty of evidence was moderate or low, the results of this review were suggested to be interpreted and applied with caution. Future TCM clinical trials should pay attention to the methodological quality to improve the reliability of evidence. Furthermore, researchers were advised to explore whether the composition, dose, flavored quantity, TCM syndrome differentiation and other relevant factors would influence the effectiveness of MXSG on CAP, which could optimize its clinical application for CAP management.

## Data Availability

The original contributions presented in this study are included in this article/Supplementary material, further inquiries can be directed to the corresponding author.

## Footnotes

1
https://www.gradepro.org/
