# Supplementary material for: Effectiveness and safety of Maxing Shigan Decoction for community-acquired pneumonia: a systematic review and meta-analysis of randomized controlled trials
Source: Front Med (Lausanne). 2025 Aug 29;12:1639027. doi: 10.3389/fmed.2025.1639027 (PMC12425732; doi:10.3389/fmed.2025.1639027)
Supplement: Supplementary file 1 [file Table_1.docx]

Supplementary Material

**Table of contents**

[1 Supplementary Figures 2](#_Toc198402730)

[1.1 Supplementary Figure S1. Sensitivity analysis 2](#_Toc198402731)

[1.2 Supplementary Figure S2. Egger’s test 7](#_Toc198402732)

[1.3 Supplementary Figure S3. Funnel plot. 9](#_Toc198402733)

[1.4 Supplementary Figure S4. Trial sequential analysis. 9](#_Toc198402734)

[2 Supplementary Table 17](#_Toc198402735)

[2.1 Supplementary Table S1. The role of Chinese herbal medicine in the included MXSG 17](#_Toc198402736)

[2.2 Supplementary Table S2. Composition of Chinese herbal formula in the 81 included studies 24](#_Toc198402737)

[2.3 Supplementary Table S3. Details of subgroup analyses (MXSG+WM vs WM) 32](#_Toc198402738)

[2.4 Supplementary Table S4. Summary of results on other outcomes 38](#_Toc198402739)

[3 Supplementary Files 47](#_Toc198402740)

[3.1 Supplementary File S1. Search strategy 47](#_Toc198402741)

# Supplementary Figures

## Supplementary Figure S1. Sensitivity analysis

**A**

**B**

**C**

**D**

**E**

**F**

**G**

**H**

**I**

**(A)** Resolution time of fever in comparison of MXSG plus WM vs. WM

**(B)** Resolution time of cough in comparison of MXSG plus WM vs. WM

**(C)** Resolution time of phlegm in comparison of MXSG plus WM vs. WM

**(D)** Resolution time of dyspnea in comparison of MXSG plus WM vs. WM

**(E)** Resolution time of pulmonary crepitation in comparison of MXSG plus WM vs. WM

**(F)** Incidence of adverse events in comparison of MXSG plus WM vs. WM

**(G)** Absorption time of lung inflammation in comparison of MXSG plus WM vs. WM

**(H)** FEV1 in comparison of MXSG plus WM vs. WM

**(I)** Length of hospitalization in comparison of MXSG plus WM vs. WM

## Supplementary Figure S2. Egger’s test

**A**

**B**

**C**

**D**

**(A)** Resolution time of fever in comparison of MXSG plus WM vs. WM

**(B)** Resolution time of cough in comparison of MXSG plus WM vs. WM

**(C)** Resolution time of pulmonary crepitation in comparison of MXSG plus WM vs. WM

**(D)** Incidence of adverse events in comparison of MXSG plus WM vs. WM

## Supplementary Figure S3. Funnel plot.

A B

C D

**(A)** Resolution time of fever in comparison of MXSG plus WM vs. WM

**(B)** Resolution time of cough in comparison of MXSG plus WM vs. WM

**(C)** Resolution time of pulmonary crepitation in comparison of MXSG plus WM vs. WM

**(D)** Incidence of adverse events in comparison of MXSG plus WM vs. WM

## Supplementary Figure S4. Trial sequential analysis.

**A**


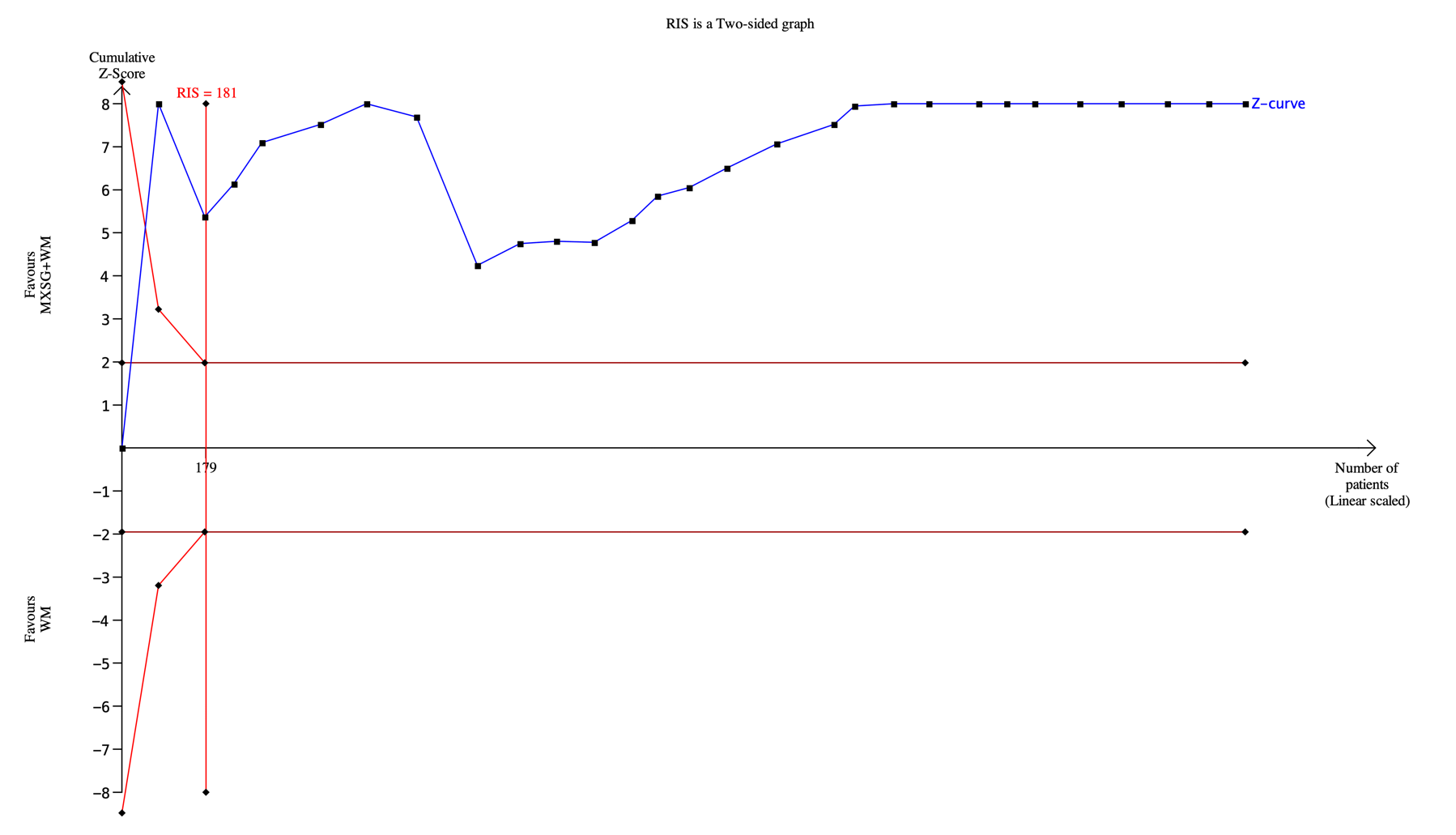


**B**


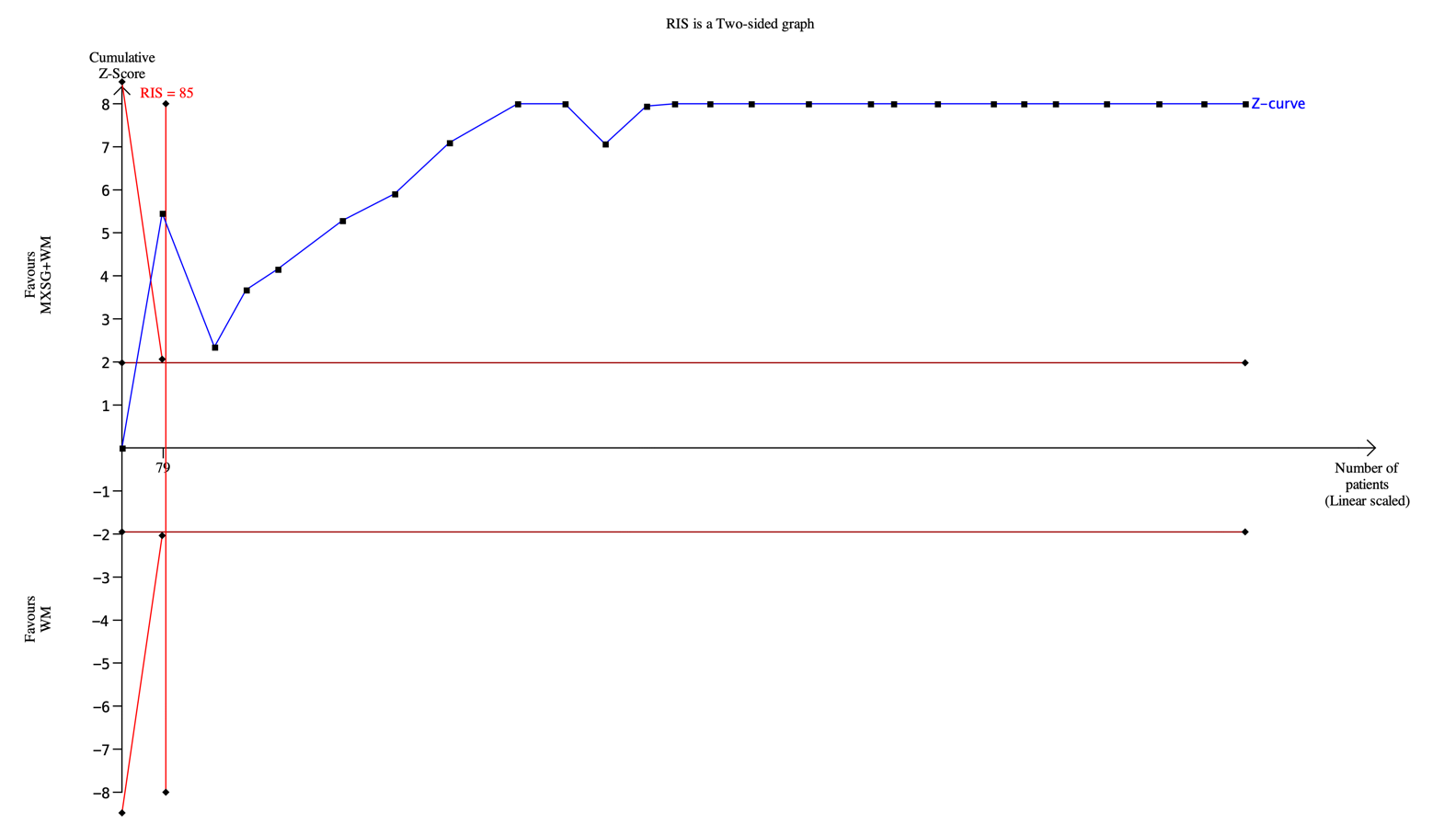


**C**


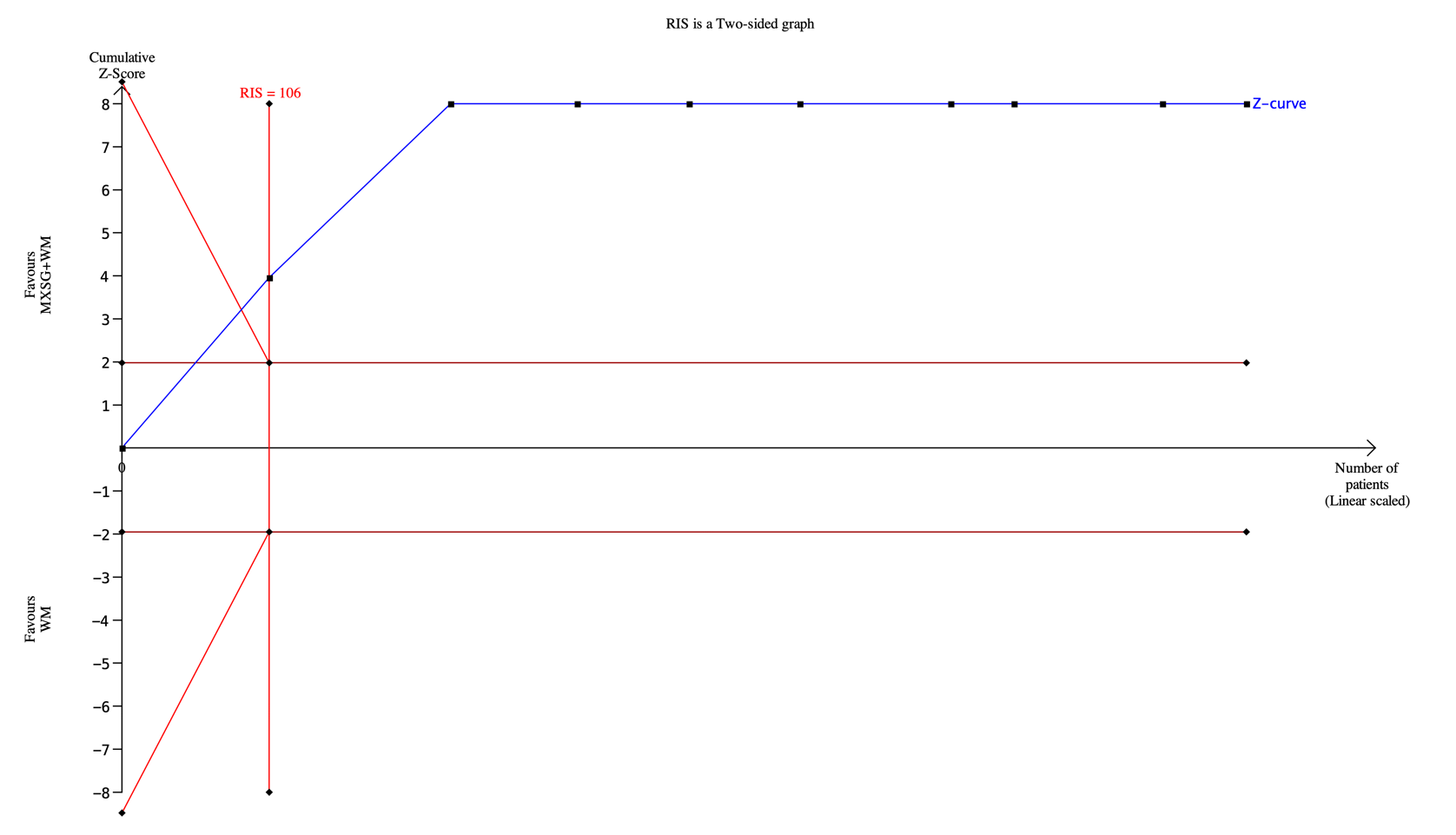


**D**


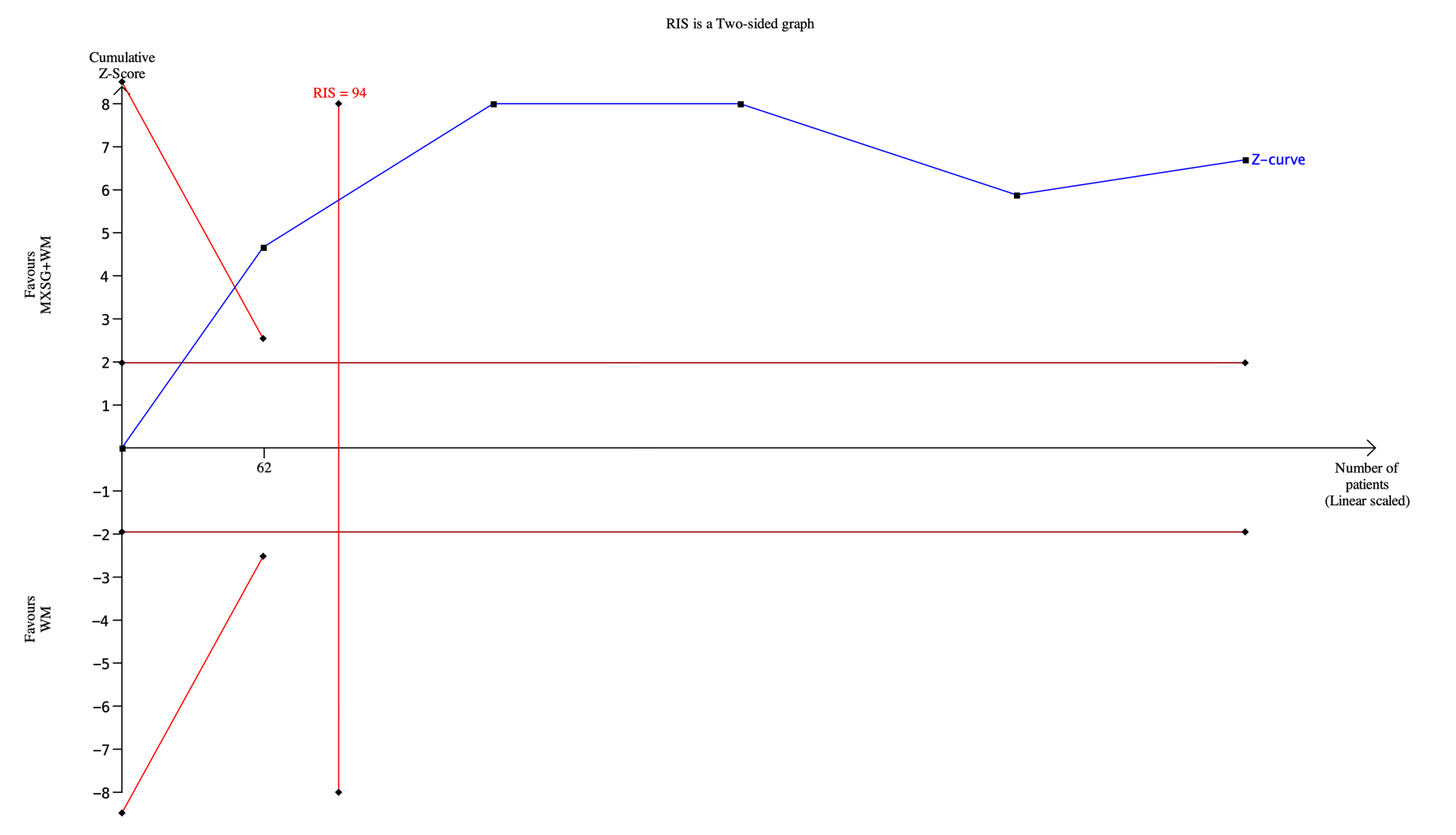


**E**


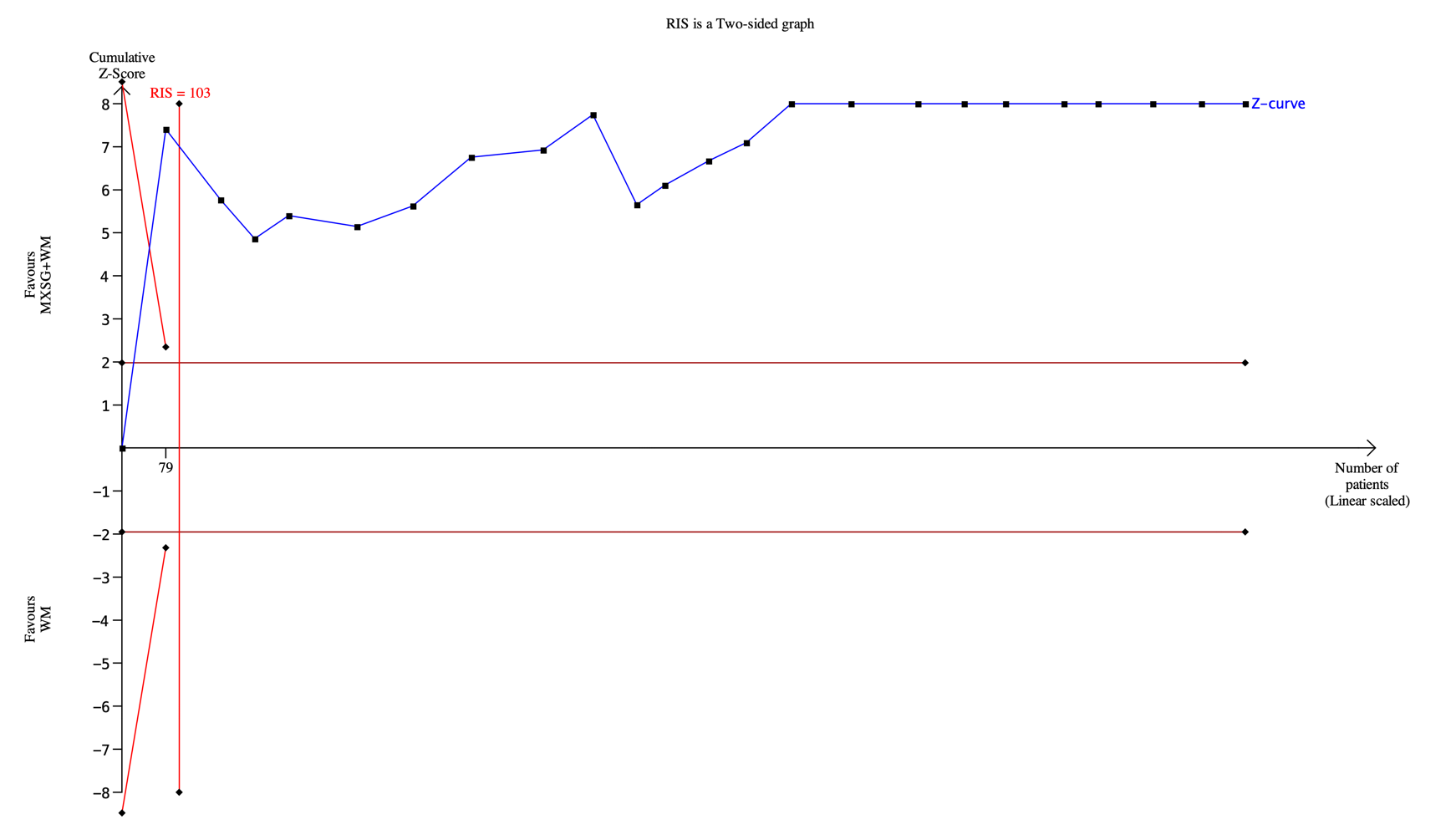


**F**


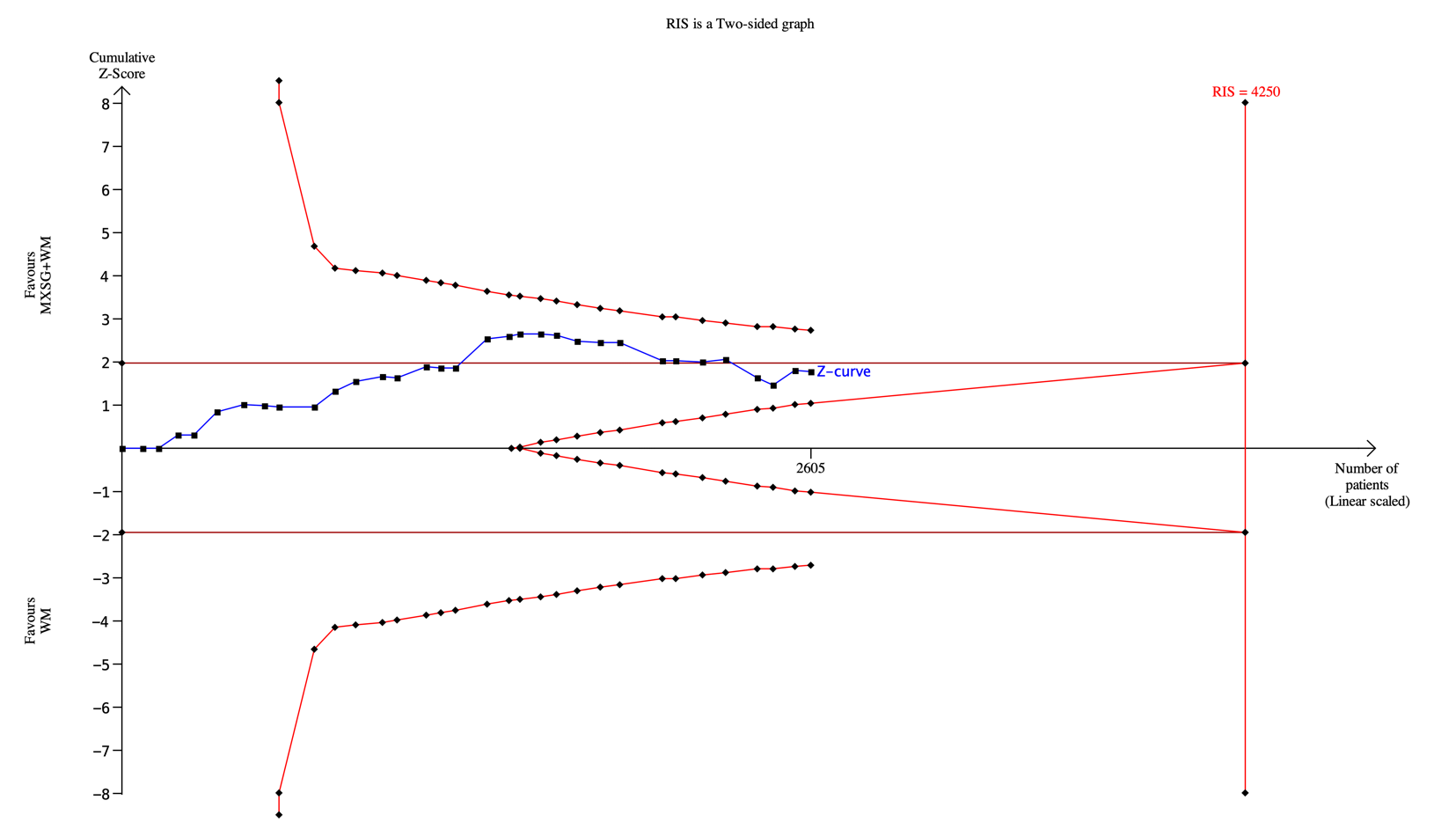


**G**


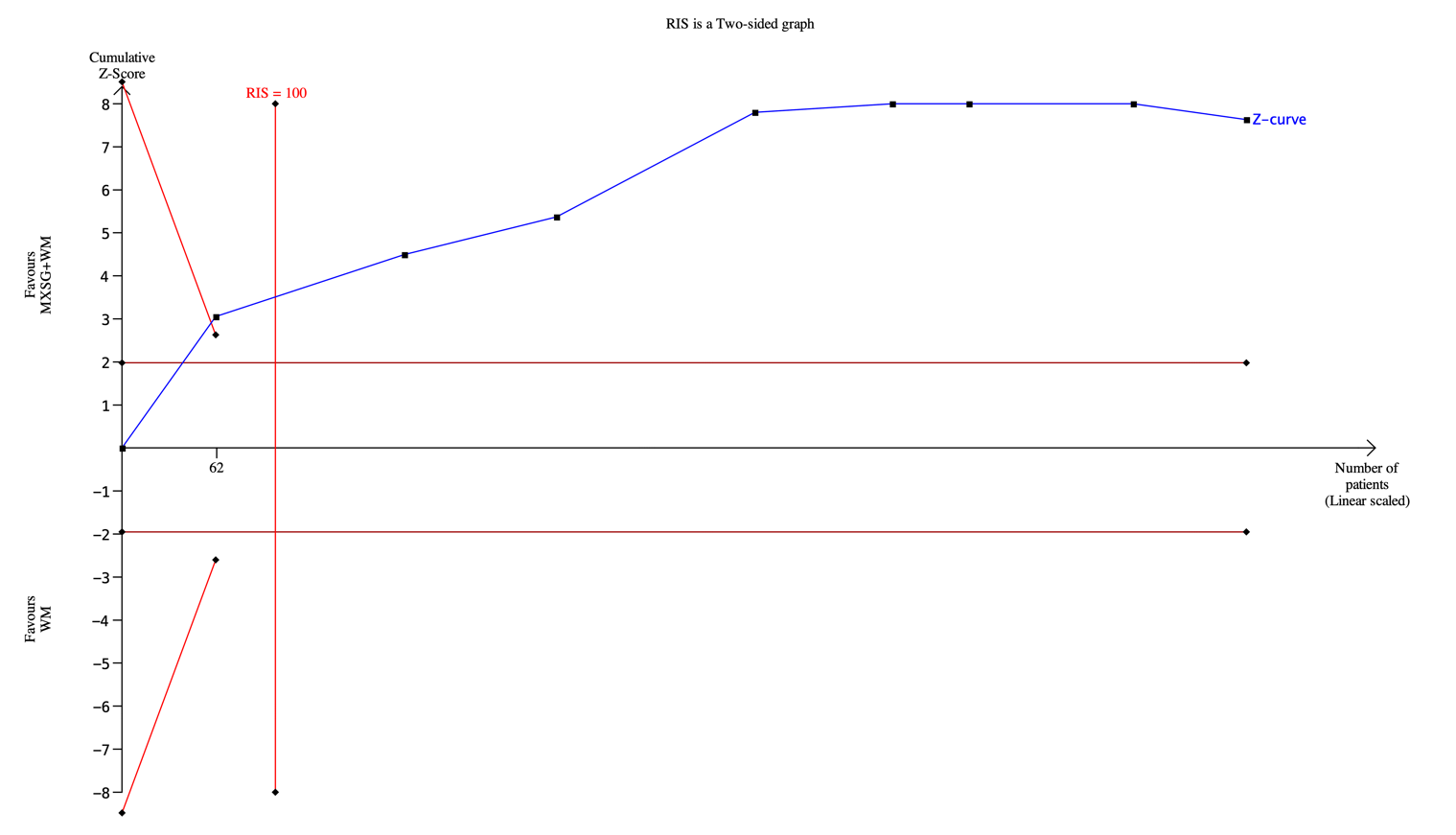


**H**


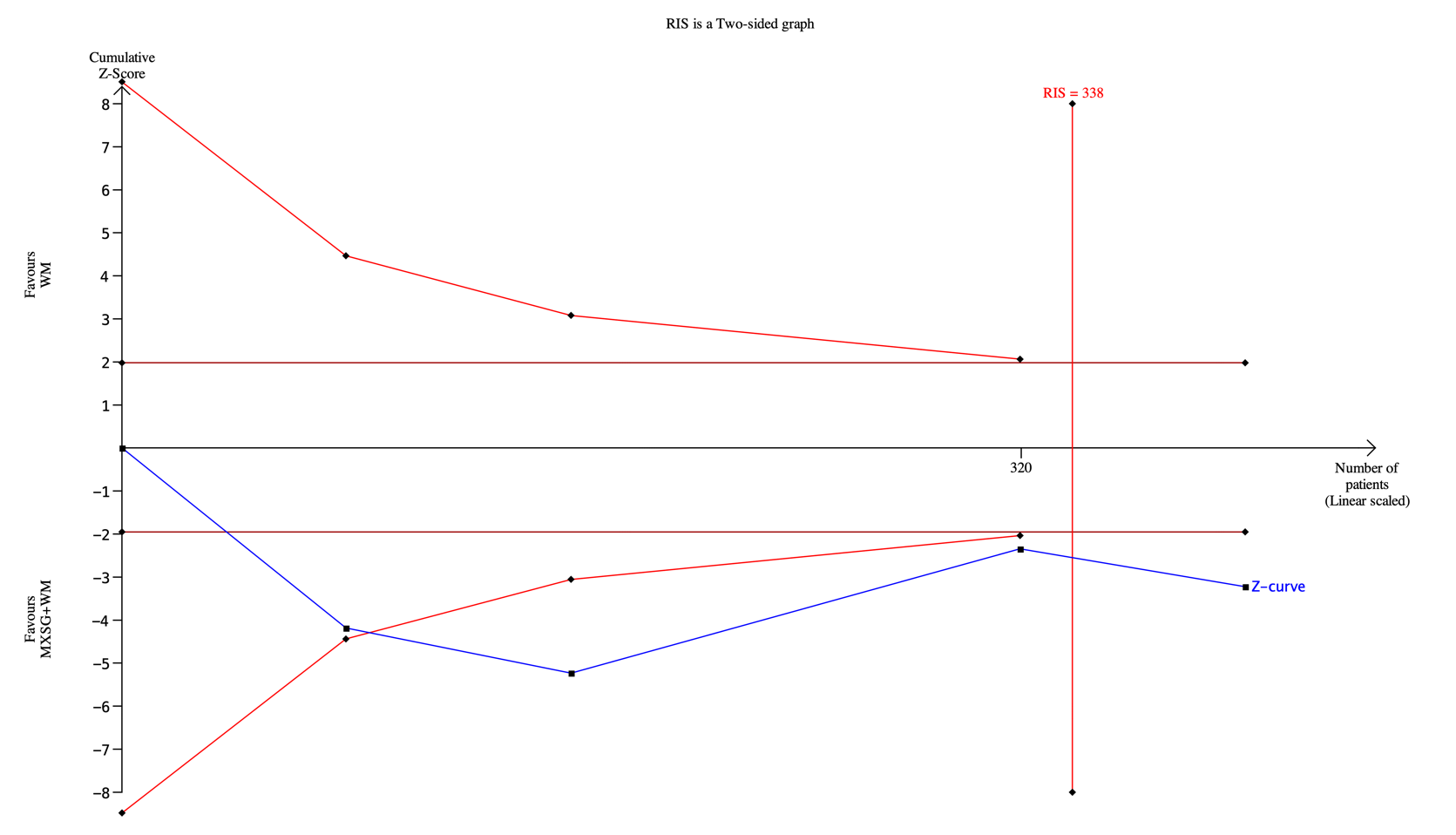


**I**


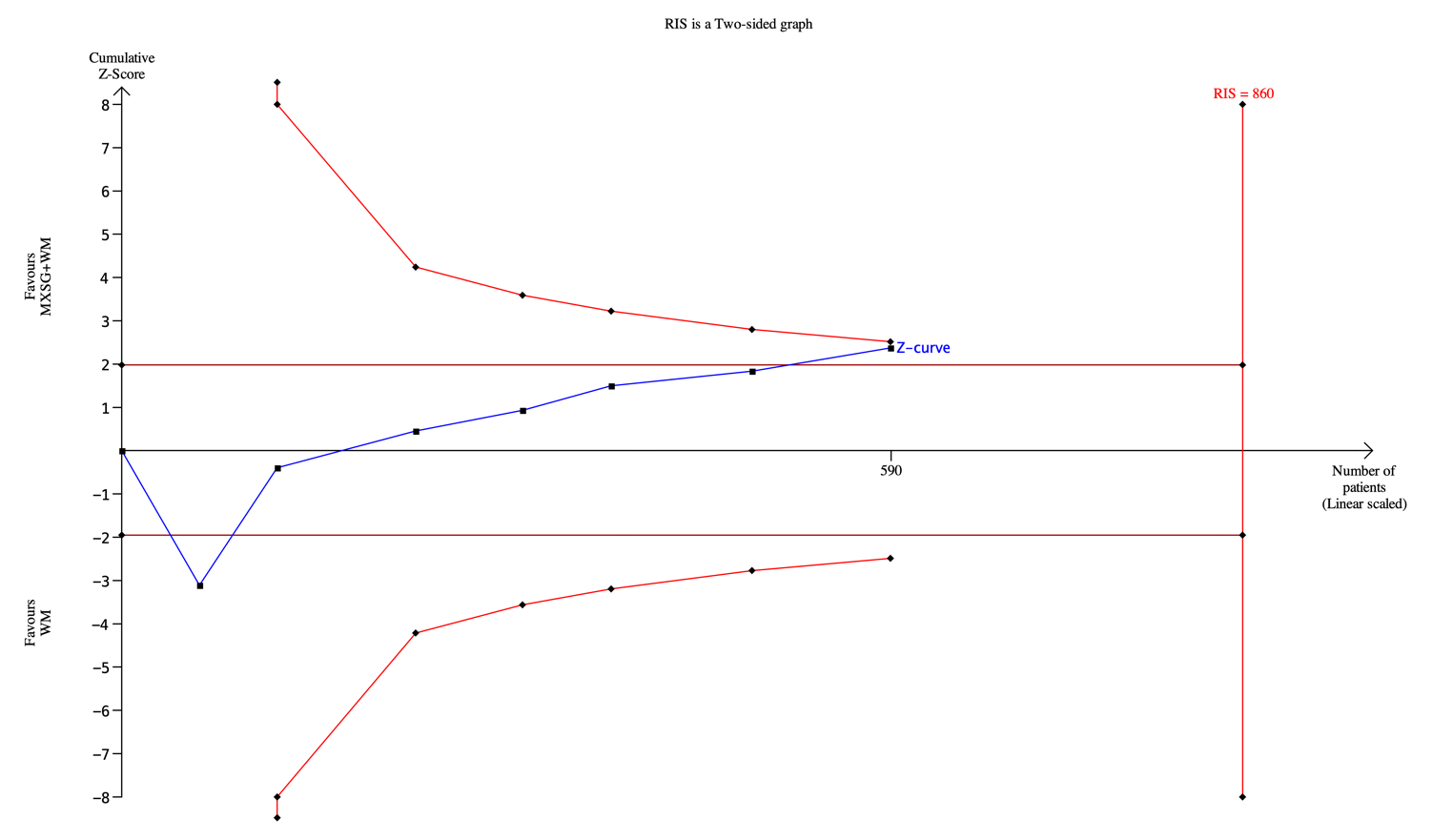


**(A)** Resolution time of fever in comparison of MXSG plus WM vs. WM

**(B)** Resolution time of cough in comparison of MXSG plus WM vs. WM

**(C)** Resolution time of phlegm in comparison of MXSG plus WM vs. WM

**(D)** Resolution time of dyspnea in comparison of MXSG plus WM vs. WM

**(E)** Resolution time of pulmonary crepitation in comparison of MXSG plus WM vs. WM

**(F)** Incidence of adverse events in comparison of MXSG plus WM vs. WM

**(G)** Absorption time of lung inflammation in comparison of MXSG plus WM vs. WM

**(H)** FEV1 in comparison of MXSG plus WM vs. WM

**(I)** Length of hospitalization in comparison of MXSG plus WM vs. WM

# Supplementary Table

## Supplementary Table S1. The role of Chinese herbal medicine in the included MXSG

| Chinese herbal medicine | Frequency of usage | Function |
| --- | --- | --- |
| Ephedrae Herba | 49 | Effuse sweat, dissipate cold; diffuse the lung, calm panting; disinhibit water, disperse swelling |
| honey-fried Ephedrae Herba | 32 | Effuse sweat, dissipate cold; diffuse the lung, calm panting; disinhibit water, disperse swelling |
| Armeniacae Semen Amarum | 81 | Direct qi downward, suppress cough, calm panting; moisten the intestines, free the stool |
| Gypsum Fibrosum | 81 | Clear heat, purge fire; eliminate vexation, allay thirst |
| Glycyrrhizae Radix et Rhizoma | 49 | Tonify the spleen, tonify qi; clear heat, detoxify; dispel phlegm, suppress cough; relax tension, relieve pain; harmonizes the hundred medicinals |
| Scutellariae Radix | 52 | Clear heat, dry dampness; purge fire, detoxify; stanch bleeding; prevent abortion |
| Platycodonis Radix | 38 | Diffuse the lung; soothe the throat; dispel phlegm; expel pus |
| honey-fried Glycyrrhizae Radix et Rhizoma | 32 | Tonify the spleen, harmonize the stomach; tonify qi, restore the pulse |
| Houttuyniae Herba | 24 | Clear heat, detoxify; disperse welling-abscesses, expel pus; disinhibit urine |
| Phragmitis Rhizoma | 22 | Clear heat, purge fire; engender fluid, allay thirst; eliminate vexation; check vomiting |
| Stemonae Radix | 19 | Moisten the lung, direct qi downward, suppress cough; kill worms, delousing |
| Farfarae Flos | 16 | Moisten the lung, direct qi downward; suppress cough, resolve phlegm |
| Farfarae Flos | 16 | Clear heat, stabilize fright; free the collateral vessels; calm panting; disinhibit urine |
| Trichosanthis Fructus | 15 | Clear heat, flush phlegm; loosen the chest, dissipate binds; moisten dryness, lubricate the intestines |
| Descurainiae Semen | 13 | Purge the lung, calm panting; move water, disperse swelling |
| Eriobotryae Folium | 11 | Clear lung fire, suppress cough; downbear counterflow, check vomiting |
| Mori Cortex | 11 | Purge the lung, calm panting; disinhibit water, disperse swelling |
| Pinelliae Rhizoma Praeparatum | 10 | Dry dampness, resolve phlegm |
| Gardeniae Fructus | 8 | Purge fire, eliminate vexation; clear heat, drain dampness; clear heat to cool the blood, detoxify; disperse swelling, relieve pain |
| Anemarrhenae Rhizoma | 8 | Clear heat, purge fire; enrich yin, moisten dryness |
| Persicae Semen | 8 | Promote blood circulation and dispel blood stasis; moisten the intestines, free the stool; suppress cough, calm panting |
| Coicis Semen | 7 | Disinhibit water, percolate dampness; fortify the spleen, check diarrhea; eliminate impediment; expel pus; detoxify, dissipate binds |
| Radix et Rhizoma Asteris | 6 | Moisten the lung, direct qi downward; disperse phlegm, suppress cough |
| Forsythiae Fructus | 6 | Clear heat, detoxify; disperse swelling, dissipate binds; dispelling wind and hea |
| Lonicerae Japonicae Flos | 6 | Clear heat, detoxify; dispelling wind and heat |
| Semen Benincasae | 6 | Clear lung fire, resolve phlegm; disperse welling-abscesses, expel pus; drain dampness |
| Citri Reticulatae Pericarpium | 5 | Regulate qi, fortify the spleen; dry dampness, resolve phlegm |
| Atractmlodis Macrocephalae Rhizoma | 5 | Fortify the spleen, tonify qi; dry dampness, disinhibit water; check sweating; prevent abortion |
| Peucedani Radix | 4 | Direct qi downward, resolve phlegm; dissipate wind, clear heat |
| Perillae Fructus | 4 | Direct qi downward, resolve phlegm; suppress cough, calm panting; moisten the intestines, free the stool |
| Coptidis Rhizoma | 4 | Clear heat, dry dampness; purge fire, detoxify |
| Artemisiae Annuae Herba | 4 | Clear vacuity heat; clear steaming bone; clear summerheatheat; interrupt malaria; abate jaundice |
| Pinelliae Rhizoma Praeparatum Cum Alumine | 4 | Dry dampness, resolve phlegm |
| Bupleuri Radix | 4 | Course wind and dissipate heat; soothe the liver, resolve depression; upbear yang |
| Rhei Radix et Rhizoma | 3 | Soften hardness with purgation; clear heat, purge fire; clear heat to cool the blood, detoxify; expel stasis, unblock the meridian; drain dampness, abate jaundice |
| Bambusae Caulis In Taenias | 3 | Clear heat, resolve phlegm; eliminate vexation; check vomiting |
| Fagopyri Dibotryis Rhizoma | 3 | Clear heat, detoxify; expel pus, dispel stasis |
| Phragmitis Communis Caulis | 3 | Clear heat, detoxify; disinhibit urine; expel pus |
| Jujubae Fructus | 3 | Tonify and replenish the middle qi; tonify blood, tranquilize |
| Poria | 3 | Disinhibit water, percolate dampness; fortify the spleen; quiet the heart |
| Mori Folium | 3 | Dispelling wind and heat; clear lung fire, moisten dryness; clear the liver, improve vision |
| Puerariae Lobamle Radix | 3 | Release the flesh, abate heat; engender fluid, allay thirst; outthrust rashes; upbear yang, check diarrhea; unblock the meridian, quicken the network vessels; deinebriating |
| Raphani Semen | 2 | Promote digestion, eliminate distention; direct qi downward, resolve phlegm |
| Sinapis Semen | 2 | Warm lung, clearing phlegm, regulate qi, dredge collaterals, relieve pain |
| Phellodendri Chinensis Cortex | 2 | Clear heat, dry dampness; purge fire, eliminate steam; detoxify, clove sore |
| Andrographis Herba | 2 | Clear heat, detoxify; clear heat to cool the blood; disperse swelling |
| Taraxaci Herba | 2 | Clear heat, detoxify; disperse swelling, dissipate binds; disinhibit urine, free strangury |
| Senecionis Scandentis Hebra | 2 | Clear heat, detoxify; improve vision; drain dampness |
| Saigae Tataricae Cornu | 2 | Calm the liver, extinguish wind; clear the liver, improve vision; dissipate the blood, detoxify |
| Folium Phyllostachydis Henonis | 2 | Clear heat, purge fire; eliminate vexation, allay thirst; disinhibit urine |
| Chrysanthemi Flos | 2 | Dissipate wind, clear heat, clear heat; calm the liver, improve vision; clear heat, clear heat, detoxify |
| Ardisiae Japonicae Herba | 2 | Resolve phlegm, suppress cough; clear heat and drain dampness; activate blood, transform stasis |
| Bombyx Batryticatus | 2 | Extinguish wind, check tetany; dispel wind, relieve pain; resolve phlegm, dissipate binds |
| Arecae Semen | 2 | Kill worms; disperse accumulations; move qi; disinhibit water; interrupt malaria |
| Menthae Haplocalycis Herba | 2 | Dispelling wind and heat; clear head and eyes; soothe the throat; outthrust rashes; soothe the liver, move qi |
| Arctii Fructus | 2 | Dispelling wind and heat; diffuse the lung, outthrust rashes; detoxify, soothe the throat |
| Cicadae Periostracum | 2 | Dispelling wind and heat; soothe the throat; outthrust rashes; improve vision, remove nebula and improve vision; resolve tetany |
| Bupleuri Radix processed with vinegar | 2 | Course wind and dissipate heat; soothe the liver, resolve depression; upbear yang |
| Schizonepetae Herba | 2 | Release the exterior, dissipate wind; outthrust rashes; reduce sores |
| Trichosanthis Radix | 1 | Clear heat, purge fire; engender fluid, allay thirst; disperse swelling, expel pus |
| Herba Hedyotidis | 1 | Clear heat, detoxify; activate blood, disperse swelling; drain dampness, abate jaundice |
| Polygoni Cuspidati Rhizoma | 1 | Drain dampness, abate jaundice; clear heat, detoxify; dissipate (blood) stasis, relieve pain; suppress cough, resolve phlegm |
| Liquor Phyllostachys | 1 | Clear heat, downbear fire; smooth expectoration, disinhibit the orifices |
| Arisaema Cum Bile | 1 | Clear heat, resolve phlegm; extinguish wind, stabilize fright |
| Meretricis Concha Cyclinae Concha | 1 | Clear heat, resolve phlegm; disinhibit urine; soften hardness |
| Herba Patriniae | 1 | Clear heat, drain dampness; detoxify, expel pus; activate blood, dispel stasis |
| Isatidis Radix | 1 | Clear heat, detoxify; clear heat to cool the blood, soothe the throat |
| Aurantii Fructus Immaturus | 1 | Break qi, disperse accumulations; resolve phlegm, dissipate glomus |
| stir-fried Fructus Aurantii with bran | 1 | Regulate qi, loosen the center; move stagnation, disperse distention |
| Citri Grandis Exocarpium | 1 | Regulate qi, loosen the center; dry dampness, resolve phlegm |
| Paeoniae Radix Rubra | 1 | Clear heat, cool the blood; dissipate (blood) stasis, relieve pain |
| Angelicae Sinensis Radix | 1 | Tonify blood, activate blood; regulate menstruation, relieve pain; moisten the intestines, free the stool |
| Pyrrosiae Folium | 1 | Disinhibit urine, free strangury; clear lung fire, suppress cough; clear heat to cool the blood, stanch bleeding |
| Dioscoreae Rhizoma | 1 | Nourish liver and kidney; Promote contraction, securing and astriction |
| Ginseng Radix et Rhizoma | 1 |  |
| Codonopsis Radix | 1 | Greatly tonify the original qi; restore the pulse, stem desertion; tonify the spleen, boost the lung ; engender fluid, tonify blood; tranquilize, sharpen the wits |
| Sojae Semen Praeparatum | 1 | Release the exterior; eliminate vexation; promote diffusion and effusion and dissipate evil, depressed heat |
| Belamcandae Rhizoma | 1 | Clear heat, detoxify; dispel phlegm; soothe the throat |
| Zingiberis Rhizoma Recens | 1 | Release the exterior, dissipate cold; warm the middle, check vomiting; resolve phlegm, suppress cough; resolves the toxin of fish and crabs |
| Perillae Folium | 1 | Release the exterior, dissipate cold; move qi, harmonize the stomach |
| Paeoniae Radix Alba | 1 | Tonify blood, regulate menstruation; constrain yin, check sweating; emolliate the liver, relieve pain; repress the liver |
| Schisandrae Chinensis Fructus | 1 | Promote contraction, securing and astriction; tonify qi, engender fluid; tonify the kidney, quiet the heart |
| Faeces Bombycis | 1 | Dispel wind, dispelling dampness; harmonize the stomach, transform turbidity |
| Herba Diclipterae Chinensis | 1 | Clear heat, detoxify; cool the blood; disinhibit urine |

## Supplementary Table S2. Composition of Chinese herbal formula in the 81 included studies

| **Study ID** | **Name of Chinese**  **herbal formula** | | | **Composition** | **Frequency of medication** | |  |
| --- | --- | --- | --- | --- | --- | --- | --- |
| Chen AG 2018 | | Maxing Shigan Decoction | Ephedrae Herba 9g, Armeniacae Semen Amarum 9g, Gypsum Fibrosum 24g, Glycyrrhizae Radix et Rhizoma 6g, Platycodonis Radix 10g, Scutellariae Radix 12g, Phragmitis Rhizoma 18g | | | once a day | |
| Chen ZB 2010 | | Modified Maxing Shigan Decoction | Ephedrae Herba 6g, Armeniacae Semen Amarum 9g, Gypsum Fibrosum 18g, Glycyrrhizae Radix et Rhizoma 3g, Lonicerae Japonicae Flos 15g, Andrographis Herba 9g, Taraxaci Herba 15g, Houttuyniae Herba 15g, Senecionis Scandentis Hebra 15g, Trichosanthis Pericarpium 9g, Fritillariae Cirrhosae Bulbus 6g, Pinelliae Rhizoma Praeparatum 9g | | | twice a day | |
| Cheng F 2018 | | Maxing Shigan Decoction | Ephedrae Herba 8g, Armeniacae Semen Amarum 10g, Gypsum Fibrosum 30g, honey-fried Glycyrrhizae Radix et Rhizoma 6g, Phragmitis Rhizoma 30g, Scutellariae Radix 10g, Platycodonis Radix 10g, Fritillariae Thunbergii Bulbus 10g, honey-fried Stemonae Radix 10g, Farfarae Flos 10g | | | twice a day | |
| Cheng YF 2017 | | Maxing Shigan Decoction plus Qianjin Weijing Decoction | honey-fried Ephedrae Herba 8g, Armeniacae Semen Amarum 8g, Gypsum Fibrosum 30g, Glycyrrhizae Radix et Rhizoma 6g, Trichosanthis Pericarpium 10g, Scutellariae Radix 15g, Fritillariae Thunbergii Bulbus 10g, Houttuyniae Herba 30g, Phragmitis Rhizoma 20g, Platycodonis Radix 15g, Pinelliae Rhizoma Praeparatum 10g | | | twice a day | |
| Cheng YF 2021 | | Maxing Shigan Decoction plus Qianjin Weijing Decoction | honey-fried Ephedrae Herba 10g, Armeniacae Semen Amarum 15g, Gypsum Fibrosum 30g, Glycyrrhizae Radix et Rhizoma 10g, Trichosanthis Pericarpium 15g, Persicae Semen 15g, Coicis Semen 15g, Phragmitis Communis Caulis 15g, Houttuyniae Herba 15g, Scutellariae Radix 15g | | | twice a day | |
| Chu Z 2019 | | Maxing Shigan Decoction | Ephedrae Herba 10g, Armeniacae Semen Amarum 15g, Gypsum Fibrosum 40g, Glycyrrhizae Radix et Rhizoma 6g, Atractmlodis Macrocephalae Rhizoma 10g, Scutellariae Radix 6g, Houttuyniae Herba 6g, Fritillariae Thunbergii Bulbus 4g | | | twice a day | |
| Cui CR 2022 | | Modified Maxing Shigan Decoction | honey-fried Ephedrae Herba 6g, Armeniacae Semen Amarum 9g, Gypsum Fibrosum 15g, Glycyrrhizae Radix et Rhizoma 6g, Menthae Haplocalycis Herba 10g, Perillae Folium 10g, Mori Folium 10g, Descurainiae Semen 10g, Eriobotryae Folium 10g, Trichosanthis Fructus 10g, Fritillariae Thunbergii Bulbus 10g, Scutellariae Radix 10g, Platycodonis Radix 10g | | | twice a day | |
| Dai LF 2018 | | Modified Maxing Shigan Decoction | honey-fried Ephedrae Herba 6g, Armeniacae Semen Amarum 10g, Gypsum Fibrosum 30g, honey-fried Glycyrrhizae Radix et Rhizoma 6g, Stemonae Radix 10g, Peucedani Radix 10g, Houttuyniae Herba 20g, Saigae Tataricae Cornu 0.6g | | | NR | |
| Deng SX 2018 | | Maxing Shigan Decoction | honey-fried Ephedrae Herba 10g, Stemonae Radix 10g, Farfarae Flos 10g, Scutellariae Radix 12g, Armeniacae Semen Amarum 12g, Platycodonis Radix 12g, Fritillariae Cirrhosae Bulbus 12g, Phragmitis Rhizoma 30g, Gypsum Fibrosum 30g, honey-fried Glycyrrhizae Radix et Rhizoma 6g | | | twice a day | |
| Dong LH 2023 | | Qinghua Fang | Ephedrae Herba 10g, Armeniacae Semen Amarum 9g, Gypsum Fibrosum 30g, Glycyrrhizae Radix et Rhizoma 12g, Pinelliae Rhizoma Praeparatum Cum Alumine 30g, Trichosanthis Fructus 30g, Rhei Radix et Rhizoma prepared with wine 5g, Scutellariae Radix 30g, Cicadae Periostracum 30g | | | NR | |
| Du BT 2020 | | Maxing Shigan Decoction | Ephedrae Herba 6g, Armeniacae Semen Amarum 9g, Gypsum Fibrosum 12g, honey-fried Glycyrrhizae Radix et Rhizoma 9g, Glycyrrhizae Radix et Rhizoma 3g | | | twice a day | |
| Fang F 2022 | | Modified Maxing Shigan Decoction | honey-fried Ephedrae Herba 5g, Armeniacae Semen Amarum 10g, Gypsum Fibrosum 15g, Glycyrrhizae Radix et Rhizoma 5g, Herba Diclipterae Chinensis 10g, Scutellariae Radix 10g, Fritillariae Thunbergii Bulbus 10g, Phragmitis Communis Caulis 10g, Bambusae Caulis In Taenias 10g, Mori Cortex 10g, Artemisiae Annuae Herba 10g, Faeces Bombycis 5g | | | twice a day | |
| Fei XJ 2014 | | Lung heat clearing Formula | Ephedrae Herba 3g, Armeniacae Semen Amarum 6g, Gypsum Fibrosum 15g, Glycyrrhizae Radix et Rhizoma 6g, Houttuyniae Herba 20g, Trichosanthis Fructus 9g, Scutellariae Radix 12g | | | twice a day | |
| Gao GL 2022 | | Huanglian Jiedu Decoction plus Maxing Shigan Decoction | honey-fried Ephedrae Herba 3~5g, Armeniacae Semen Amarum 6~10g, Gypsum Fibrosum 9~20g, Glycyrrhizae Radix et Rhizoma 3~5g, Scutellariae Radix 6~10g, Coptidis Rhizoma 3~6g, Gardeniae Fructus 3~10g, Phellodendri Chinensis Cortex 3~10g, Eriobotryae Folium 6~10g, Paeoniae Radix Rubra 6~10g, Persicae Semen 6~10g, Artemisiae Annuae Herba 6~10g, Fritillariae Thunbergii Bulbus 6~10g, Mori Cortex 6~10g, Scutellariae Radix 6~10g, Bambusae Caulis In Taenias 6~10g, Phragmitis Communis Caulis 6~10g | | | twice a day | |
| Guo JW 2023 | | Maxing Shigan Decoction | Ephedrae Herba 10g, Armeniacae Semen Amarum 15g, Gypsum Fibrosum 30g, honey-fried Glycyrrhizae Radix et Rhizoma 10g, Phragmitis Rhizoma 30g, Scutellariae Radix 15g, Fritillariae Thunbergii Bulbus 15g, Platycodonis Radix 15g, honey-fried Stemonae Radix 10g, Farfarae Flos 10g | | |  | |
| He XY 2011 | | Modified Maxing Shigan Decoction | honey-fried Ephedrae Herba 6g, Armeniacae Semen Amarum 10g, Gypsum Fibrosum 30g, honey-fried Glycyrrhizae Radix et Rhizoma 6g, Saigae Tataricae Cornu 0.6g, Houttuyniae Herba 20g, Peucedani Radix 10g, Stemonae Radix 10g | | | NR | |
| Hu P 2024 | | Modified Maxing Shigan Decoction | Ephedrae Herba 6g, fried Armeniacae Semen Amarum 10g, Gypsum Fibrosum 30g, Glycyrrhizae Radix et Rhizoma 3g, Scutellariae Radix processed with wine 10g, Bupleuri Radix processed with vinegar 12g, Artemisiae Annuae Herba 10g, Puerariae Lobamle Radix 15g, Arecae Semen 10g, Polygonati Odorati Rhizoma 15g | | | twice a day | |
| Hu W 2017 | | Maxing Shigan Decoction | Ephedrae Herba 6g, Armeniacae Semen Amarum 9g, Gypsum Fibrosum 12g, honey-fried Glycyrrhizae Radix et Rhizoma 9g | | | 2～3 times a day | |
| Huo HM 2016 | | Modified Maxing Shigan Decoction | Ephedrae Herba 3g, Armeniacae Semen Amarum 6g, Gypsum Fibrosum 15g, Glycyrrhizae Radix et Rhizoma 6g, Glycyrrhizae Radix et Rhizoma 15g, Astmgali Radix 15g, Stemonae Radix 6g, Fritillariae Thunbergii Bulbus 6g | | | three times a day | |
| Jin ZX 2020 | | Qingfei Mixture | Ephedrae Herba 1.66g, Armeniacae Semen Amarum 3.33g, Gypsum Fibrosum 10.00g, Glycyrrhizae Radix et Rhizoma 1.66g, Persicae Semen 3.33g, Scutellariae Radix 3.33g, Coptidis Rhizoma 0.33g, Phellodendri Chinensis Cortex 3.33g, Descurainiae Semen 3.33g, Gardeniae Fructus 3.33g | | | three times a day  (10 ml a time) | |
| Kong FH 2023 | | Maxing Shigan Decoction | Ephedrae Herba 9g, Armeniacae Semen Amarum 9g, Gypsum Fibrosum 6g, honey-fried Glycyrrhizae Radix et Rhizoma 6g | | |  | |
| Li HN 2006 | | Chinese herbal decoction | honey-fried Ephedrae Herba 8g, Armeniacae Semen Amarum 12g, Gypsum Fibrosum 20g, honey-fried Glycyrrhizae Radix et Rhizoma 6g, Pyrrosiae Folium 12g, Scutellariae Radix 10g, Houttuyniae Herba 15g, Radix et Rhizoma Asteris 15g, Platycodonis Radix 6g | | | twice a day | |
| Li JT 2020 | | Modified Maxing Shigan Decoction | Ephedrae Herba 5g, Armeniacae Semen Amarum 6g, Gypsum Fibrosum 20g, Glycyrrhizae Radix et Rhizoma 10g, Scutellariae Radix 10g, Pinelliae Rhizoma Praeparatum 5g, Aurantii Fructus Immaturus 5g, Citri Reticulatae Pericarpium 5g, Platycodonis Radix 10g | | | twice a day | |
| Li L 2021 | | Modified Maxing Shigan Decoction | Ephedrae Herba 9g, Armeniacae Semen Amarum 12g, Gypsum Fibrosum 30g, honey-fried Glycyrrhizae Radix et Rhizoma 6g, Mori Cortex 9g, Fritillariae Cirrhosae Bulbus 9g, Scutellariae Radix 9g, Poria 10g, Atractmlodis Macrocephalae Rhizoma 10g, Eriobotryae Folium 12g, Platycodonis Radix 15g | | | twice a day | |
| Li Y 2014 | | Modified Maxing Shigan Decoction | Ephedrae Herba 10g, Armeniacae Semen Amarum 12g, Gypsum Fibrosum 30g, honey-fried Glycyrrhizae Radix et Rhizoma 6g, Phragmitis Rhizoma 30g, Platycodonis Radix 12g, Fritillariae Thunbergii Bulbus 12g, Scutellariae Radix 12g, honey-fried Stemonae Radix 10g, Farfarae Flos 10g | | | twice a day | |
| Li Y 2020 | | Modified Maxing Shigan Decoction | honey-fried Ephedrae Herba 10g, Armeniacae Semen Amarum 10g, Gypsum Fibrosum 30g, honey-fried Glycyrrhizae Radix et Rhizoma 15g, Farfarae Flos 10g, Atractmlodis Macrocephalae Rhizoma 10g, Poria 10g, Fritillariae Cirrhosae Bulbus 3g | | | twice a day | |
| Li YE 2020 | | Modified Maxing Shigan Decoction | Ephedrae Herba 9g, Armeniacae Semen Amarum 6g, Gypsum Fibrosum 12g, Glycyrrhizae Radix et Rhizoma 6g, Houttuyniae Herba 12g | | | twice a day | |
| Liu BH 2018 | | Maxign Shigan Decoction plus Tingli Dazao Xiefei Decoction | Ephedrae Herba 10g, Armeniacae Semen Amarum 12g, Gypsum Fibrosum 30~50g, Glycyrrhizae Radix et Rhizoma 9g, Scutellariae Radix 15g, Liquor Phyllostachys 15g, Codonopsis Radix 20g, Ardisiae Japonicae Herba 20g, Lonicerae Japonicae Flos 20g, Pinelliae Rhizoma Praeparatum 12g, Jujubae Fructus 10g, Descurainiae Semen 10g, Houttuyniae Herba 30g, Trichosanthis Fructus 30g | | | twice a day | |
| Liu HY 2016 | | Modified Maxing Shigan Decoction | honey-fried Ephedrae Herba 10g, Armeniacae Semen Amarum 12g, Gypsum Fibrosum 30g, honey-fried Glycyrrhizae Radix et Rhizoma 6g, Stemonae Radix 10g, Fritillariae Cirrhosae Bulbus 12g, Scutellariae Radix 12g, Farfarae Flos 10g, Platycodonis Radix 12g, Phragmitis Rhizoma 30g | | | once a day | |
| Liu Y 2019 | | Maxing Shigan Decoction | Ephedrae Herba 9g, Armeniacae Semen Amarum 9g, Gypsum Fibrosum 12g, honey-fried Glycyrrhizae Radix et Rhizoma 6g, Trichosanthis Fructus 12g, Platycodonis Radix 12g, Fritillariae Thunbergii Bulbus 12g, Descurainiae Semen 9g, Scutellariae Radix 12g, Stemonae Radix 9g, Farfarae Flos 9g | | | twice a day | |
| Liu YF 2023 | | Maxing Shigan Decoction plus Qianjin Weijing Decoction | honey-fried Ephedrae Herba 6g, Armeniacae Semen Amarum 9g, Gypsum Fibrosum 20g, Glycyrrhizae Radix et Rhizoma 6g, Phragmitis Rhizoma 30g, Coicis Semen 30g, Semen Benincasae 30g, Persicae Semen 10g | | | twice a day | |
| Liu YH 2016 | | Modified Maxing Shigan Decoction | Ephedrae Herba 6g, Armeniacae Semen Amarum 15g, Gypsum Fibrosum 15g, honey-fried Glycyrrhizae Radix et Rhizoma 6g, Mori Cortex 9g, Descurainiae Semen 12g, Eriobotryae Folium 12g | | | three times a day | |
| Liu YR 2011 | | Modified Maxing Shigan Decoction | honey-fried Ephedrae Herba 10g, Gypsum Fibrosum 20g, Armeniacae Semen Amarum 10g, Glycyrrhizae Radix et Rhizoma 10g, Platycodonis Radix 10g, Houttuyniae Herba 20g | | | twice a day | |
| Liu YR 2019 | | Modified Maxing Shigan Decoction | honey-fried Ephedrae Herba 10g, Armeniacae Semen Amarum 10g, Gypsum Fibrosum 20g, Glycyrrhizae Radix et Rhizoma 10g, Platycodonis Radix 10g, Houttuyniae Herba 20g | | | twice a day | |
| Lu TF 2010 | | Yinqiao San plus Maxing Shigan Decoction | honey-fried Ephedrae Herba 10g, Armeniacae Semen Amarum 10g, Gypsum Fibrosum 30g, honey-fried Glycyrrhizae Radix et Rhizoma 6g, Lonicerae Japonicae Flos 15g, Forsythiae Fructus 15g, Folium Phyllostachydis Henonisl 10g, Menthae Haplocalycis Herba 12g, Trichosanthis Fructus 15g, Mori Cortex 10g, Platycodonis Radix 10g, Schizonepetae Herba 15g, Radix et Rhizoma Asteris 15g, Stemonae Radix 15g, Citri Reticulatae Pericarpium 12g | | | NR | |
| Ma FM 2020 | | Maxing Shigan Decoction plus Tingli Dazao Xiefei Decoction | Ephedrae Herba 9g, Armeniacae Semen Amarum 9g, Gypsum Fibrosum 24g, Glycyrrhizae Radix et Rhizoma 6g, Descurainiae Semen 10g, Jujubae Fructus 10g | | | twice a day | |
| Ma WJ 2015 | | Xianma Xiaotan Decoction | honey-fried Ephedrae Herba 10g, Armeniacae Semen Amarum 10g, Gypsum Fibrosum 20g, Glycyrrhizae Radix et Rhizoma 5g, Trichosanthis Fructus 20g, Coptidis Rhizoma 6g, Pinelliae Rhizoma Praeparatum 15g, Fritillariae Thunbergii Bulbus 15g | | | three times a day | |
| Ma XY 2011 | | Modified Maxing Shigan Decoction | Ephedrae Herba 10g, Armeniacae Semen Amarum 12g, Gypsum Fibrosum 30g, honey-fried Glycyrrhizae Radix et Rhizoma 6g, Phragmitis Rhizoma 30g, Platycodonis Radix 12g, Fritillariae Thunbergii Bulbus 12g, Scutellariae Radix 12g, Galli Gigerii Endothelium Corneum 10g, honey-fried Stemonae Radix 10g, Farfarae Flos 10g | | | NR | |
| Ma YF 2017 | | Modified Maxing Shigan Decoction | honey-fried Ephedrae Herba 5g, Gypsum Fibrosum 10g, Armeniacae Semen Amarum 6g, honey-fried Glycyrrhizae Radix et Rhizoma 5g, Lonicerae Japonicae Flos 10g, Scutellariae Radix 6g, Pinelliae Rhizoma Praeparatum Cum Alumine 6g, Trichosanthis Pericarpium 8g | | | three times a day | |
| Meng W 2017 | | Modified Maxing Shigan Decoction | Ephedrae Herba 20g, Armeniacae Semen Amarum 12g, Gypsum Fibrosum 40g, honey-fried Glycyrrhizae Radix et Rhizoma 15g, Fritillariae Cirrhosae Bulbus 20g, Houttuyniae Herba 20g, Descurainiae Semen 15g, Anemarrhenae Rhizoma 15g, Scutellariae Radix 15g, Platycodonis Radix 20g | | | twice a day | |
| Mo WS 2021 | | Maxing Shigan decoction plus Qianjin Weijing decoction | Ephedrae Herba 5g, Armeniacae Semen Amarum 10g, Gypsum Fibrosum 30g, Persicae Semen 10g, Glycyrrhizae Radix et Rhizoma 3g, Eriobotryae Folium 10g, Scutellariae Radix 10g, Platycodonis Radix 10g, Fritillariae Thunbergii Bulbus 10g, Semen Benincasae 10g, Phragmitis Rhizoma 15g, Coicis Semen 25g | | | twice a day | |
| Mo ZH 2022 | | Feirening Mixture | honey-fried Ephedrae Herba 3.75g, Armeniacae Semen Amarum 2.5g, Paeoniae Radix Alba 3.75g, Stemonae Radix 2.5g, honey-fried Radix et Rhizoma Asteris 2.5g, Platycodonis Radix 2.5g, Fritillariae Ussuriensis Bulbus 3.75g, honey-fried Eriobotryae Folium 1.25g, Scutellariae Radix 3.75g, Gypsum Fibrosum 3.75g, Mori Cortex 3.75g, Anemarrhenae Rhizoma 2.5g, Trichosanthis Fructus 2.5g, Bambusae Caulis In Taenias 2.5g, Houttuyniae Herba 3.75g, Atractmlodis Macrocephalae Rhizoma 2.5g, stir-fried Fructus Aurantii with bran 2.5g, Galli Gigerii Endothelium Corneum 2.5g, Coicis Semen 3.75g, honey-fried Glycyrrhizae Radix et Rhizoma 2.5g, Arctii Fructus 3.75g, Phragmitis Rhizoma 5g, Angelicae Sinensis Radix 3.75g, Ardisiae Japonicae Herba 2.5g, Dioscoreae Rhizoma 2.5g | | | three times a day | |
| Ni CY 2023 | | Modified Maxing Shigan Decoction | Ephedrae Herba 9g, Armeniacae Semen Amarum 9g, Gypsum Fibrosum 18g, Glycyrrhizae Radix et Rhizoma 6g, Fritillariae Cirrhosae Bulbus 10g, Platycodonis Radix 10g, Anemarrhenae Rhizoma 10g, Scutellariae Radix 15g, Houttuyniae Herba 15g, Descurainiae Semen 15g | | | twice a day | |
| Ning WJ 2022 | | Xiaochaihu Decoction plus Maxing Shigan Decoction | honey-fried Ephedrae Herba 5 g~10 g, Armeniacae Semen Amarum 10g, Gypsum Fibrosum 30g~50g, honey-fried Glycyrrhizae Radix et Rhizoma 10g, 4 Jujubae Fructus, Zingiberis Rhizoma Recens 9g, Pinelliae Rhizoma Praeparatum Cum Alumine 9g, Ginseng Radix et Rhizoma 9g, Scutellariae Radix 9g, Bupleuri Radix 15g | | | twice a day | |
| Shen AY 2014 | | Modified Maxing Shigan Decoction | honey-fried Ephedrae Herba 10g, Armeniacae Semen Amarum 12g, Gypsum Fibrosum 30g, Scutellariae Radix 12g, Phragmitis Rhizoma 30g, Platycodonis Radix 12g, Farfarae Flos 10g, Fritillariae Cirrhosae Bulbus 12g, Stemonae Radix 10g, honey-fried Glycyrrhizae Radix et Rhizoma 6g | | | twice a day | |
| Shen RX 2013 | | Modified Maxing Shigan Decoction | Ephedrae Herba 6g, Armeniacae Semen Amarum 12g, Gypsum Fibrosum 30g, Glycyrrhizae Radix et Rhizoma 6g, Farfarae Flos 12g, Radix et Rhizoma Asteris 12g, honey-fried Stemonae Radix 12g, Fritillariae Thunbergii Bulbus 15g, Scutellariae Radix 10g | | | twice a day | |
| Shi H 2010 | | Pneumonia Mixture (hospital preparation) | Ephedrae Herba, Armeniacae Semen Amarum, Gypsum Fibrosum, Glycyrrhizae Radix et Rhizoma, Fritillariae Thunbergii Bulbus, Houttuyniae Herba, Herba Patriniae | | | three times a day  (80 ml a time) | |
| Song DF 2022 | | Maxing Shigan Decoction | Ephedrae Herba 4g, Armeniacae Semen Amarum 5g, Gypsum Fibrosum 12g, honey-fried Glycyrrhizae Radix et Rhizoma 4g, Lonicerae Japonicae Flos 9g, Scutellariae Radix 6g, Pinelliae Rhizoma Praeparatum Cum Alumine 6g, Trichosanthis Pericarpium 8g | | | twice a day | |
| Su QZ 2016 | | Modified Maxing Shigan Decoction | Ephedrae Herba 20g, Armeniacae Semen Amarum 25g, Gypsum Fibrosum 40g, honey-fried Glycyrrhizae Radix et Rhizoma 20g, Arctii Fructus 20g, Descurainiae Semen 20g, Mori Folium 25g, Chrysanthemi Flos 30g, Anemarrhenae Rhizoma 15g, Fritillariae Cirrhosae Bulbus 20g, Houttuyniae Herba 15g, Isatidis Radix 20g | | | twice a day | |
| Su SD 2016 | | Maxing Shigan Decoction | Ephedrae Herba 9g, Armeniacae Semen Amarum 9g, Gypsum Fibrosum 20g, Glycyrrhizae Radix et Rhizoma 6g | | | three times a day | |
| Sun K 2022 | | Maxing Shigan Decoction | honey-fried Ephedrae Herba 10g, Armeniacae Semen Amarum 12g, honey-fried Glycyrrhizae Radix et Rhizoma 6g, Gypsum Fibrosum 30g, Scutellariae Radix 12g, Phragmitis Rhizoma 30g, Farfarae Flos 10g, Fritillariae Cirrhosae Bulbus 12g, Fagopyri Dibotryis Rhizoma 20g, Eriobotryae Folium 15g | | | twice a day | |
| Sun QQ 2019 | | Maxing Shigan Decoction | Ephedrae Herba 9g, Armeniacae Semen Amarum 12g, Gypsum Fibrosum 30g, honey-fried Glycyrrhizae Radix et Rhizoma 6g, Platycodonis Radix 15g, Eriobotryae Folium 12g, Atractmlodis Macrocephalae Rhizoma 10g, Poria 10g, Fritillariae Cirrhosae Bulbus 9g, Scutellariae Radix 9g, Mori Cortex 9g | | | twice a day | |
| Sun QS 2015 | | Modified Maxing Shigan Decoction | honey-fried Ephedrae Herba 10g, Armeniacae Semen Amarum 10g, Gypsum Fibrosum 15g, Glycyrrhizae Radix et Rhizoma 10g, Scutellariae Radix 10g, Fritillariae Cirrhosae Bulbus 10g, Platycodonis Radix 10g, Phragmitis Rhizoma 10g, Pinelliae Rhizoma 10g, Stemonae Radix 10g, Gardeniae Fructus 10g, Trichosanthis Fructus 5g, Perillae Fructus 5g, Raphani Semen 5g, Sinapis Semen 5g, Trichosanthis Radix 5g | | | twice a day | |
| Tang H 2013 | | Modified Maxing Shigan Decoction | honey-fried Ephedrae Herba 9g, Armeniacae Semen Amarum10g, Gypsum Fibrosum 18g, Glycyrrhizae Radix et Rhizoma 6g, Scutellariae Radix 10g, Lonicerae Japonicae Flos10g, Houttuyniae Herba 10g, Mori Cortex 10g, Gardeniae Fructus 10g, Pinelliae Rhizoma 10g, Rhei Radix et Rhizoma 6g | | | twice a day | |
| Tian XM 2017 | | Maxing Shigan Decoction | Ephedrae Herba 8g, Armeniacae Semen Amarum 10g, Gypsum Fibrosum 30g, honey-fried Glycyrrhizae Radix et Rhizoma 6g, Scutellariae Radix 10g, Platycodonis Radix 10g, Phragmitis Rhizoma 30g, Fritillariae Thunbergii Bulbus 10g, honey-fried Stemonae Radix 10g, Farfarae Flos 10g | | | twice a day | |
| Tian YH 2013 | | Modified Maxing Shigan Decoction | Ephedrae Herba 5g, Armeniacae Semen Amarum 9g, Glycyrrhizae Radix et Rhizoma 6g, Gypsum Fibrosum 18 g, Mori Folium 12g, Chrysanthemi Flos 15g, Schizonepetae Herba 10g, Sojae Semen Praeparatum 12g, Anemarrhenae Rhizoma 10g, Lonicerae Japonicae Flos 12 g, Forsythiae Fructus 12g, Platycodonis Radix 12g | | | twice a day | |
| Wang CH 2015 | | Maxing Shigan Decoction | Ephedrae Herba 6g, Armeniacae Semen Amarum 15g, Gypsum Fibrosum 15g, honey-fried Glycyrrhizae Radix et Rhizoma 6g, Fritillariae Cirrhosae Bulbus 9g, Fagopyri Dibotryis Rhizoma 20g, Eriobotryae Folium 15g | | | twice a day | |
| Wang CM 2014 | | Modified Maxing Shigan Decoction | honey-fried Ephedrae Herba 6g, Armeniacae Semen Amarum 12g, Gypsum Fibrosum 30g, Glycyrrhizae Radix et Rhizoma 10g, Bupleuri Radix 20g, Scutellariae Radix 15g, Forsythiae Fructus 15g, Phragmitis Rhizoma 15g, Platycodonis Radix 10g | | | twice a day | |
| Wang JH 2020 | | Maxing Shigan Decoction | Ephedrae Herba 6~9g, Armeniacae Semen Amarum 6g、Gypsum Fibrosum 9~12g, Glycyrrhizae Radix et Rhizoma 3~6g, Houttuyniae Herba 9~12g | | | twice a day | |
| Wang JJ 2017 | | Modified Maxing Shigan Decoction | Gypsum Fibrosum 30g, honey-fried Glycyrrhizae Radix et Rhizoma 6g, Ephedrae Herba 8g, Phragmitis Rhizoma 30g, Armeniacae Semen Amarum 10g, Platycodonis Radix 10g, Scutellariae Radix 10g, honey-fried Stemonae Radix 10g, Fritillariae Thunbergii Bulbus 10g, Farfarae Flos 10g | | | twice a day | |
| Wang JT 2014 | | Tanre Kesou Fang | Ephedrae Herba 6g, Armeniacae Semen Amarum 6g, Gypsum Fibrosum 30g, Glycyrrhizae Radix et Rhizoma 3g, Radix et Rhizoma Asteris 10g, Stemonae Radix 10g, Eriobotryae Folium 10g, Fritillariae Thunbergii Bulbus 15g, Trichosanthis Pericarpium 10g, Belamcandae Rhizoma 10g，Bombyx Batryticatus10g | | | 2～3 times a day | |
| Wang XM 2024 | | Maxing Shigan Decoction plus Qianjin Weijing Decoction | Ephedrae Herba 6g, Armeniacae Semen Amarum 9g, Gypsum Fibrosum 30g, Glycyrrhizae Radix et Rhizoma 6g, Semen Benincasae 30g, Phragmitis Rhizoma 30g, Coicis Semen 30g, Persicae Semen 9g | | | once a day | |
| Wu TF 2013 | | Modified Maxing Shigan Decoction | honey-fried Ephedrae Herba 2~5g, Armeniacae Semen Amarum 6~10g, Gypsum Fibrosum 5~10g, Glycyrrhizae Radix et Rhizoma 3~5g, Trichosanthis Pericarpium 10~15g, Phragmitis Rhizoma 6~10g, Fritillariae Thunbergii Bulbus 5~10g, Farfarae Flos 6~10g | | | once a day | |
| Wu XK 2016 | | Detoxification-Clearing Lung Mixture (hospital preparation) | honey-fried Ephedrae Herba, Armeniacae Semen Amarum, Gypsum Fibrosum, Glycyrrhizae Radix et Rhizoma，Scutellariae Radix, Polygoni Cuspidati Rhizoma、Forsythiae Fructus, Bombyx Batryticatus, Arctii Fructus、Platycodonis Radix, Herba Hedyotidis, Trichosanthis Fructus | | | three times a day  (35 ml a time) | |
| Xiao YL 2021 | | Yinhuang Qingfei Decoction | Ephedrae Herba 8g, Armeniacae Semen Amarum 15g, Gypsum Fibrosum 30g, Glycyrrhizae Radix et Rhizoma 6g, Lonicerae Japonicae Flos 30g, Scutellariae Radix 30g, Forsythiae Fructus 15g, Houttuyniae Herba 30g, Semen Benincasae 30g, Polygalae Radix 10g, Platycodonis Radix 15g, Arisaema Cum Bile 30g, Farfarae Flos 15g, Descurainiae Semen 15g | | | three times a day | |
| Xie JJ 2010 | | Self Maxing Mixture | honey-fried Ephedrae Herba 3~8g, Armeniacae Semen Amarum 6~10g, Gypsum Fibrosum 3~12g, Glycyrrhizae Radix et Rhizoma 3~6g, Platycodonis Radix 3~10g, Peucedani Radix 6~10g, Pinelliae Rhizoma Praeparatum 6~10g, Trichosanthis Pericarpium 6~10g, Descurainiae Semen 3~8g, Meretricis Concha Cyclinae Concha 10~15g, Radix et Rhizoma Asteris 6~10g | | | twice a day | |
| Xie Y 2023 | | Qianjin Weijing Decoction plus Maxing Shigan Decoction | Ephedrae Herba 6g, Armeniacae Semen Amarum 9g, Gypsum Fibrosum 15g, Glycyrrhizae Radix et Rhizoma 6g, Phragmitis Rhizoma 40g, Semen Benincasae 15g, Coicis Semen 20g, Persicae Semen 15g, Rhei Radix et Rhizoma 6g, Moutan Cortex12g, Scutellariae Radix 12g, Astmgali Radix 20g | | | twice a day | |
| Xin DY 2018 | | Maxing Shigan Decoction plus Gegen Qinlian Decoction | Ephedrae Herba 6g, Armeniacae Semen Amarum 6g, Gypsum Fibrosum 20g, honey-fried Glycyrrhizae Radix et Rhizoma 5g, Puerariae Lobamle Radix 15g, Scutellariae Radix 9g, Coptidis Rhizoma 6g | | | twice a day | |
| Xu GL 2016 | | Maxing Shigan Decoction plus Qianjin Weijing Decoction | Ephedrae Herba 5g, Armeniacae Semen Amarum 10g, Gypsum Fibrosum 30g, Glycyrrhizae Radix et Rhizoma 3g, Phragmitis Rhizoma 15g, Persicae Semen 10g, Coicis Semen 25g, Semen Benincasae 10g, Scutellariae Radix 10 g, Fritillariae Thunbergii Bulbus 10g, Platycodonis Radix 10 g, Eriobotryae Folium 10g | | | twice a day | |
| Xu GL 2020 | | Modified Maxing Shigan Decoction | Ephedrae Herba 6g, Armeniacae Semen Amarum 10g, Gypsum Fibrosum 30g, Glycyrrhizae Radix et Rhizoma 3g, Forsythiae Fructus 10g, Scutellariae Radix 10g, Platycodonis Radix 10g, Citri Reticulatae Pericarpium 10 g, Fritillariae Thunbergii Bulbus 10g, Houttuyniae Herba 10 g | | | twice a day | |
| Yang HG 2014 | | Modified Maxing Shigan Decoction | Ephedrae Herba 15g, Armeniacae Semen Amarum 10g, Gypsum Fibrosum 60g, Glycyrrhizae Radix et Rhizoma 6g, Perillae Fructus 10g, Fagopyri Dibotryis Rhizoma 10g, Farfarae Flos 10g, Cicadae Periostracum 10g, Schisandrae Chinensis Fructus 10g, Platycodonis Radix 15g | | | twice a day | |
| Yang J 2020 | | Modified Maxing Shigan Decoction | Ephedrae Herba 20g, Armeniacae Semen Amarum 12g, Gypsum Fibrosum 40g, honey-fried Glycyrrhizae Radix et Rhizoma 15g, Fritillariae Cirrhosae Bulbus 20g, Houttuyniae Herba 20g, Platycodonis Radix 20g, Descurainiae Semen 15g, Scutellariae Radix 15g, Anemarrhenae Rhizoma 15g | | | twice a day | |
| Yang JS 2021 | | Modified Maxing Shigan Decoction | Ephedrae Herba 10g, Armeniacae Semen Amarum 10g, Gypsum Fibrosum 20g, honey-fried Glycyrrhizae Radix et Rhizoma 6g, Trichosanthis Fructus 20g, Mori Cortex 12g, Scutellariae Radix 9g, Folium Phyllostachydis Henonis 10g | | | twice a day | |
| Yuan LL 2008 | | Modified Maxing Shigan Decoction | Ephedrae Herba 6g, Armeniacae Semen Amarum 9g, Gypsum Fibrosum 30g, Glycyrrhizae Radix et Rhizoma 3g, Fritillariae Cirrhosae Bulbus 6g, Trichosanthis Fructus 30g, Taraxaci Herba 30g, Lonicerae Japonicae Flos 15g, Houttuyniae Herba 15g, Senecionis Scandentis Hebra 15g, Andrographis Herba 15g, Pinelliae Rhizoma 3g | | | twice a day | |
| Zhang JJ 2020 | | Modified Maxing Shigan Decoction | honey-fried Ephedrae Herba 9g, Armeniacae Semen Amarum 12g, Gypsum Fibrosum 30g, Glycyrrhizae Radix et Rhizoma 6g, Mori Cortex 9g, Scutellariae Radix 9g, Fritillariae Thunbergii Bulbus 9g, Platycodonis Radix 6g, Citri Reticulatae Pericarpium 6g, Trichosanthis Fructus 12g, Gardeniae Fructus 9g, Anemarrhenae Rhizoma 9g | | | twice a day | |
| Zhang L 2019 | | Bawei Qingfei Decoction | honey-fried Ephedrae Herba 5g, Armeniacae Semen Amarum 6g, Gypsum Fibrosum 18g, Glycyrrhizae Radix et Rhizoma 4g, Descurainiae Semen 4g, Houttuyniae Herba 12g, Scutellariae Radix 9g, Perillae Fructus 6g | | | three times a day | |
| Zheng YJ 2022 | | Maxing Shigan Decoction | Ephedrae Herba 6g, Armeniacae Semen Amarum 6g, Gypsum Fibrosum 24g, Glycyrrhizae Radix et Rhizoma 6g | | | three times a day | |
| Zhou DH 2016 | | Modified Maxing Shigan Decoction | honey-fried Ephedrae Herba 9g, Armeniacae Semen Amarum 12g, Gypsum Fibrosum 30g, Glycyrrhizae Radix et Rhizoma 6g, Mori Cortex 9g, Scutellariae Radix 9g, Fritillariae Thunbergii Bulbus 9g, Platycodonis Radix 6g, Citri Reticulatae Pericarpium 6g, Gardeniae Fructus 9g, Trichosanthis Fructus 12g, Anemarrhenae Rhizoma 9g | | | twice a day | |
| Zhou YH 2015 | | Modified Maxing Shigan Decoction | honey-fried Ephedrae Herba 10g, Armeniacae Semen Amarum 10g, Gypsum Fibrosum 30g, Glycyrrhizae Radix et Rhizoma 10g, Platycodonis Radix 10g, Scutellariae Radix 10g, Gardeniae Fructus 10g, Stemonae Radix 10g, Sinapis Semen 6g, Raphani Semen 10g, Perillae Fructus 10g, Pinelliae Rhizoma 10g, Citri Grandis Exocarpium 10g, Phragmitis Rhizoma 30g, Fritillariae Thunbergii Bulbus 10g | | | twice a day | |
| Zhu J 2022 | | Modified Maxing Shigan Decoction | Ephedrae Herba 6g, Armeniacae Semen Amarum 10g, Radix et Rhizoma Asteris 30g, Glycyrrhizae Radix et Rhizoma 3g, Scutellariae Radix processed with wine10g, Bupleuri Radix processed with vinegar 12g, Artemisiae Annuae Herba 10g, Puerariae Lobamle Radix 15g, Arecae Semen 10g, Polygonati Odorati Rhizoma 15g | | | twice a day | |
| Zou P 2018 | | Modified Maxing Shigan Decoction | honey-fried Ephedrae Herba 10g, Gypsum Fibrosum 30g, Armeniacae Semen Amarum 15g, Glycyrrhizae Radix et Rhizoma 6g, Scutellariae Radix 15g, Gardeniae Fructus 10g, Trichosanthis Fructus 15g, Peucedani Radix 15g, Platycodonis Radix 15g | | | twice a day | |

**ml**, milliliter

| Supplementary Table S3. Details of subgroup analyses (MXSG+WM vs WM) | | | | | | | | | | |
| --- | --- | --- | --- | --- | --- | --- | --- | --- | --- | --- |
| **Outcome** | **Type of subgroup** | **Study number** | | **Effect size (95% CI)** | | **Heterogeneity** | | **Interaction P value** | | |
| Resolution time of fever | Syndrome differentiation and treatment | | | | | | | | | |
|  | With | 17 | | MD=-1.58 [-2.00, -1.16] | | P<0.00001, I^2^=96% | | | P=0.97 | |
|  | Without | 11 | | MD=-1.59 [-2.00, -1.18] | | P<0.00001, I^2^=97% | | |  | |
|  | Flavored quantity of Chinese medicine | | | | | | | | | |
|  | >0, ≤4 | 10 | | MD=-1.25 [-1.48, -1.03] | | P<0.00001, I^2^=88% | | | P=0.14 | |
|  | >4, <8 | 13 | | MD=-1.62 [-2.05, -1.18] | | P<0.00001, I^2^=95% | | |  | |
|  | ≥8 | 5 | | MD=-1.98 [-2.89, -1.07] | | P<0.00001, I^2^=98% | | |  | |
|  | Age |  | |  | |  | | |  | |
|  | Elders | 2 | | MD= -3.12 [-3.28, -2.96] | | P=0.44, I^2^=0% | | | **P<0.00001** | |
|  | Other age groups | 26 | | MD= -1.45 [-1.68, -1.22] | | P<0.00001, I^2^=94% | | |  | |
| Resolution time of cough | Syndrome differentiation and treatment | | | | | | | | | |
|  | With | 15 | | MD=-2.52 [-2.89, -2.14] | | P<0.00001, I^2^=80% | | | P=0.09 | |
|  | Without | 10 | | MD=-2.00 [-2.46, -1.54] | | P<0.00001, I^2^=88% | | |  | |
|  | Flavored quantity of Chinese medicine | | | | | | | | | |
|  | >0, ≤4 | 9 | | MD=-2.36 [-3.04, -1.69] | | P<0.00001, I^2^=92% | | | P=0.73 | |
|  | >4, <8 | 12 | | MD=-2.32 [-2.58, -2.06] | | P=0.03, I^2^=49% | | |  | |
|  | ≥8 | 4 | | MD=-2.46 [-2.69, -2.23] | | P=0.72, I^2^=0% | | |  | |
|  | Age |  | |  | |  | | |  | |
|  | Children | 8 | | MD=-2.37 [-3.13, -1.60] | | P<0.00001, I^2^=94% | | | P=0.33 | |
|  | Adults | 3 | | MD=-2.74 [-3.52, -1.96] | | P=0.08, I^2^=60% | | |  | |
|  | Elders | 2 | | MD=-2.44 [-2.72, -2.16] | | P=0.59, I^2^=0% | | |  | |
|  | Adults+Children | 1 | | MD=-2.56 [-2.90, -2.22] | | Not available | | |  | |
|  | Adults+Elders | 11 | | MD=-2.18 [-2.43, -1.93] | | P=0.13, I^2^=34% | | |  | |
| Resolution time of pulmonary crepitation | Syndrome differentiation and treatment | | | | | | | | | |
|  | With | 13 | | MD=-2.22 [-2.65, -1.78] | | P<0.00001, I^2^=86% | | | P=0.48 | |
|  | Without | 10 | | MD=-1.99 [-2.44, -1.54] | | P<0.00001, I^2^=86% | | |  | |
|  | Flavored quantity of Chinese medicine | | | | | | | | | |
|  | >0, ≤4 | 9 | | MD=-1.93 [-2.45, -1.40] | | P<0.00001, I^2^=90% | | | P<0.00001 | |
|  | >4, <8 | 11 | | MD=-1.95 [-2.35, -1.54] | | P<0.00001, I^2^=79% | | |  | |
|  | ≥8 | 3 | | MD=-3.26 [-3.63, -2.89] | | P=0.74, I^2^=0% | | |  | |
|  | Age |  | |  | |  | | |  | |
|  | Eleders | 3 | | MD=-3.41 [-3.89, -2.94] | | P=0.22, I^2^=33% | | | **P<0.00001** | |
|  | Other age groups | 20 | | MD=-1.88 [-2.18, -1.59] | | P<0.00001, I^2^=83% | | |  | |
| CRP | Syndrome differentiation and treatment | | | | | | | | | |
|  | With | 16 | | MD=-1.99 [-2.65, -1.32] | | P<0.00001, I^2^=96% | | | P=0.13 | |
|  | Without | 22 | | MD=-2.71 [-3.37, -2.05] | | P<0.00001, I^2^=97% | | |  | |
|  | Flavored quantity of Chinese medicine | | | | | | | | | |
|  | =0 | 2 | | MD=-5.32 [-8.55, -2.09] | | P<0.00001, I^2^=98% | | | P<0.00001 | |
|  | >0, ≤4 | 14 | | MD=-2.25 [-2.99, -1.50] | | P<0.00001, I^2^=96% | | |  | |
|  | >4, <8 | 15 | | MD=-2.84 [-3.63, -2.06] | | P<0.00001, I^2^=96% | | |  | |
|  | ≥8 | 7 | | MD=-1.01 [-1.53, -0.49] | | P<0.00001, I^2^=88% | | |  | |
|  | Age |  | |  | |  | | |  | |
|  | Children | 6 | | MD=-3.10 [-4.78, -1.41] | | P<0.00001, I^2^=98% | | | P=0.73 | |
|  | Adults | 13 | | MD=-2.16 [-2.82, -1.50] | | P<0.00001, I^2^=95% | | |  | |
|  | Elders | 6 | | MD=-2.75 [-4.23, -1.27] | | P<0.00001, I^2^=97% | | |  | |
|  | Adults+Children | 1 | | MD=-2.41 [-2.91, -1.91] | | Not available | | |  | |
|  | Adults+Elders | 12 | | MD=-2.04 [-2.80, -1.28] | | P<0.00001, I^2^=96% | | |  | |
| WBC | Syndrome differentiation and treatment | | | | | | | | | |
|  | With | 14 | | MD=-1.13 [-1.59, -0.67] | | P<0.00001, I^2^=91% | | | P=0.34 | |
|  | Without | 19 | | MD=-0.83 [-1.25, -0.40] | | P<0.00001, I^2^=92% | | |  | |
|  | Flavored quantity of Chinese medicine | | | | | | | | | |
|  | >0, ≤4 | 10 | | MD=-1.60 [-2.53, -0.67] | | P<0.00001, I^2^=96% | | | P=0.22 | |
|  | >4, <8 | 15 | | MD=-0.75 [-1.06, -0.43] | | P<0.00001, I^2^=85% | | |  | |
|  | ≥8 | 8 | | MD=-0.91 [-1.25, -0.57] | | P=0.0003, I^2^=74% | | |  | |
|  | Age | | | | | | | | | |
|  | Children | 1 | | MD=-5.12 [-6.06, -4.19] | | Not available | | | P<0.00001 | |
|  | Adults | 11 | | MD=-0.92 [-1.20, -0.64] | | P<0.00001, I^2^=71% | | |  | |
|  | Elders | 8 | | MD=-0.58 [-1.22, 0.06] | | P<0.00001, I^2^=92% | | |  | |
|  | Adults+Elders | 13 | | MD=-1.01 [-1.55, -0.46] | | P<0.00001, I^2^=93% | | |  | |
|  | Severity of CAP | | | | | | | | | |
|  | Severe CAP | 4 | | MD=-1.02 [-1.63, -0.41] | | P=0.005, I^2^=76% | | | P=0.2 | |
|  | None-severe CAP | 6 | | MD=-0.63 [-0.96, -0.29] | | P=0.01, I^2^=62% | | |  | |
|  | CAP | 24 | | MD=-1.09 [-1.51, -0.67] | | P<0.00001, I^2^=94% | | |  | |
| PCT | With | 18 | | MD=-2.18 [-2.77, -1.59] | | P<0.00001, I^2^=96% | | | P=0.84 | |
|  | Without | 10 | | MD=-2.28 [-3.08, -1.48] | | P<0.00001, I^2^=95% | | |  | |
|  | Flavored quantity of Chinese medicine | | | | | | | | | |
|  | =0 | 2 | | MD=-3.38 [-6.63, -0.12] | | P<0.00001, I^2^=99% | | | P=0.16 | |
|  | >0, ≤4 | 7 | | MD=-1.80 [-2.65, -0.94] | | P<0.00001, I^2^=93% | | |  | |
|  | >4, <8 | 15 | | MD=-1.77 [-2.26, -1.28] | | P<0.00001, I^2^=92% | | |  | |
|  | ≥8 | 4 | | MD=-4.71 [-7.45, -1.96] | | P<0.00001, I^2^=98% | | |  | |
|  | Age | | | | | | | | | |
|  | Children | 3 | | MD=-2.53 [-4.29, -0.77] | | P<0.00001, I^2^=98% | | | P<0.00001 | |
|  | Adults | 6 | | MD=-2.62 [-3.63, -1.61] | | P<0.00001, I^2^=92% | | |  | |
|  | Elders | 6 | | MD=-3.49 [-5.26, -1.72] | | P<0.00001, I^2^=98% | | |  | |
|  | Adults+Children | 1 | | MD=-0.80 [-1.19, -0.41] | | Not available | | |  | |
|  | Adults+Elders | 12 | | MD=-1.62 [-2.12, -1.12] | | P<0.00001, I^2^=91% | | |  | |
|  | Severity of CAP | | | | | | | | | |
|  | Severe CAP | 3 | | MD=-1.76 [-2.38, -1.13] | | P=0.03, I^2^=72% | | | P=0.38 | |
|  | None-severe CAP | 2 | | MD=-1.75 [-3.66, 0.16] | | P<0.00001, I^2^=95% | | |  | |
|  | CAP | 23 | | MD=-2.33 [-2.88, -1.78] | | P<0.00001, I^2^=96% | | |  | |
| Absorption Time of Lung Inflammation | Syndrome differentiation and treatment | | | | | | | | | |
|  | With | 7 | | MD=-3.01 [-4.03, -1.99] | | P=0.0004, I^2^=76% | | | P=0.59 | |
|  | Without | 1 | | MD=-3.40 [-4.44, -2.37] | | Not available | | |  | |
|  | Flavored quantity of Chinese medicine | | | | | | | | | |
|  | <8 | 6 | | MD=-2.71 [-3.42, -1.99] | | P=0.08, I^2^=49% | | | **P=0.0008** | |
|  | ≥8 | 2 | | MD=-4.75 [-5.70, -3.79] | | P=0.94, I^2^=0% | | |  | |
|  | Age | | | | | | | | | |
|  | Children | 3 | | MD=-2.44 [-3.47, -1.41] | | P=0.03, I^2^=72% | | | P=0.09 | |
|  | Elders | 3 | | MD=-4.09 [-5.33, -2.86] | | P=0.05, I^2^=67% | | |  | |
|  | Adults+Elders | 2 | | MD=-4.26 [-6.76, -1.77] | | P=0.46, I^2^=0% | | |  | |
| Improvement Rate of Chest Radiograph | Syndrome differentiation and treatment | | | | | | | | | |
|  | With | 16 | | RR=1.19 [1.11, 1.28] | | P<0.00001, I^2^=73% | | | P=0.23 | |
|  | Without | 26 | | RR=1.13 [1.08, 1.19] | | P<0.00001, I^2^=71% | | |  | |
|  | Flavored quantity of Chinese medicine | | | | | | | | | |
|  | =0 | 2 | | RR=1.11 [0.80, 1.54] | | P=0.03, I^2^=80% | | | P=0.66 | |
|  | >0, ≤4 | 10 | | RR=1.17 [1.11, 1.23] | | P=0.80, I^2^=0% | | |  | |
|  | >4, <8 | 17 | | RR=1.19 [1.10, 1.28] | | P<0.00001, I^2^=68% | | |  | |
|  | ≥8 | 13 | | RR=1.12 [1.04, 1.20] | | P<0.00001, I^2^=80% | | |  | |
|  | Age | | | | | | | | | |
|  | Children | 7 | | RR=1.20 [1.06, 1.36] | | P<0.00001, I^2^=83% | | | P=0.83 | |
|  | Adults | 13 | | RR=1.16 [1.07, 1.26] | | P<0.00001, I^2^=80% | | |  | |
|  | Elders | 5 | | RR=1.16 [1.08, 1.25] | | P=0.91, I^2^=0% | | |  | |
|  | Adults+Children | 1 | | RR=1.24 [1.06, 1.44] | | Not available | | |  | |
|  | Adults+Elders | 16 | | RR=1.13 [1.05, 1.21] | | P<0.00001, I^2^=71% | | |  | |
| Length of Hospitalizati-on | Syndrome differentiation and treatment | | | | | | | | | |
|  | With | | 2 | | MD=-2.61 [-3.72, -1.49] | | P=0.05, I^2^=73% | | | **P=0.04** |
|  | Without | | 5 | | MD=-0.91 [-2.08, 0.25] | | P<0.00001, I^2^=91% | | |  |
|  | Flavored quantity of Chinese medicine | | | | | | | | | |
|  | >0, ≤4 | | 2 | | MD=-1.44 [-3.29, 0.41] | | P<0.0001, I^2^=93% | | | P=0.97 |
|  | >4, <8 | | 3 | | MD=-1.22 [-4.02, 1.58] | | P<0.00001, I^2^=97% | | |  |
|  | ≥8 | | 2 | | MD=-1.54 [-2.28, -0.81] | | P=0.51, I^2^=0% | | |  |
|  | Age | | | | | | | | | |
|  | Children | | 2 | | MD=-0.26 [-3.52, 3.01] | | P<0.00001, I^2^=96% | | | P=0.0005 |
|  | Adults+Children | | 1 | | MD=-3.09 [-3.61, -2.57] | | Not available | | |  |
|  | Adults+Elders | | 4 | | MD=-1.47 [-2.44, -0.49] | | P=0.005, I^2^=83% | | |  |
| Resolution time of dyspnea | Flavored quantity of Chinese medicine | | | | | | | | | |
|  | >0, ≤4 | | 2 | | MD=-1.46 [-1.67, -1.25] | | P=0.26, I^2^=23% | | | **P<0.00001** |
|  | >4, <8 | | 3 | | MD=-2.61 [-2.92, -2.30] | | P=0.37, I^2^=0% | | |  |
|  | Age | | | | | | | | | |
|  | Children | | 2 | | MD=-1.46 [-1.67, -1.25] | | P=0.26, I^2^=23% | | | **P<0.00001** |
|  | Adults+Children | | 1 | | MD=-2.52 [-2.87, -2.17] | | Not available | | |  |
|  | Adults+Elders | | 2 | | MD=-2.91 [-3.57, -2.25] | | P=0.33, I^2^=0% | | |  |
| FVC | Syndrome differentiation and treatment | | | | | | | | | |
|  | With | | 2 | | MD=-0.05 [-1.03, 0.93] | | P<0.00001, I^2^=96% | | | P=0.96 |
|  | Without | | 3 | | MD=-0.08 [-0.80, 0.64] | | P<0.00001, I^2^=99% | | |  |
|  | Age | | | | | | | | | |
|  | Adults | | 1 | | MD=0.25 [0.17, 0.33] | | Not available | | | P=0.22 |
|  | Adults+Elders | | 4 | | MD=-0.15 [-0.79, 0.49] | | P<0.00001, I^2^=96% | | |  |
| FEV1 | Syndrome differentiation and treatment | | | | | | | | | |
|  | With | | 1 | | MD=0.65 [0.35, 0.95] | | Not available | | | P=0.57 |
|  | Without | | 3 | | MD=0.51 [0.13, 0.89] | | P<0.00001, I^2^=98% | | |  |
|  | Age | | | | | | | | | |
|  | Adults | | 1 | | MD=0.19 [0.14, 0.24] | | Not available | | | **P<0.00001** |
|  | Adults+Elders | | 3 | | MD=0.64 [0.56, 0.71] | | P=0.72, I^2^=0% | | |  |

## Supplementary Table S4. Summary of results on other outcomes

| **Study ID** | | | **Sample Size (T/C)** | | | | | | | | | | **Estimate effect [95% CI]** | | | | | | | | **P** | | | |  |  |  |  |  |  |
| --- | --- | --- | --- | --- | --- | --- | --- | --- | --- | --- | --- | --- | --- | --- | --- | --- | --- | --- | --- | --- | --- | --- | --- | --- | --- | --- | --- | --- | --- | --- |
| **MXSG+WM vs WM (CRP)** | | | | | | | | | | | | | | | | | | | | | | | | | | |  |  |  |  |
| Cheng F 2018 | | | 29/29 | | | | | | | MD=-3.92 [-4.82, -3.02] | | | | | | | P<0.00001 | | | | | | | |  |  |  |  |  |  |
| Cheng YF 2017 | | | 35/35 | | | | | | | MD=-0.37 [-0.84, 0.11] | | | | | | | P=0.13 | | | | | | | |  |  |  |  |  |  |
| Cheng YF 2021 | | | 40/40 | | | | | | | MD=-2.51 [-3.10, -1.92] | | | | | | | P<0.00001 | | | | | | | |  |  |  |  |  |  |
| Chu Z 2019 | | | 47/47 | | | | | | | MD=-4.16 [-4.89, -3.43] | | | | | | | P<0.00001 | | | | | | | |  |  |  |  |  |  |
| Cui CR 2022 | | | 80/80 | | | | | | | MD=-1.81 [-2.18, -1.44] | | | | | | | P<0.00001 | | | | | | | |  |  |  |  |  |  |
| Dai LF 2018 | | | 55/55 | | | | | | | MD=-2.96 [-3.51, -2.42] | | | | | | | P<0.00001 | | | | | | | |  |  |  |  |  |  |
| Du BT 2020 | | | 150/150 | | | | | | | MD=-3.70 [-4.07, -3.32] | | | | | | | P<0.00001 | | | | | | | |  |  |  |  |  |  |
| Fei XJ 2014 | | | 30/30 | | | | | | | MD=-0.53 [-1.05, -0.02] | | | | | | | P=0.04 | | | | | | | |  |  |  |  |  |  |
| Guo JW 2023 | | | 44/43 | | | | | | | MD=-0.69 [-1.13, -0.26] | | | | | | | P<0.002 | | | | | | | |  |  |  |  |  |  |
| Huo HM 2016 | | | 39/39 | | | | | | | MD=-4.46 [-5.30, -3.61] | | | | | | | P<0.00001 | | | | | | | |  |  |  |  |  |  |
| Hu P 2024 | | | 30/30 | | | | | | | MD=-11.18 [-13.31, -9.05] | | | | | | | P<0.00001 | | | | | | | |  |  |  |  |  |  |
| Hu W 2017 | | | 66/64 | | | | | | | MD=-7.00 [-7.92, -6.07] | | | | | | | P<0.00001 | | | | | | | |  |  |  |  |  |  |
| Li JT 2020 | | | 22/23 | | | | | | | MD=-1.98 [-2.70, -1.25] | | | | | | | P<0.00001 | | | | | | | |  |  |  |  |  |  |
| Li L 2021 | | | 44/44 | | | | | | | MD=-2.39 [-2.94, -1.83] | | | | | | | P<0.00001 | | | | | | | |  |  |  |  |  |  |
| Liu YF 2023 | | | 31/31 | | | | | | | MD=-3.04 [-3.79, -2.30] | | | | | | | P<0.00001 | | | | | | | |  |  |  |  |  |  |
| Liu YH 2016 | | | 25/25 | | | | | | | MD=-4.48 [-5.55, -3.41] | | | | | | | P<0.00001 | | | | | | | |  |  |  |  |  |  |
| Li Y 2020 | | | 42/42 | | | | | | | MD=-3.39 [-4.07, -2.71] | | | | | | | P<0.00001 | | | | | | | |  |  |  |  |  |  |
| Ma FM 2020 | | | 53/53 | | | | | | | MD=-1.40 [-1.82, -0.97] | | | | | | | P<0.00001 | | | | | | | |  |  |  |  |  |  |
| Ma YF 2017 | | | 50/50 | | | | | | | MD=-0.03 [-0.43, 0.36] | | | | | | | P=0.87 | | | | | | | |  |  |  |  |  |  |
| Meng W 2017 | | | 40/40 | | | | | | | MD=-1.95 [-2.49, -1.41] | | | | | | | P<0.00001 | | | | | | | |  |  |  |  |  |  |
| Mo ZH 2022 | | | 25/25 | | | | | | | MD=-0.80 [-1.38, -0.23] | | | | | | | P=0.006 | | | | | | | |  |  |  |  |  |  |
| Ning WJ 2022 | | | 43/43 | | | | | | | MD=-1.36 [-1.83, -0.89] | | | | | | | P<0.00001 | | | | | | | |  |  |  |  |  |  |
| Shen RX 2013 | | | 25/25 | | | | | | | MD=-1.45 [-2.08, -0.82] | | | | | | | P<0.00001 | | | | | | | |  |  |  |  |  |  |
| Song DF 2022 | | | 50/50 | | | | | | | MD=-1.19 [-1.62, -0.77] | | | | | | | P<0.00001 | | | | | | | |  |  |  |  |  |  |
| Sun QQ 2019 | | | 54/54 | | | | | | | MD=-2.41 [-2.91, -1.91] | | | | | | | P<0.00001 | | | | | | | |  |  |  |  |  |  |
| Tang H 2013 | | | 45/45 | | | | | | | MD=-0.43 [-0.84, -0.01] | | | | | | | P=0.05 | | | | | | | |  |  |  |  |  |  |
| Wang CH 2015 | | | 30/30 | | | | | | | MD=-5.69 [-6.86, -4.52] | | | | | | | P<0.00001 | | | | | | | |  |  |  |  |  |  |
| Wang XM | | | 30/30 | | | | | | | MD=-1.02 [-1.56, -0.48] | | | | | | | P=0.0002 | | | | | | | |  |  |  |  |  |  |
| Wu XK 2016 | | | 40/42 | | | | | | | MD=-0.44 [-0.88, -0.00] | | | | | | | P=0.05 | | | | | | | |  |  |  |  |  |  |
| Xiao YL 2021 | | | 37/38 | | | | | | | MD=-0.49 [-0.95, -0.03] | | | | | | | P=0.04 | | | | | | | |  |  |  |  |  |  |
| Xie Y 2023 | | | 48/48 | | | | | | | MD=-2.12 [-2.62, -1.61] | | | | | | | P<0.00001 | | | | | | | |  |  |  |  |  |  |
| Xin DY 2018 (high dose) | | | 29/27 | | | | | | | MD=-1.08 [-1.65, -0.52] | | | | | | | P=0.0002 | | | | | | | |  |  |  |  |  |  |
| Xin DY 2018 (low dose) | | | 27/27 | | | | | | | MD=-0.82 [-1.38, -0.27] | | | | | | | P=0.004 | | | | | | | |  |  |  |  |  |  |
| Xu GL 2020 | | | 30/30 | | | | | | | MD=-2.44 [-3.12, -1.77] | | | | | | | P<0.00001 | | | | | | | |  |  |  |  |  |  |
| Yang JS 2021 | | | 31/31 | | | | | | | MD=-0.30 [-0.80, 0.20] | | | | | | | P=0.24 | | | | | | | |  |  |  |  |  |  |
| Yuan LL 2008 | | | 30/30 | | | | | | | MD=-0.65 [-1.17, -0.13] | | | | | | | P=0.01 | | | | | | | |  |  |  |  |  |  |
| Zhou DH 2016 | | | 40/40 | | | | | | | MD=-0.71 [-1.17, -0.26] | | | | | | | P=0.002 | | | | | | | |  |  |  |  |  |  |
| Zhu J 2022 | | | 51/51 | | | | | | | MD=-13.32 [-15.22, -11.41] | | | | | | | P<0.00001 | | | | | | | |  |  |  |  |  |  |
| Zou P 2018 | | | 30/30 | | | | | | | MD=-1.96 [-2.59, -1.34] | | | | | | | P<0.00001 | | | | | | | |  |  |  |  |  |  |
| **MXSG+WM 1 vs WM 1+WM 2 (CRP)** | | | | | | | | | | | | | | | | | | | | | | | | | | |  |  |  |  |
| Tian XM 2017 | | | 46/44 | | | | | | MD=-4.03 [-4.43, -3.63]] | | | | | P<0.00001 | | | | | | | | |  |  |  |  |  |  |  |  |
| **MXSG vs WM (CRP)** | | | | | | | | | | | | | | | | | | | | | | | | | | |  |  |  |  |
| Chen ZB 2010 | | | 20/20 | | | | | | | | | MD=0.42 [-0.33, 1.17] | | | P=0.27 | | | | | | | | | | | | | | |  |
| **MXSG+WM vs WM (WBC)** | | | | | | | | | | | | | | | | | | | | | | | | | | | |  |  |  |
| Cheng F 2018 | | | | | | 29/29 | | | | | | MD=-2.21 [-3.77, -0.65] | | | | | P=0.006 | | | | | | | |  |  |  |  |  |  |
| Cheng YF 2017 | | | | | | 35/35 | | | | | | MD=-0.70 [-1.20, -0.20] | | | | | P=0.006 | | | | | | | |  |  |  |  |  |  |
| Cheng YF 2021 | | | | | | 40/40 | | | | | | MD=-3.22 [-3.95, -2.49] | | | | | P<0.00001 | | | | | | | |  |  |  |  |  |  |
| Dai LF 2018 | | | | | | 55/55 | | | | | | MD=-1.94 [-2.55, -1.33] | | | | | P<0.00001 | | | | | | | |  |  |  |  |  |  |
| Deng SX 2018 | | | | | | 44/44 | | | | | | MD=-3.37 [-4.70, -2.04] | | | | | P<0.00001 | | | | | | | |  |  |  |  |  |  |
| Fei XJ 2014 | | | | | | 30/30 | | | | | | MD=-0.66 [-1.89, 0.57] | | | | | P=0.29 | | | | | | | |  |  |  |  |  |  |
| He XY 2011 (severe CAP) | | | | | | 10/9 | | | | | | MD=-0.98 [-1.98, 0.02] | | | | | P=0.05 | | | | | | | |  |  |  |  |  |  |
| He XY 2011  (none-severe CAP) | | | | | | 29/11 | | | | | | MD=0.04 [-0.94, 1.02] | | | | | P=0.94 | | | | | | | |  |  |  |  |  |  |
| Hu P 2024 | | | | | | 30/30 | | | | | | MD=-1.68 [-2.27, -1.08] | | | | | P<0.00001 | | | | | | | |  |  |  |  |  |  |
| Huo HM 2016 | | | | | | 39/39 | | | | | | MD=-1.23 [-1.34, -1.12] | | | | | P<0.00001 | | | | | | | |  |  |  |  |  |  |
| Jin ZX 2020 | | | | | | 40/40 | | | | | | MD=-0.32 [-1.21, 0.57] | | | | | P=0.48 | | | | | | | |  |  |  |  |  |  |
| Li JT 2020 | | | | | | 22/23 | | | | | | MD=-2.51 [-3.54, -1.48] | | | | | P<0.00001 | | | | | | | |  |  |  |  |  |  |
| Liu YF 2023 | | | | | | 31/31 | | | | | | MD=-2.35 [-2.43, -2.27] | | | | | P<0.00001 | | | | | | | |  |  |  |  |  |  |
| Liu YH 2016 | | | | | | 25/25 | | | | | | MD=1.34 [1.03, 1.65] | | | | | P<0.00001 | | | | | | | |  |  |  |  |  |  |
| Liu YR 2019 | | | | | | 30/30 | | | | | | MD=-0.25 [-0.65, 0.15] | | | | | P=0.22 | | | | | | | |  |  |  |  |  |  |
| Li Y 2014 | | | | | | 51/49 | | | | | | MD=0.67 [-1.20, 2.54] | | | | | P=0.48 | | | | | | | |  |  |  |  |  |  |
| Mo WS 2021 | | | | | | 27/26 | | | | | | MD=-3.21 [-4.39, -2.03] | | | | | P<0.00001 | | | | | | | |  |  |  |  |  |  |
| Mo ZH 2022 | | | | | | 25/25 | | | | | | MD=-1.74 [-2.51, -0.97] | | | | | P<0.00001 | | | | | | | |  |  |  |  |  |  |
| Ning WJ 2022 | | | | | | 43/43 | | | | | | MD=-0.67 [-1.25, -0.09] | | | | | P=0.02 | | | | | | | |  |  |  |  |  |  |
| Shen AY 2014 | | | | | | 39/38 | | | | | | MD=-2.14 [-3.49, -0.79] | | | | | P=0.002 | | | | | | | |  |  |  |  |  |  |
| Sun K 2022 | | | | | | 32/32 | | | | | | MD=-2.14 [-3.61, -0.67] | | | | | P=0.004 | | | | | | | |  |  |  |  |  |  |
| Sun QS 2015 | | | | | | 65/65 | | | | | | MD=-2.55 [-3.21, -1.89] | | | | | P<0.00001 | | | | | | | |  |  |  |  |  |  |
| Tang H 2013 | | | | | | 45/45 | | | | | | MD=-1.04 [-1.78, -0.30] | | | | | P=0.006 | | | | | | | |  |  |  |  |  |  |
| Wang XM 2024 | | | | | | 30/30 | | | | | | MD=-1.73 [-2.32, -1.13] | | | | | P<0.00001 | | | | | | | |  |  |  |  |  |  |
| Xiao YL 2021 | | | | | | 37/38 | | | | | | MD=-1.31 [-2.38, -0.24] | | | | | P=0.02 | | | | | | | |  |  |  |  |  |  |
| Xie Y 2023 | | | | | | 48/48 | | | | | | MD=-2.38 [-3.03, -1.73] | | | | | P<0.00001 | | | | | | | |  |  |  |  |  |  |
| Xin DY 2018 (high dose) | | | | | | 29/27 | | | | | | MD=-1.03 [-2.11, 0.05] | | | | | P=0.06 | | | | | | | |  |  |  |  |  |  |
| Xin DY 2018 (low dose) | | | | | | 27/27 | | | | | | MD=-1.33 [-2.33, -0.33] | | | | | P=0.009 | | | | | | | |  |  |  |  |  |  |
| Xu GL 2016 | | | | | | 30/30 | | | | | | MD=-1.70 [-4.40, 1.00] | | | | | P=0.22 | | | | | | | |  |  |  |  |  |  |
| Xu GL 2020 | | | | | | 30/30 | | | | | | MD=-1.49 [-2.12, -0.86] | | | | | P<0.00001 | | | | | | | |  |  |  |  |  |  |
| Yang JS 2021 | | | | | | 31/31 | | | | | | MD=-2.53 [-3.24, -1.82] | | | | | P<0.00001 | | | | | | | |  |  |  |  |  |  |
| Yuan LL 2008 | | | | | | 30/30 | | | | | | MD=-1.39 [-2.51, -0.27] | | | | | P=0.01 | | | | | | | |  |  |  |  |  |  |
| Zhou DH 2016 | | | | | | 40/40 | | | | | | MD=-1.41 [-2.88, 0.06] | | | | | P=0.06 | | | | | | | |  |  |  |  |  |  |
| Zhu J 2022 | | | | | | 51/51 | | | | | | MD=0.07 [-0.01, 0.15] | | | | | P=0.09 | | | | | | | |  |  |  |  |  |  |
| Zou P 2018 | | | | | | 30/30 | | | | | | MD=-3.52 [-5.50, -1.54] | | | | | P=0.0005 | | | | | | | |  |  |  |  |  |  |
| **MXSG+WM 1 vs WM 1+WM 2 (WBC)** | | | | | | | | | | | | | | | | | | | | | | | | | | | |  |  |  |
| Tian XM 2017 | | | | | 46/44 | | | | | | | | | MD=-2.15 [-3.43, -0.87] | | | | | P=0.001 | | | | | | | | |  |  |  |
| **MXSG vs WM (WBC)** | | | | | | | | | | | | | | | | | | | | | | | | | | | |  |  |  |
| Chen ZB 2010 | | | | | 20/20 | | | | | | | | | MD=-0.29 [-1.46, 0.88] | | | | | | | P=0.63 | |  |  |  |  |  |  |  |  |
| **MXSG+WM vs WM (PCT)** | | | | | | | | | | | | | | | | | | | | | | | | | | | |  |  |  |
| Chen AG 2018 | | | | 27/26 | | | | | MD=-1.88 [-2.53, -1.23] | | | | | | | | | | | | | P<0.00001 | | | | | | | |  |
| Cheng F 2018 | | | | 29/29 | | | | | MD=-0.80 [-1.34, -0.26] | | | | | | | | | | | | | P=0.003 | | | | | | | |  |
| Deng SX 2018 | | | | 44/44 | | | | | MD=-1.84 [-2.35, -1.34] | | | | | | | | | | | | | P<0.00001 | | | | | | | |  |
| Du BT 2020 | | | | 150/150 | | | | | MD=-1.73 [-2.00, -1.47] | | | | | | | | | | | | | P<0.00001 | | | | | | | |  |
| Guo JW 2023 | | | | 44/43 | | | | | MD=-0.59 [-1.02, -0.16] | | | | | | | | | | | | | P=0.007 | | | | | | | |  |
| Hu P 2024 | | | | 30/30 | | | | | MD=-3.53 [-4.36, -2.70] | | | | | | | | | | | | | P<0.00001 | | | | | | | |  |
| Hu W 2017 | | | | 66/64 | | | | | MD=-5.05 [-5.77, -4.34] | | | | | | | | | | | | | P<0.00001 | | | | | | | |  |
| Li JT 2020 | | | | 22/23 | | | | | MD=-3.48 [-4.44, -2.53] | | | | | | | | | | | | | P<0.00001 | | | | | | | |  |
| Li L 2021 | | | | 44/44 | | | | | MD=-0.89 [-1.33, -0.45] | | | | | | | | | | | | | P<0.0001 | | | | | | | |  |
| Li Y 2014 | | | | 51/49 | | | | | MD=-0.08 [-0.47, 0.31] | | | | | | | | | | | | | P=0.69 | | | | | | | |  |
| Liu YF 2023 | | | | 31/31 | | | | | MD=-2.76 [-3.47, -2.06] | | | | | | | | | | | | | P<0.00001 | | | | | | | |  |
| Liu YR 2019 | | | | 30/30 | | | | | MD=-0.63 [-1.15, -0.11] | | | | | | | | | | | | | P=0.02 | | | | | | | |  |
| Ma FM 2020 | | | | 53/53 | | | | | MD=-0.73 [-1.12, -0.33] | | | | | | | | | | | | | P=0.0003 | | | | | | | |  |
| Mo WS 2021 | | | | 27/26 | | | | | MD=-2.55 [-3.29, -1.81] | | | | | | | | | | | | | P<0.00001 | | | | | | | |  |
| Ning WJ 2022 | | | | 43/43 | | | | | MD=-1.99 [-2.51, -1.47] | | | | | | | | | | | | | P<0.00001 | | | | | | | |  |
| Shen AY 2014 | | | | 39/38 | | | | | | MD=-2.60 [-3.21, -1.99] | | | | | | | | | | | | P<0.00001 | | | | | | | | |
| Shen RX 2013 | | | | 32/32 | | | | | | MD=-1.34 [-1.89, -0.80] | | | | | | | | | | | | P<0.00001 | | | | | | | | |
| Sun K 2022 | | | | 32/32 | | | | | | MD=-2.63 [-3.31, -1.95] | | | | | | | | | | | | | P<0.00001 | | | | | | | |
| Sun QQ 2019 | | | | 54/54 | | | | | | MD=-0.80 [-1.19, -0.41] | | | | | | | | | | | | | P<0.0001 | | | | | | | |
| Sun QS 2015 | | | | 65/65 | | | | | | MD=-14.23 [-16.02, -12.44] | | | | | | | | | | | | | P<0.00001 | | | | | | | |
| Wang CH 2015 | | | | 30/30 | | | | | | MD=-2.86 [-3.59, -2.13] | | | | | | | | | | | | | | P<0.00001 | | | | | |  |
| Wang XM 2024 | | | | 30/30 | | | | | | MD=-3.54[-4.36, -2.71] | | | | | | | | | | | | P<0.00001 | | | | | |  |  |  |
| Xie Y 2023 | | | | 48/48 | | | | | | MD=-1.44 [-1.89, -0.99] | | | | | | | | | | | | P<0.00001 | | | | | |  |  |  |
| Xu GL 2016 | | | | 30/30 | | | | | | MD=-1.45 [-2.02, -0.87] | | | | | | | | | | | | P<0.00001 | | | | | |  |  |  |
| Xu GL 2020 | | | | 30/30 | | | | | | MD=-2.75 [-3.47, -2.03] | | | | | | | | | | | | P<0.00001 | | | | | |  |  |  |
| Yang JS 2021 | | | | 31/31 | | | | | | MD=-0.45 [-0.96, 0.05] | | | | | | | | | | | | P=0.08 | | | | | |  |  |  |
| Zhu J 2022 | | | | 51/51 | | | | | | MD=-1.56 [-2.00, -1.11] | | | | | | | | | | | | P<0.00001 | | | | | |  |  |  |
| Zou P 2018 | | | | 30/30 | | | | | | MD=-2.45 [-3.12, -1.77] | | | | | | | | | | | | P<0.00001 | | | | | |  |  |  |
| **MXSG+WM 1 vs WM1+WM 2 (PCT)** | | | | | | | | | | | | | | | | | | | | | | | | | | | |  |  |  |
| Tian XM 2017 | | | 46/44 | | | | | | | MD=-0.19 [-0.29, -0.09] | | | | | | | | | | | | P=0.0003 | | | |  |  |  |  |  |
| **MXSG+WM 1 vs WM1+WM 2 (Absorption Time of Lung Inflammation )** | | | | | | | | | | | | | | | | | | | | | | | | | |  |  |  |  |  |
| Tian XM 2017 | | | 46/44 | | | | | | | MD=-4.17 [-8.43, 0.09] | | | | | | | | | | | P=0.06 | | |  |  |  |  |  |  |  |
| \| **MXSG+WM vs WM** **(Improvement Rate of Chest Radiograph)** \| \| \| \| \| \| \| \| \| \| --- \| --- \| --- \| --- \| --- \| --- \| --- \| --- \| --- \| \| Cheng F 2018 \| \| 29/29 \| \| RR=1.23 [0.98, 1.54] \| \| \| \| \| \| P=0.08 \| \| Cheng YF 2017 \| \| 35/35 \| \| RR=1.00 [0.95, 1.06] \| \| \| \| \| \| P=1.00 \| \| Chu Z 2019 \| \| 47/47 \| \| RR=1.22 [1.04, 1.43] \| \| \| \| \| \| P=0.02 \| \| Cui CR 2022 \| \| 80/80 \| \| RR=1.13 [1.02, 1.25] \| \| \| \| \| \| P=0.02 \| \| Dai LF 2018 \| \| 55/55 \| \| RR=1.21 [1.04, 1.41] \| \| \| \| \| \| P=0.02 \| \| Dong LH 2023 \| \| 51/52 \| \| RR=1.29 [1.02, 1.62] \| \| \| \| \| \| P=0.03 \| \| Gao GL 2022 \| \| 47/47 \| \| RR=1.26 [1.05, 1.51] \| \| \| \| \| \| P=0.01 \| \| Huo HM 2016 \| \| 39/39 \| \| RR=1.20 [0.99, 1.46] \| \| \| \| \| \| P=0.07 \| \| Hu W 2017 \| \| 66/64 \| \| RR=1.03 [0.98, 1.09] \| \| \| \| \| \| P=0.24 \| \| Jin ZX 2020 \| \| 40/40 \| \| RR=1.04 [0.76, 1.42] \| \| \| \| \| \| P=0.81 \| \| Li HN 2006 \| \| 39/40 \| \| RR=1.31 [1.07, 1.61] \| \| \| \| \| \| P=0.01 \| \| Li JT 2020 \| \| 22/23 \| \| RR=1.23 [0.93, 1.62] \| \| \| \| \| \| P=0.14 \| \| Li L 2021 \| \| 44/44 \| \| RR=1.37 [1.10, 1.70] \| \| \| \| \| \| P=0.005 \| \| Liu BH 2018 \| \| 34/34 \| \| RR=1.13 [0.99, 1.29] \| \| \| \| \| \| P=0.07 \| \| Liu HY 2016 \| \| 30/30 \| \| RR=1.11 [0.97, 1.27] \| \| \| \| \| \| P=0.13 \| \| Liu YH 2016 \| \| 25/25 \| \| RR=1.14 [0.95, 1.38] \| \| \| \| \| \| P=0.17 \| \| Liu YR 2011 \| \| 30/30 \| \| RR=1.16 [0.98, 1.38] \| \| \| \| \| \| P=0.09 \| \| Li YE 2020 \| \| 37/37 \| \| RR=1.46 [1.14, 1.87] \| \| \| \| \| \| P=0.003 \| \| Ma FM 2020 \| \| 53/53 \| \| RR=1.16 [1.01, 1.32] \| \| \| \| \| \| P=0.03 \| \| Ma WJ 2015 \| \| 29/29 \| \| RR=1.13 [0.93, 1.37] \| \| \| \| \| \| P=0.23 \| \| Mo WS 2021 \| \| 27/26 \| \| RR=1.32 [1.03, 1.68] \| \| \| \| \| \| P=0.03 \| \| Mo ZH 2022 \| \| 25/25 \| \| RR=1.38 [0.99, 1.91] \| \| \| \| \| \| P=0.06 \| \| Ning WJ 2022 \| \| 43/43 \| \| RR=1.21 [1.02, 1.43] \| \| \| \| \| \| P=0.03 \| \| Shen RX 2013 \| \| 32/32 \| \| RR=1.33 [0.93, 1.92] \| \| \| \| \| \| P=0.12 \| \| Song DF 2022 \| \| 50/50 \| \| RR=1.11 [1.01, 1.23] \| \| \| \| \| \| P=0.04 \| \| Sun QQ 2019 \| \| 54/54 \| \| RR=1.24 [1.06, 1.44] \| \| \| \| \| \| P=0.006 \| \| Sun QS 2015 \| \| 65/65 \| \| RR=1.13 [1.00, 1.27] \| \| \| \| \| \| P=0.04 \| \| Su QZ 2016 \| \| 30/30 \| \| RR=1.08 [0.91, 1.28] \| \| \| \| \| \| P=0.39 \| \| Su SD 2016 \| \| 40/40 \| \| RR=1.24 [0.93, 1.66] \| \| \| \| \| \| P=0.15 \| \| Tang H 2013 \| \| 45/45 \| \| RR=1.24 [1.03, 1.48] \| \| \| \| \| \| P=0.02 \| \| Tian YH 2013 \| \| 38/38 \| \| RR=1.20 [1.00, 1.44] \| \| \| \| \| \| P=0.05 \| \| Wang CM 2014 \| \| 25/25 \| \| RR=1.16 [0.89, 1.51] \| \| \| \| \| \| P=0.28 \| \| Wang JJ 2017 \| \| 28/28 \| \| RR=1.23 [1.00, 1.51] \| \| \| \| \| \| P=0.05 \| \| Wu TF 2013 \| \| 63/60 \| \| RR=1.20 [1.03, 1.39] \| \| \| \| \| \| P=0.02 \| \| Xiao YL 2021 \| \| 37/38 \| \| RR=1.00 [0.95, 1.05] \| \| \| \| \| \| P=1.00 \| \| Xu GL 2016 \| \| 30/30 \| \| RR=1.42 [1.06, 1.91] \| \| \| \| \| \| P=0.02 \| \| Yang HG 2014 \| \| 44/44 \| \| RR=1.21 [1.01, 1.44] \| \| \| \| \| \| P=0.04 \| \| Yang J 2020 \| \| 30/30 \| \| RR=1.21 [1.00, 1.46] \| \| \| \| \| \| P=0.05 \| \| Yuan LL 2008 \| \| 30/30 \| \| RR=1.03 [0.94, 1.13] \| \| \| \| \| \| P=0.47 \| \| Zhang JJ 2020 \| \| 40/40 \| \| RR=1.00 [0.93, 1.07] \| \| \| \| \| \| P=1.00 \| \| Zhou DH 2016 \| \| 40/40 \| \| RR=1.00 [0.93, 1.07] \| \| \| \| \| \| P=1.00 \| \| Zhou YH 2015 \| \| 45/45 \| \| RR=1.16 [1.00, 1.35] \| \| \| \| \| \| P=0.05 \| \| **MXSG+WM1 vs WM1 +WM2 (Improvement Rate of Chest Radiograph)** \| \| \| \| \| \| \| \| \| \| \| \| \| Tian XM 2017 \| 46/44 \| \| RR=1.05 [0.96, 1.15] \| \| \| \| \| P=0.29 \| \| \| \| \| \| \| **MXSG vs placebo (Improvement Rate of Chest Radiograph)** \| \| \| \| \| \| \| \| \| \| \| \| \| \| \| Zheng YJ 2022 \| 36/35 \| \| RR=1.04 [0.87, 1.24] \| \| P=0.69 \| \| \| \| \| \| \| \| \| **MXSG vs WM (Improvement Rate of Chest Radiograph)** \| \| \| \| \| \| \| \| \| \| \| \| \| \| Chen ZB 2010 \| 20/20 \| \| RR=1.00 [0.91, 1.10] \| \| P=1.00 \| \| \| \| \| \| \| \| \| **MXSG+WM vs WM (FVC)** \| \| \| \| \| \| \| \| \| \| \| \| \| \| Cheng YF 2021 \| 40/40 \| \| MD=0.21 [-0.58, 1.00] \| \| P=0.60 \| \| \| \| \| \| \| \| \| Cui CR 2022 \| 80/80 \| \| MD=0.25 [0.17, 0.33] \| \| P<0.00001 \| \| \| \| \| \| \| \| \| Kong FH 2023 \| 60/60 \| \| MD=0.44 [0.23, 0.65] \| \| P<0.00001 \| \| \| \| \| \| \| \| \| Liu Y 2019 \| 40/40 \| \| MD=-0.56 [-0.89, -0.23] \| \| \| \| P=0.001 \| \| \| \| \| \| \| \| \| Ni CY 2023 \| 40/40 \| \| MD=-0.61 [-0.72,-0.50] \| \| \| \| P<0.00001 \| \| \| \| \| \| \| \| \| **MXSG+WM vs WM (PEF)** \| \| \| \| \| \| \| \| \| \| \| \| \| \| Fang F 2022 \| 49/49 \| \| RR=14.00 [9.37, 18.63] \| \| \| P<0.00001 \| \| \| \| \| \| \| \| \| \| \| Kong FH 2023 \| 60/0 \| \| RR=0.35 [0.20, 0.50] \| \| P<0.00001 \| \| \| \| \| \| \| \|   **T** treatment group, **C** control group, **RR** risk ratio, **CI** confidence interval, **CRP** C-reactive protein, **WBC** white blood cell, **PCT** procalcitonin, **FVC** forced vital capacity, **PEF** peak expiratory flow | | | | | | | | | | | | | | | | | | | | | | | | | | | |  |  |  |

# Supplementary Files

## Supplementary File S1. Search strategy

**CNKI**

TKA=('社区获得性肺炎' + 'CAP' + '肺炎' + '肺部炎症' + '肺部感染') AND (FT=('麻黄' * '杏仁' * '石膏' * '甘草') OR TKA=('麻杏石甘汤' + '麻杏石甘' + '麻杏甘石' + '麻杏甘石汤' + '麻黄杏仁甘草石膏' + '麻黄杏仁甘草石膏汤' + '连花清瘟' + '连花清瘟胶囊' + '连花清瘟颗粒' + '清肺消炎' + '清肺消炎丸' + '银黄清肺' + '银黄清肺胶囊' + '清咳平喘' + '清咳平喘颗粒' + '解毒清肺' + '解毒清肺合剂' + '清肺解毒' + '清肺解毒方' + '五虎' + '五虎汤' + '陷麻消痰' + '陷麻消痰汤')) AND TKA='随机'

n=1333

**Wanfang**

主题:("社区获得性肺炎" or "CAP" or "肺炎" or "肺部炎症" or "肺部感染") and (全部:("麻黄" and "杏仁" and "甘草" and "石膏") or 主题:("麻杏石甘汤" or "麻杏石甘" or "麻杏甘石" or "麻杏甘石汤" or "麻黄杏仁甘草石膏" or "麻黄杏仁甘草石膏汤" or "连花清瘟" or "连花清瘟胶囊" or "连花清瘟颗粒" or "清肺消炎" or "清肺消炎丸" or "银黄清肺" or "银黄清肺胶囊" or "清咳平喘" or "清咳平喘颗粒" or "解毒清肺" or "解毒清肺合剂" or "清肺解毒" or "清肺解毒方" or "五虎" or "五虎汤" or "陷麻消痰" or "陷麻消痰汤")) and 主题: "随机"

n=880

**VIP**

R=("社区获得性肺炎" + "CAP" + "肺炎" + "肺部炎症" + "肺部感染") AND (U=("麻黄" * "杏仁" * "甘草" * "石膏") OR R=("麻杏石甘" + "麻杏石甘汤" + "麻杏甘石" + "麻杏甘石汤" + "麻黄杏仁甘草石膏" + "麻黄杏仁甘草石膏汤" + "连花清瘟" + "连花清瘟胶囊" + "连花清瘟颗粒" + "清肺消炎" + "清肺消炎丸" + "银黄清肺" + "银黄清肺胶囊" + "清咳平喘" + "清咳平喘颗粒" + "解毒清肺" + "解毒清肺合剂" + "清肺解毒" + "清肺解毒方" + "五虎" + "五虎汤" + "陷麻消痰" + "陷麻消痰汤")) AND R="随机"

M=("社区获得性肺炎" + "CAP" + "肺炎" + "肺部炎症" + "肺部感染") AND (U=("麻黄" * "杏仁" * "甘草" * "石膏") OR M=("麻杏石甘" + "麻杏石甘汤" + "麻杏甘石" + "麻杏甘石汤" + "麻黄杏仁甘草石膏" + "麻黄杏仁甘草石膏汤" + "连花清瘟" + "连花清瘟胶囊" + "连花清瘟颗粒" + "清肺消炎" + "清肺消炎丸" + "银黄清肺" + "银黄清肺胶囊" + "清咳平喘" + "清咳平喘颗粒" + "解毒清肺" + "解毒清肺合剂" + "清肺解毒" + "清肺解毒方" + "五虎" + "五虎汤" + "陷麻消痰" + "陷麻消痰汤")) AND M="随机"

n=783

**Yiigle**

(标题=社区获得性肺炎 OR CAP OR 肺炎 OR 肺部炎症 OR 肺部感染) AND ((所有=麻黄 AND 杏仁 AND 石膏 AND 甘草) OR (标题=麻杏石甘 OR 麻杏石甘汤 OR 麻杏甘石 OR 麻杏甘石汤 OR 麻黄杏仁甘草石膏 OR 麻黄杏仁甘草石膏汤 OR 连花清瘟 OR 连花清瘟胶囊 OR 连花清瘟颗粒 OR 清肺消炎 OR 清肺消炎丸 OR 银黄清肺 OR 银黄清肺胶囊 OR 清咳平喘 OR 清咳平喘颗粒 OR 解毒清肺 OR 解毒清肺合剂 OR 清肺解毒 OR 清肺解毒方 OR 五虎 OR 五虎汤 OR 陷麻消痰 OR 陷麻消痰汤)) AND 标题=随机

(关键词=社区获得性肺炎 OR CAP OR 肺炎 OR 肺部炎症 OR 肺部感染) AND ((所有=麻黄 AND 杏仁 AND 石膏 AND 甘草) OR (关键词=麻杏石甘 OR 麻杏石甘汤 OR 麻杏甘石 OR 麻杏甘石汤 OR 麻黄杏仁甘草石膏 OR 麻黄杏仁甘草石膏汤 OR 连花清瘟 OR 连花清瘟胶囊 OR 连花清瘟颗粒 OR 清肺消炎 OR 清肺消炎丸 OR 银黄清肺 OR 银黄清肺胶囊 OR 清咳平喘 OR 清咳平喘颗粒 OR 解毒清肺 OR 解毒清肺合剂 OR 清肺解毒 OR 清肺解毒方 OR 五虎 OR 五虎汤 OR 陷麻消痰 OR 陷麻消痰汤)) AND 关键词=随机

(摘要=社区获得性肺炎 OR CAP OR 肺炎 OR 肺部炎症 OR 肺部感染) AND ((所有=麻黄 AND 杏仁 AND 石膏 AND 甘草) OR (摘要=麻杏石甘 OR 麻杏石甘汤 OR 麻杏甘石 OR 麻杏甘石汤 OR 麻黄杏仁甘草石膏 OR 麻黄杏仁甘草石膏汤 OR 连花清瘟 OR 连花清瘟胶囊 OR 连花清瘟颗粒 OR 清肺消炎 OR 清肺消炎丸 OR 银黄清肺 OR 银黄清肺胶囊 OR 清咳平喘 OR 清咳平喘颗粒 OR 解毒清肺 OR 解毒清肺合剂 OR 清肺解毒 OR 清肺解毒方 OR 五虎 OR 五虎汤 OR 陷麻消痰 OR 陷麻消痰汤)) AND 摘要=随机

n=8

**SinoMed**

((( "麻杏石甘"[常用字段:智能] OR "麻杏石甘汤"[常用字段:智能] OR "麻杏甘石"[常用字段:智能] OR "麻杏甘石汤"[常用字段:智能] OR "麻黄杏仁甘草石膏"[常用字段:智能] OR "麻黄杏仁甘草石膏汤"[常用字段:智能]) OR( "连花清瘟"[常用字段:智能] OR "连花清瘟胶囊"[常用字段:智能] OR "连花清瘟颗粒"[常用字段:智能] OR "清肺消炎"[常用字段:智能] OR "清肺消炎丸"[常用字段:智能] OR "银黄清肺"[常用字段:智能] OR "银黄清肺胶囊"[常用字段:智能] OR "清咳平喘"[常用字段:智能] OR "清咳平喘颗粒"[常用字段:智能]) OR( "解毒清肺"[常用字段:智能] OR "解毒清肺合剂"[常用字段:智能] OR "清肺解毒"[常用字段:智能] OR "清肺解毒方"[常用字段:智能] OR "五虎"[常用字段:智能] OR "五虎汤"[常用字段:智能] OR "陷麻消痰"[常用字段:智能] OR "陷麻消痰汤"[常用字段:智能])) OR ("麻黄"[全部字段:智能] AND "杏仁"[全部字段:智能] AND "石膏"[全部字段:智能] AND "甘草"[全部字段:智能])) AND ("随机"[常用字段:智能]) AND ("社区获得性肺炎"[常用字段:智能] OR "CAP"[常用字段:智能] OR "肺炎"[常用字段:智能] OR "肺部炎症"[常用字段:智能] OR "肺部感染"[常用字段:智能])

n=716

**PubMed**

(((((("community acquired pneumonia"[Title/Abstract]) OR (CAP[Title/Abstract])) OR (pneumoni*[Title/Abstract])) OR ("lung inflammation"[Title/Abstract])) OR ("pulmonary inflammation"[Title/Abstract])) AND ((((((((Mahuang) OR ("Ma Huang")) OR ("Ephedrae herba")) OR ("Ephedra sinica Stapf")) AND ((((Xingren) OR ("Xing Ren")) OR ("Apricot kernel")) OR ("Semen Armeniacae Amarum"))) AND ((((Shigao) OR ("Shi Gao")) OR (Gypsum)) OR ("Gypsum Fibrosum"))) AND ((((Gancao) OR ("Gan Cao")) OR (Liquorice)) OR ("Glycyrrhiza uralensis Fisch"))) OR ((((((((((((((((((("Maxing Shigan"[Title/Abstract]) OR ("Maxing Ganshi"[Title/Abstract])) OR ("Ma Xing Shi Gan"[Title/Abstract])) OR ("Ma Xing Gan Shi"[Title/Abstract])) OR (Maxingshigan[Title/Abstract])) OR (Maxingganshi[Title/Abstract])) OR (MXSGT[Title/Abstract])) OR ("mahuang xingren gancao shigao"[Title/Abstract])) OR ("Lianhua Qingwen"[Title/Abstract])) OR (Lianhuaqingwen[Title/Abstract])) OR ("Lian Hua Qing Wen"[Title/Abstract])) OR ("Qingfei Xiaoyan"[Title/Abstract])) OR ("Yinhuang Qingfei"[Title/Abstract])) OR ("Qingke Pingchuan"[Title/Abstract])) OR ("Detoxification Qingfei Mixture"[Title/Abstract])) OR ("Detoxification-clearing Lung Mixture"[Title/Abstract])) OR ("Qingfei Jiedu"[Title/Abstract])) OR (Wuhu[Title/Abstract])) OR ("XianMa Xiaotan"[Title/Abstract])))) AND (random*[Title/Abstract])

n=22

**Cochrane Library**

Search Name: CAP

Date Run: 2025/1/20 15:08:08

Comment:

ID Search Hits

#1 ("community acquired pneumonia" or CAP or pneumoni* or "lung inflammation" or "pulmonary inflammation"):ti,ab,kw (Word variations have been searched)

#2 (Mahuang or "Ma Huang" or "Ephedrae herba" or "Ephedra sinica Stapf") (Word variations have been searched)

#3 (Xingren or "Xing Ren" or "Apricot kernel" or "Semen Armeniacae Amarum") (Word variations have been searched)

#4 (Shigao or "Shi Gao" or Gypsum or "Gypsum Fibrosum") (Word variations have been searched)

#5 (Gancao or "Gan Cao" or Liquorice or "Glycyrrhiza uralensis Fisch") (Word variations have been searched)

#6 #2 and #3 and #4 and #5

#7 ("Maxing Shigan" or "Maxing Ganshi" or "Ma Xing Shi Gan" or "Ma Xing Gan Shi" or "Maxingshigan" or "Maxingganshi" or MXSGT or "mahuang xingren gancao shigao" or "Lianhua Qingwen" or "Lianhuaqingwen" or "Lian Hua Qing Wen" or "Qingfei Xiaoyan" or "Yinhuang Qingfei" or "Qingke Pingchuan" or "Detoxification Qingfei Mixture" or "Detoxification-clearing Lung Mixture" or "Qingfei Jiedu" or "Wuhu" or "Xianma Xiaoyan"):ti,ab,kw (Word variations have been searched)

#8 (random*):ti,ab,kw (Word variations have been searched)

#9 #6 or #7

#10 #1 and #8 and #9

n=30

**Embase**

('community acquired pneumonia':ti OR cap:ti OR pneumoni*:ti OR 'lung inflammation':ti OR 'pulmonary inflammation':ti) AND ((xingren OR 'xing ren' OR 'apricot kernel' OR 'semen armeniacae amarum') AND (mahuang OR 'ma huang' OR 'ephedrae herba' OR 'ephedra sinica stapf') AND (shigao OR 'shi gao' OR gypsum OR 'gypsum fibrosum') AND (gancao OR 'gan cao' OR liquorice OR 'glycyrrhiza uralensis fisch') OR 'maxing shigan':ti,ab,kw OR 'maxing ganshi':ti,ab,kw OR 'ma xing shi gan':ti,ab,kw OR 'ma xing gan shi':ti,ab,kw OR maxingshigan:ti,ab,kw OR maxingganshi:ti,ab,kw OR mxsgt:ti,ab,kw OR 'mahuang xingren gancao shigao':ti,ab,kw OR 'lianhua qingwen':ti,ab,kw OR lianhuaqingwen:ti,ab,kw OR 'lian hua qing wen':ti,ab,kw OR 'qingfei xiaoyan':ti,ab,kw OR 'yinhuang qingfei':ti,ab,kw OR 'qingke pingchuan':ti,ab,kw OR 'detoxification qingfei mixture':ti,ab,kw OR 'detoxification-clearing lung mixture':ti,ab,kw OR 'qingfei jiedu':ti,ab,kw OR wuhu:ti,ab,kw OR 'xianma xiaotan':ti,ab,kw) AND random*:ti,ab,kw

n=32
